# A Systematic Review of Carcinogenic Outcomes and Potential Mechanisms from Exposure to 2,4-D and MCPA in the Environment

**DOI:** 10.1155/2013/371610

**Published:** 2013-02-26

**Authors:** Katherine von Stackelberg

**Affiliations:** ^1^E Risk Sciences, LLP, 12 Holton Street, Allston, MA 02134, USA; ^2^Harvard Center for Risk Analysis, 401 Park Drive, Landmark 404J, Boston, MA 02215, USA

## Abstract

Chlorophenoxy compounds, particularly 2,4-dichlorophenoxyacetic acid (2,4-D) and 4-chloro-2-methylphenoxy)acetic acid (MCPA), are amongst the most widely used herbicides in the United States for both agricultural and residential applications. Epidemiologic studies suggest that exposure to 2,4-D and MCPA may be associated with increased risk non-Hodgkins lymphoma (NHL), Hodgkin's disease (HD), leukemia, and soft-tissue sarcoma (STS). Toxicological studies in rodents show no evidence of carcinogenicity, and regulatory agencies worldwide consider chlorophenoxies as not likely to be carcinogenic or unclassifiable as to carcinogenicity. This systematic review assembles the available data to evaluate epidemiologic, toxicological, pharmacokinetic, exposure, and biomonitoring studies with respect to key cellular events noted in disease etiology and how those relate to hypothesized modes of action for these constituents to determine the plausibility of an association between exposure to environmentally relevant concentrations of 2,4-D and MCPA and lymphohematopoietic cancers. The combined evidence does not support a genotoxic mode of action. Although plausible hypotheses for other carcinogenic modes of action exist, a comparison of biomonitoring data to oral equivalent doses calculated from bioassay data shows that environmental exposures are not sufficient to support a causal relationship. Genetic polymorphisms exist that are known to increase the risk of developing NHL. The potential interaction between these polymorphisms and exposures to chlorophenoxy compounds, particularly in occupational settings, is largely unknown.

## 1. Introduction

**Figure 1:**
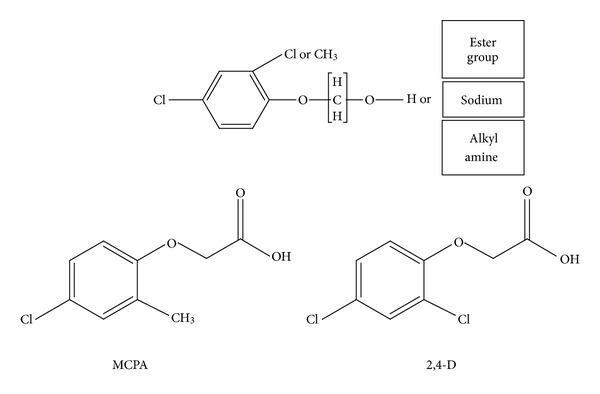
General chemical structure of chlorophenoxy compounds and 2,4-D and MCPA.

The chlorophenoxy herbicides MCPA and 2,4-D are registered for a range of agricultural and residential uses focused on control of postemergent broadleaf weeds. Since 2001, 2,4-D has been the most commonly used herbicide in the residential market at 8 to 11 million pounds annually and is the seventh most commonly used herbicide in the agricultural market ranging from 24 to 30 million pounds annually (http://www.epa.gov/pesticides/pestsales/07pestsales/usage2007_2.htm#3_5/). MCPA is used less, falling within the top 25 compounds used residentially and agriculturally, but is a closely related compound. Phenoxy herbicides act by simulating the action of natural hormones to produce uncoordinated plant growth. Their action is selective as they are toxic to dicotyledonous but not monocotyledonous plants. The physical properties of chlorophenoxy compounds can vary greatly according to formulation. For instance, as alkali salts they are highly water soluble (can be formulated as aqueous solutions), whereas as simple esters they demonstrate low water solubility and are more lipophilic (generally formulated as emulsifiable concentrates). The acid is the parent compound, but a number of formulations in use contain the more water-soluble amine salts or the ester derivatives, which are readily dissolved in an organic solvent.  Figure 1 shows the general chemical structure of the chlorophenoxy herbicides, together with the structures of the parent compounds MCPA and 2,4-D.

A series of rodent bioassays submitted to the US EPA in support of pesticide registration have found no carcinogenic treatment-related effects for either MCPA [1–5] or 2,4-D [6–8]. Regulatory agencies in their evaluations of these two constituents have found them unlikely to be human carcinogens [3, 4] or unclassifiable as to carcinogenicity [8–10] while the World Health Organization (WHO) has concluded that 2,4-D and its salts and esters are not genotoxic, specifically, and the toxicity of the salts and esters of 2,4-D is comparable to that of the acid [11]. However, a number of epidemiologic studies have found positive associations between some measure of exposure either to chlorophenoxy compounds and/or MCPA and/or 2,4-D in particular and an increased risk of some lymphohematopoietic cancers, primarily non-Hodgkins lymphoma (NHL) [12–14], but also Hodgkin's disease (HD), soft-tissue sarcoma (STS), and to a lesser extent, leukemia, while others have found no associations [15, 16] or only in combination with other compounds or with multiple chlorophenoxys [17]. 

Under the assumption that the epidemiologic studies reveal a potential association between exposure and outcome, there must be a series of cellular events by which exposure to chlorophenoxy compounds is causally related to these carcinogenic outcomes. The toxicological studies are equivocal. While the traditional *in vivo* rodent assays are all negative for tumorigenic responses, and a number of *in vivo* and *in vitro* studies of potential mutagenicity and clastogenicity are negative [18–20], a number of other *in vitro* studies have shown weakly positive responses for chromosomal aberrations, sister chromatid exchange [SCE], and increased micronucleus formation and replicative index [21], but typically only at the highest concentrations and/or doses tested exceeding renal transport mechanisms or observed effects were transient. In addition, several studies have demonstrated the ability of 2,4-D to interrupt cellular functions and communication [22, 23], suggesting a potential nongenotoxic mode of action. Chlorophenoxy compounds are known to induce P450 [24] and to effectively bind to plasma proteins. Both 2,4-D and MCPA have been shown in studies ranging from rats to dogs to humans to be largely excreted as parent compounds and to a lesser extent as conjugates via urine within hours of exposure [25, 26]. There is general agreement that chlorophenoxy compounds do not accumulate in tissues. 

There have been numerous previous reviews evaluating the evidence for potential health effects associated with exposures to 2,4-D in particular as summarized in Table 1. The US EPA, US EPA Science Advisory Board, IARC, WHO, and Canadian government independently have conducted assessments of the carcinogenicity of 2,4-D, MCPA, and/or chlorophenoxy compounds generally [5, 9, 34, 45–48]. In 1991, the Center for Risk Analysis at the Harvard School of Public Health convened a panel of 13 scientists to weigh the evidence on the human carcinogenicity of 2,4-D [37]. The panel based its findings on a review of the toxicological and epidemiologic literature up to that time on 2,4-D and related phenoxy herbicides. The panel concluded that the toxicological data alone do not provide a strong basis for determination of carcinogenicity of 2,4-D. However, although they were unable to establish a cause-effect relationship, the panel concluded there was suggestive although inconclusive evidence for an association between exposure to 2,4-D and NHL and that further study was warranted. The panel further concluded there was little evidence of an association between 2,4-D use and soft-tissue sarcoma or Hodgkins disease and no evidence of an association between 2,4-D use and any other form of cancer. 

Focusing specifically on the epidemiologic studies, Johnson [36] conducted a review of the association between exposure to chlorophenoxy compounds and NHL, STS, HD, and other malignant lymphomas based solely on occupational cohort studies, and determined the weight of evidence at that time did not unequivocally support an association between use of chlorophenoxys and malignant lymphomas and/or STS, and that the available occupational cohort studies had not yet accumulated sufficient person years of observation to date. Nonetheless, despite the lack of sufficient person-years of observation, cases of lymphoma, and STS were observed when none were expected, suggestive of a potential association.

Munroe et al. [38] published a “comprehensive, integrated review and evaluation of the scientific evidence relating to the safety of the herbicide 2,4-D” and found no evidence for adverse effects across a range of outcomes. Focusing specifically on cancer, the authors found that only the case-control studies provided any evidence of an association between exposure to 2,4-D and NHL, specifically, and that this association was not borne out by the cohort studies. Finally, in an evaluation of the *in vitro* and *in vivo* data, they found no support for a mechanistic basis by which 2,4-D might lead to NHL.

Also in 1992, Morrison et al. [14] conducted a review of the literature and determined there was reasonable evidence suggesting that occupational exposure to phenoxy herbicides resulted in increased risk of developing NHL. The authors noted several studies showing large increases in risk of STS with phenoxy herbicide exposure but acknowledged that other studies had failed to observe increased risks and evidence for an exposure-risk relationship was lacking. A number of the underlying studies, particularly those showing elevated risks, included exposures to other constituents, such as dioxins.

In 1993, Bond and Rossbacher [39] published a review of potential human carcinogenicity of the chlorophenoxy herbicides MCPA, 2-(2-methyl-4-chlorophenoxy)propanoic acid (MCPP), and 2-(2,4-dichlorophenoxy)propionic acid (2,4-DP). They evaluated the epidemiologic evidence, particularly based on European studies, for associations between exposure to chlorophenoxy herbicides and cancer, including NHL, HD, and STS. The authors concluded that although suggestive evidence from epidemiologic studies of associations between chlorophenoxy herbicides and increased risks for several uncommon cancers existed, the evidence was inconsistent and far from conclusive. Further, none of the evidence specifically implicated MCPA, MCPP, or 2,4-D. Furthermore, the results of experimental studies in laboratory animals did not support a causal association between exposure these three compounds and cancer development. Similarly, Gandhi et al. [41] developed a critical evaluation of cancer risk from 2,4-D and found that there was no evidence for carcinogenicity of 2,4-D, although there was some suggestive evidence for NHL as an outcome, but without a plausible mode of action.

In 2002, Garabrant and Philbert [42] reviewed the scientific evidence from studies in both humans and animals relevant to cancer risks, neurologic disease, reproductive risks, and immunotoxicity of 2,4-D and its salts and esters focusing particularly on studies conducted from 1995 through 2001. The authors concluded that the available evidence from epidemiologic studies did not indicate any causal association of any form of cancer with 2,4-D exposure. Further, they found no human evidence of adverse reproductive outcomes related to 2,4-D. The available data from animal studies of acute, subchronic, and chronic exposure to 2,4-D and its salts and esters showed an unequivocal lack of systemic toxicity at doses that did not exceed renal clearance mechanisms. They found no evidence that 2,4-D in any of its forms activated or altered the immune system in animals at any dose. At doses exceeding or approaching renal clearance mechanisms, approximately 50 mg/kg in rats [49] 2,4-D was observed to cause liver and kidney damage and irritated mucous membranes. Although myotonia and alterations in gait and behavioral indices were observed following doses of 2,4-D that again exceeded renal clearance mechanisms, alterations in the neurologic system of experimental animals were not observed with the administration of doses in the microgram/kg/day range. The authors found it unlikely that 2,4-D exhibited any potential health effects at doses below those required to induce systemic toxicity. 

Bus and Hammond [43] summarized the findings of animal and human health studies primarily conducted or sponsored by the Industry Task Force II on 2,4-D Research Data (2,4-D Task Force) using the three forms of 2,4-D (acid, dimethylamine salts, and 2-ethylhexyl ester) and reported that chronic and other toxicity responses were generally limited to high doses, well above those known to result in nonlinear pharmacokinetic behavior. They further reported that 2,4-D did not demonstrate carcinogenicity or genotoxicity in animals, did not cause birth defects, and demonstrated low potential for reproductive toxicity and neurotoxicity, based on the additional studies provided to the US EPA in support of 2,4-D reregistration.

Despite these numerous reviews and several regulatory evaluations, questions as to the carcinogenic potential of 2,4-D and related compounds persist, prompting this paper.

### 1.1. Integrated Evaluation

There are a number of proposed approaches for evaluating the weight of evidence for a causal association between a particular exposure and a set of outcomes all of which rely to some extent on the use of Hill's criteria as applied to the body of evidence [50]. But defining criteria weights and the specific details of how the criteria are applied requires a clear definition of what constitutes “weight” and what constitutes “evidence” and how those components relate to each other without appearing *ad hoc* or purely the result of professional judgment. Ideally, one could use decision analytic techniques in which each individual study would receive a quantitative score across each of the clearly defined criteria resulting in an “objective” evaluation, but this becomes somewhat intractable, particularly in this case, given the large number of studies, the categories of studies (e.g., epidemiologic, *in vivo* and *in vitro*, and exposure), and the nuanced details of each study. 

Hypothesis-based weight-of-evidence [51, 52] provides a useful framework for evaluating hypotheses related to potential modes of action of chemical toxicity. Mode of action has regulatory significance with respect to the model used to develop toxicity factors and dose-response relationships for use in risk assessments [53], particularly with respect to carcinogenic outcomes, but most frameworks start with the premise that there is tumor induction observed in animal studies [54], which is not the case for 2,4-D or MCPA. Therefore, under the assumption that the epidemiologic studies are suggestive of an association between exposure to 2,4-D and/or MCPA and certain lymphohematopoietic outcomes, there is a benefit to evaluate those lymphohematopoietic outcomes with respect to disease etiology to identify key cellular events involved in either disease initiation or promotion to determine how those might relate to a potential mode of action for chlorophenoxy compounds to exert their biological influence. This strawman approach provides a framework for evaluating how much of the burden of disease might be attributable to environmental factors, specifically exposure to chlorophenoxy compounds. Since 2001, the International Lymphoma Epidemiology Consortium has dedicated itself to provide an open scientific forum and collaborative platform across which to pool data and conduct analyses related to lymphomas, particularly NHL. These investigators have made significant progress in identifying molecular pathways and events leading to subclinical progression of disease [55] that are explored here in the context of chlorophenoxy exposures. Is there evidence for a relationship between exposure and development of molecular events required for disease progression? And if so, how would exposure to chlorophenoxy compounds in the environment contribute to those events? And finally, are exposure concentrations sufficient to plausibly contribute to disease incidence? What is the evidence for population-level exposures, and how do those relate to concentrations at which effects have been observed across the different categories of studies (e.g., *in vivo* and *in vitro* toxicological, epidemiologic)?

**Figure 2:**
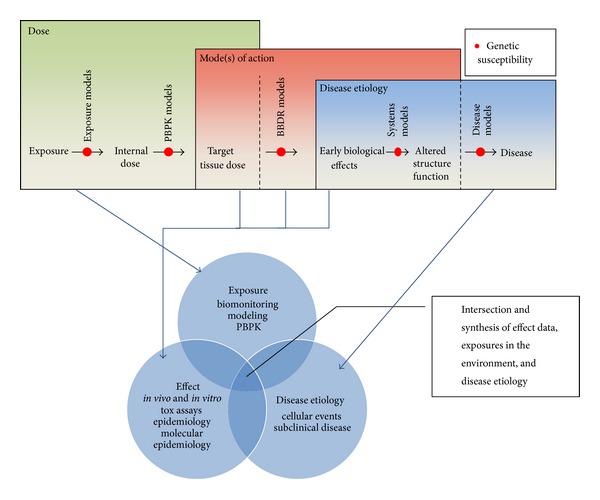
Intersection of data required to evaluate the plausibility of an association between exposure and outcome.

Figure 2 shows that synthesizing this information to determine the potential for exposure to chlorophenoxy compounds at environmentally relevant concentrations to lead to specific carcinogenic outcomes requires a critical evaluation of the intersection of environmental exposures (what are the exposure concentrations in the environment and how do those relate to biologically effective doses), the evidence for particular effects from toxicological and epidemiological data, and what is known about cellular events at the subclinical scale in terms of disease etiology. This allows an evaluation of biological plausibility with respect to a hypothesized mode of action based on the best available understanding of molecular events required for disease progression, evaluated in the context of what is known about how these compounds exert their biological influence, and exposure conditions necessary to achieve absorbed doses relevant to the pathways of interest. 

The structure of the paper is as follows. First, the rationale for focusing on lymphohematopoietic cancers is provided in Section 2 by summarizing and evaluating the key epidemiologic studies that have demonstrated an association between some measure of exposure to 2,4-D, MCPA, and/or chlorophenoxy compounds generally and lymphohematopoietic cancers.  Section 3 takes a “top down” approach by evaluating what is known about disease etiology to develop hypotheses concerning potential modes of action by which exposure to 2,4-D and/or MCPA might lead to the particular health outcomes identified in Section 2.  Section 4 identifies, discusses, and interprets the literature and data with respect to the kinetics of absorption, distribution, metabolism, and elimination in laboratory studies in humans and animals (Section 4.1), followed by a subsection on pharmacodynamics.  Section 5 focuses on toxicological studies, both *in vivo* and *in vitro*, starting with animal studies (Section 5.1) and then available human studies (Section 5.2).  Section 6 discusses exposures in the environment based on the available biomonitoring data and modeling studies in the context of the hypothesized modes of action. This is followed by a synthesis of the evidence in Section 7. 

## 2. Epidemiologic Studies

This section identifies the available epidemiologic studies and evaluates them with respect to a number of questions to identify the specific carcinogenic outcomes of interest. The first set of questions relates generally to study design, including the following.

- *How was exposure quantified?* A key limitation of epidemiological studies is related to the way in which exposures are quantified at best and categorized at worst. Many epidemiological studies rely on relatively crude measures of exposure such as basic occupational status (e.g., farmer, chlorophenoxy manufacturing) with years on the job as the primary measure of more or less exposure. Other studies make an attempt to quantify pounds of active constituent produced (for manufacturing facilities) or used (for sprayers, farmers, etc.). This information may or may not be combined with estimates of duration (e.g., two months a year for 12 years, etc.). Particularly for constituent usage, most estimates rely on questionnaires of various kinds, and in some cases, only next of kin is available to answer these questions. Studies that are able to use quantitative exposure information (e.g., biomarkers, etc.) allow for greater confidence in any observed associations.
- *What covariates were evaluated?* It is important to evaluate potential covariates of interest that might be related to disease (e.g., smoking) and certainly across the entire study population. This could include other potential exposures (e.g., solvents, other chemicals), and even if these arenot included directly in the evaluation, it is important to understand potential differences in exposures across the study population (e.g., cases have higher solvent exposures than controls, etc.). If the study is attempting to evaluate exposure across a number of constituents, then the statistical treatment needs to reflect these multiple comparisons to avoid spurious associations.
- *Is latency considered?* Most cancers require a series of events to occur over time following exposure; a study in which exposure and outcome are largely concurrent is less compelling than a study that has thought through the latency question. Weisenburger [100] suggests that the latency period for NHL, HD, leukemia for long term, and chronic exposures is on the order of 10–20 years as compared to short-term, high-intensity exposures for which the latency period is significantly shorter, on the order of five to six years.
- *How long was the follow-up period in cohort studies?* Related to latency but not exactly the same is the follow-up period in cohort studies. Again, particularly for chronic exposures and/or outcomes that are not expected for some time following exposure, it is important to allow enough follow-up time.
- *How were cases and controls selected in case-control studies?* Clearly, systematic differences across cases and controls will influence the analysis, particularly with respect to potential exposures.
- *How were outcomes identified and categorized?* In general, epidemiological studies rely on ICD classifications in use at the time of the study, but these change over time as our understanding of clinically relevant differences in disease become apparent. There is also the question of grouping outcomes with respect to mode of action. For example, within lymphohematopoietic outcomes, which include both lymphomas and leukemias, there are different cellular and molecular origins to disease relevant to the potential mode of action of an exposure such that it may not be appropriate to consider outcomes too broadly. That said, there may not be enough power to detect measurable differences across histological subtypes (e.g., follicular versus mantle cell lymphoma).

Another set of questions concerns the analysis and results, including the following.

- *What is the power of the study?* A challenge in epidemiological studies, particularly case-control studies with rare outcomes, is the power of the study to detect a relative risk of a certain magnitude.
- *Is there a dose-response relationship with measures of exposure (*e.g.,* job duration, years of use, etc.)?* In general, there is an expectation that higher and/or longer exposure would be associated with higher risk, depending on the potential mode of action of the compound.
- *What statistical tests are used?* As mentioned above, in the event of multiple exposures and comparisons, the statistical model used needs to account for that to avoid spurious associations.

Candidate studies were identified through a literature search, using PubMed, Medline, and Web of Science, for all epidemiologic studies related to 2,4-D, MCPA, and/or chlorophenoxy compounds and lymphohematopoietic cancers. Search terms included “lymph*” and “2,4-D,” or “MCPA,” “chlorophenoxy,” or “phenoxyacetic” and “human.” References for citations obtained this way were carefully reviewed to identify additional relevant studies. Papers were categorized as to type of study (e.g., case control, cohort) and general cohort (e.g., Swedish forestry workers, Finnish chlorophenoxy producers, US agricultural study, etc.). Results from the most recent, nonoverlapping analyses were the focus of this assessment.

**Figure 3:**
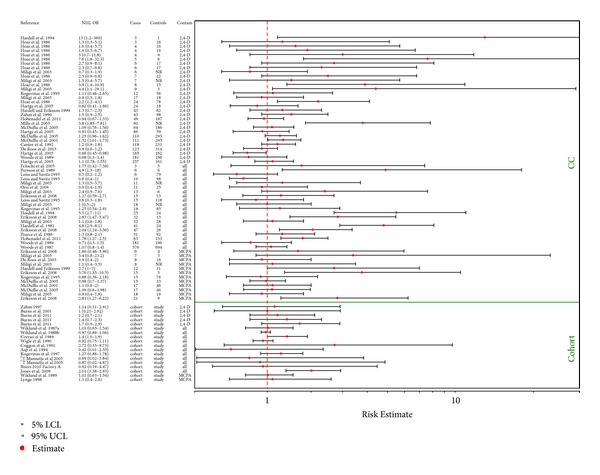
Summary of epidemiologic studies for NHL (see for review [12, 15–17, 56–89]).

**Figure 4:**
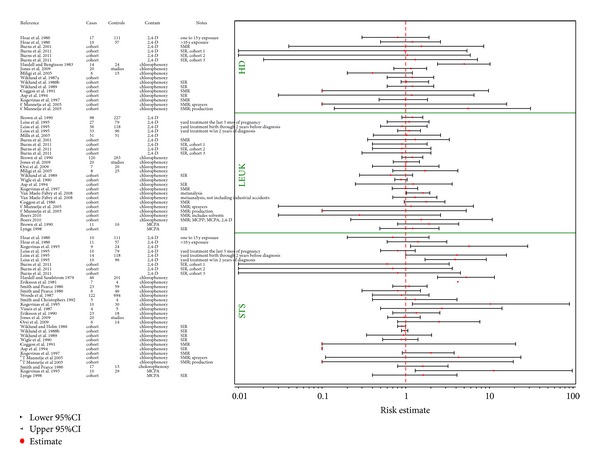
Summary of epidemiologic studies for STS, HD, and leukemia (see for review [12, 44, 57, 59, 66, 70, 75–80, 80–87, 89–99]).

Several studies in occupationally exposed case-control and to a lesser extent cohort studies show statistically significant associations (Figures 3 and 4) with a number of lymphohematopoietic outcomes, and these are the basis for concern with respect to potential health effects associated with exposures to 2,4-D and/or MCPA.  Figure 3 provides an overview of the epidemiologic studies related to NHL as an outcome, while results for the remaining cancers are graphically depicted in Figure 4.

### 2.1. Case-Control Studies

#### 2.1.1. NHL

Table 2 provides a summary of available case-control studies that have evaluated NHL as an outcome.  Figure 3 provides these results in a graphical format.

The strongest association between exposure to chlorophenoxy compounds and NHL is demonstrated through a series of occupational case-control studies carried out in Sweden [56, 60, 68, 71, 72, 90, 93, 94, 101–103]. These occupational studies focused on individuals involved in manufacturing chlorophenoxy compounds or professional sprayers, particularly in the forestry and railroad industries (e.g., spraying noxious weeds to maintain rights-of-way, etc.). The primary criticisms of these studies [104] include possible inaccurate diagnoses, observation and/or recall bias, lack of control for confounding variables, and poorly specified exposures (exposure is typically defined as greater than one day). Consequently, it is difficult to infer causality from these studies since there were numerous, largely statistically uncontrolled confounding exposures, and exposure itself was poorly specified, relying largely on self-reported questionnaires, and often without demonstrating dose-response relationships. For deceased cases, exposure categorization relied on next of kin, which may be particularly unreliable. Exposure was defined as greater than *one day* over many years.

These studies do not consistently demonstrate statistical significance or strength of association. For example, Hardell and Eriksson [60] conducted an analysis of a population-based case-control study in northern and middle Sweden with 404 NHL cases, 741 controls overall, and 12 NHL cases with 11 controls for the MCPA-specific analyses. They used questionnaires supplemented by telephone interviews to estimate exposure. They found a marginally statistically significant odds ratio for exposure to MCPA, but only when a latency period greater than 30 years was assumed. For other time periods, the association was not statistically significant. The odds ratio was less than 1.0 for exposure within 10–20 years of NHL onset, indicating reduced risk. Only the univariate analyses showed an increased OR = 2.7 (95% CI 1.0–7.0); the multivariate analysis OR = 1.2 (95% CI = 0.6–2.0). That is, only when exposures were individually modeled the authors did demonstrate statistical significance.

Another set of studies from the United States show more equivocal results. Hoar et al. [57] conducted a population-based, case-control study in Kansas based on telephone interviews with 200 white men diagnosed with NHL along with 1,005 controls. Use of chlorophenoxy herbicides (predominantly 2,4-D) in 24 cases and 78 controls was associated with an OR = 2.2 (95% CI = 1.2–4.1). Table 2 shows the results stratified by days per year use of 2,4-D, which shows that only the highest exposure was statistically significant, and the lowest exposure predicts a higher OR than the next two higher exposures. However, the number of cases and controls was very small when stratifying results. Zahm et al. [61] followed up with a population-based, case-control study in 66 counties in eastern Nebraska. Telephone interviews were conducted with 201 white men diagnosed with NHL between July 1, 1983 and June 30, 1986 with 775 controls. The authors report a 50% increase in NHL among men who mixed or applied 2,4-D (OR = 1.5, 95% CI 0.9–2.5). Reported ORs were largely unchanged when controlling for use of other pesticides and use of protective equipment. In fact, those farmers who reported typically using protective equipment had a higher OR (1.7, 95% CI = 0.9–3.1) as compared to those who did not (OR = 1.2, 95% CI = 0.6–2.4). It does not appear that the study controlled for smoking and/or other lifestyle factors. One issue to note with these studies is that the questionnaire used to determine exposure asked only about herbicide usage generally and therefore may not apply specifically to 2,4-D. Statistically significant associations were also found with triazines (OR = 2.5, 95% CI = 1.2–5.4), trifluralin (OR = 12.5, 95% CI = 1.6–116.1), and herbicides not otherwise named (OR = 5.8, 95% CI = 1.9–17.2).

Kogevinas et al. [59] report on a large, international, nested case-control study sponsored by the International Agency for Research on Cancer (IARC). Kogevinas et al. [59] evaluated 11 soft-tissue sarcoma and 32 lymphoma cases occurring within an international cohort which were matched for age, sex, and country of residence with 55 and 158 controls, respectively. Three industrial hygienists who were blind to case-control status estimated exposures to 21 chemicals or mixtures. In this study, the results for NHL were not statistically significant and showed ORs less than one (Table 2). Predicted ORs did not show a dose-response relationship across exposures when expressed as referent, low, medium, and high (e.g., the lowest exposure, in some cases, had the highest predicted OR, but sample sizes were very small when defined this way). A strength of this study is the international scope, with cases and controls based on worldwide cohorts.

Other studies, particularly those that evaluated multiple exposures and/or dose-response relationships, do not demonstrate a convincing relationship between exposure and outcome. For example, Cantor et al. [64] report on a case (*n* = 622) control (*n* = 1245 population-based) study which evaluated potential exposures across a wide range of pesticides, herbicides, and insecticides and found statistically significant positive associations with exposure to malathion, DDT, chlordane, and lindane, but not chlorophenoxys (largely 2,4-D) and NHL. Similarly, McDuffie et al. [13] conducted a Canadian multicenter population-based incident, case (*n* = 517) control (*n* = 1506) study among men in a diversity of occupations using an initial postal questionnaire followed by a telephone interview for those reporting pesticide exposure of 10 h/year or more and a 15% random sample of the remainder. Adjusted odds ratios (ORs) were computed using conditional logistic regression stratified by the matching variables of age and province of residence, and subsequently adjusted for statistically significant medical variables (history of measles, mumps, cancer, allergy desensitization treatment, and a positive history of cancer in first-degree relatives). They found that among major chemical classes of herbicides, the risk of NHL was statistically significantly increased for exposure to phenoxy herbicides (OR = 1.38; 95% CI = 1.06–1.81), although a detailed evaluation of individual phenoxy herbicides found the highest individual OR for mecoprop (OR = 2.33; 95% CI = 1.58–3.44) rather than 2,4-D or MCPA. Moreover, across the entire study, the highest ORs were found for aldrin (OR = 4.18; 95% CI = 1.48–11.96). In their final models, NHL was most highly associated with a personal history of cancer; a history of cancer in first-degree relatives; exposure to dicamba-containing herbicides, to mecoprop, and to aldrin. Their final models did not include 2,4-D or MCPA.

Miligi et al. [58] conducted a population-based case-control study in Italy based on 1,575 interviewed cases and 1,232 controls in the nine agricultural study areas. Exposure to nitroderivatives and phenylimines among fungicides, hydrocarbon derivatives and insecticide oils among insecticides, and the herbicide amides were the chemical classes observed to be associated with developing NHL. ORs for the chlorophenoxy compounds are presented in Table 2 and are slightly elevated in some cases but all statistically insignificant. Exposure was assigned as a probability of usage in terms of chemicals families and active ingredients according to an ordinal scale (low, medium, and high) taking into account the time period, crops and crop diseases, and treatment applied as well as the area. Industrial hygienists reviewed questionnaire data on crop diseases, treatments carried out and historical periods, field acreage, geographical location, and self-reported use of specific pesticides. The agronomists involved in the pesticide exposure assessment based their judgments on personal local experience, national statistics on pesticide use per year and administrative unit, available records of local pesticide suppliers, records of pesticide purchases by the major farms, and on professional consultants for the different crops. Miligi et al. [58] report on several additional analyses that find a statistically significant OR = 4.4 (95% CI = 1.1–29.1) based on 9 cases and 3 controls related to 2,4-D usage without protective equipment. The wide confidence interval (e.g., small *n*) makes it difficult to infer a relationship.

In an “integrative” study evaluating many potential pesticides and combinations of pesticides, De Roos et al. [16] report on a pooled analysis from three case-control studies conducted under the auspices of the National Cancer Institute in the United States based on data from the 1980s. The authors used these pooled data to examine pesticide exposures in farming as risk factors for NHL in men. The large sample size (*n* = 3417) allowed analysis of 47 pesticides simultaneously, controlling for potential confounding by other pesticides in the model, and adjusting the estimates based on a prespecified variance to make them more stable. Reported use of several individual pesticides was associated with increased NHL incidence, including organophosphate insecticides coumaphos, diazinon, fonofos, insecticides chlordane, dieldrin, copper acetoarsenite, herbicides atrazine, glyphosate, and sodium chlorate. A subanalysis of these “potentially carcinogenic” pesticides suggested a positive trend of risk with exposure to increasing numbers. Estimated ORs for 2,4-D and MCPA were both below one (Table 2) and were not elevated nor significant in any combined model.

However, Mills et al. [12] in a study involving 131 lymphohematopeoitic cancers diagnosed in California between 1988 and 2001 in United Farm Workers of America (UFW) members found a statistically significant OR = 3.8 (95% CI = 1.85–7.81) for exposure to 2,4-D. This was the only statistically significant association for NHL across all pesticides studied. However, while the authors included age, sex, and length of union affiliation as covariates, there was no mention of controlling for smoking and/or other risk factors that may be associated with NHL. Exposure was characterized by linking UFW job histories (records kept by the union) to records of pesticide use by county kept by the State of California Pesticide Databank. Employment in a given crop in a given month/year in a given county was matched to the corresponding application of several pesticides on that crop in a given month and county location. These applications (in pounds of active ingredients applied) were summed and used as a proxy or surrogate measure of pesticide exposure for both cases and controls for the two- to three-decade period prior to diagnosis of the cancer. However, although exposure was better characterized than in most epidemiologic studies, there was no verification of any individual exposure (e.g., true individual exposures were completely unknown).

Orsi et al. [70] conducted a hospital-based case-control study in six centers in France between 2000 and 2004. The cases were incident cases with a diagnosis of lymphoma aged 18–75 years. During the same period, controls of the same age and sex as the cases were recruited in the same hospital, mainly in the orthopaedic and rheumatological departments. Exposures to pesticides were evaluated through specific interviews and case-by-case expert reviews. The authors calculated ORs and 95% CIs using unconditional logistic regressions and did not find an increased OR for occupational exposure to chlorophenoxy compounds as a class and NHL. Hohenadel et al. [17] report on the results of the cross-canada study of pesticides and health, a case-control study of Canadian men 19 years of age or older, conducted between 1991 and 1994 in six Canadian provinces (Alberta, British Columbia, Manitoba, Ontario, Quebec, and Saskatchewan). A combination of postal and telephone interviews were used to obtain data for covariates and for pesticide use. Stratifying respondents based on use of 2 or more chlorophenoxy herbicides showed a statistically significant OR = 1.78 (95% CI = 1.27–2.5). However, a model with only exposure to 2,4-D resulted in an OR <1, and in a larger evaluation of combinations of pesticides, malathion consistently emerged as a statistically significant exposure while 2,4-D did not.

All the previous studies have involved occupational exposures, which may not be particularly relevant to residential settings or the general public with respect to actual exposure levels in the population and potential risks associated with a significant use of chlorophenoxy compounds. One study, however, Hartge et al. [15] explored the relationship between residential use of herbicides (primarily on lawns) and NHL in a population case-control study across Iowa, metropolitan Detroit, Los Angeles, and Seattle from the period 1998 to 2000. The authors calculated relative risks based on measured 2,4-D in carpet dust (Table 2) as well as self-reported herbicide (specific chemicals not provided) use on lawns. The authors did not observe a relationship between estimates of exposure and NHL. 

Another study, Leiss and Savitz [69] explored associations between home pesticide use and childhood cancers in a study that grew out of childhood cancer and electromagnetic field exposure in Colorado. Exposure data was collected through parental interviews and dichotomized as “any use” versus “no use” for each pesticide type and exposure period based on the question whether the yard around the residence was “ever treated with insecticides or herbicides to control insects or weeds.” No associations with lymphomas, broadly defined, were found with all predicted ORs less than one.

#### 2.1.2. STS

The bottom portion of Figure 4 provides a summary of the available STS studies.

Hardell and Sandstrom [93] estimated an OR of 5.3 (95% CI = 2.4–11.5) for STS based 13 cases and 14 controls from the same Swedish case-control study as described previously for NHL. A follow-on study by Eriksson et al. [94] estimated an elevated although nonstatistically significant OR of 4.2 for chlorophenoxy exposures free from TCDD contamination (e.g., MCPA, 2,4-D, mecoprop, and dichlorprop). The New Zealand studies [98, 105] estimated a slightly elevated although nonstatistically significant OR in one study ([105], OR = 1.6; 95% CI = 0.8–3.2; 17 cases and 13 controls) and an OR less than one in another (Smith and Pearce [98]; OR = 0.7; 95% CI = 0.3–1.5; 6 cases and 46 controls). 

Woods et al. [66] in a study in western Washington state estimated an OR = 0.8 (95% CI = 0.5–1.2) assuming predominantly chlorphenoxy exposures, and Cantor et al. [64] estimated a nonsignificant OR = 1.2 (95% CI = 0.9–1.6) based on 118 cases and 231 controls. Vineis et al. 1986 [106] in a study in Italy involving female rice weeders found a nonsignificant OR = 2.7 (95% CI = 0.59–12.37) based on 31 cases and 73 controls. Exposure to chlorophenoxy compounds (likely including 2,4,5-T) was based on three categories: no exposure, maybe exposed, and definitely exposed. Rice weeders were considered exposed to phenoxy herbicides when they worked after 1950 and did not work exclusively in a small rice allotment of their own. The “maybe” category was used particularly for people engaged in corn, wheat, and pasture growing after 1950.

Hoar et al. [57] conducted a population-based, case-control study in Kansas based on telephone interviews with 200 white men diagnosed with STS along with 1,005 controls. Estimated ORs were all below one except for 11 cases (57 controls) with greater than 16 years of exposure (OR = 1.4; 95% CI = 0.6–3.1).

The strongest associations between chlorophenoxy compound exposure and STS were found by Kogevinas et al. [59] based on 10 cases and 30 controls, who estimated an OR = 10.3 (95% CI = 1.2–90.6). When stratifying results by predominantly MCPA/MCPP exposures, the estimated OR increased to 11.27 (95% CI = 1.3–97.9) based on 10 cases and 29 controls and decreased to 5.72 but still statistically significant (95% CI = 1.14–28.7) based on 9 cases and 24 controls for exposures identified as predominantly 2,4-D related. Exposures to 21 chemicals or mixtures were estimated by three industrial hygienists who were blind to the subject's case-control status, but a dichotomous exposure classification was applied which likely included considerable misclassification (according to the authors page 398) since information on dates and quantities of production and spraying of the six pesticides was not consistently available. Results are presented for four exposure categories (none, low, medium, and high), and the predicted ORs for the chlorophenoxys and MCPA do not follow a dose-response relationship, while dose-response relationships were observed for TCDD, 2,4-D, and 3,4-5-T. However, results presented in this way were not statistically significant except for the highest predicted exposure for chlorophenoxys generally. The authors used a logistic model and developed results for each contaminant individually. 

Another study, Leiss and Savitz [69] explored associations between home pesticide use and childhood cancers in a study that study that grew out of childhood cancer and electromagnetic field exposure in Colorado. Exposure data was collected through parental interviews, and dichotomized as “any use” versus “no use” for each pesticide type and exposure period based on the question whether the yard around the residence was “ever treated with insecticides or herbicides to control insects or weeds.” Separate ORs were estimated for exposure during the last three months of pregnancy (OR = 0.8; 95% CI = 0.5–1.3 based on 10 cases and 79 controls), exposure between birth and two years of diagnosis (OR = 4.1; 95% CI = 1.0–16.0), and exposure between two years of diagnosis and diagnosis (OR = 3.9; 95% CI = 1.7–9.2). 

The cohort studies do not show an association between exposure and STS as an outcome. Only two of the case control studies show statistically significantly increased ORs [59, 69]. Leiss and Savitz [69] focused on childhood STS, and exposure was only specified as “yard treatment.” 

#### 2.1.3. HD

The top portion of Figure 4 provides the results of the epidemiologic studies focusing on HD.

The Swedish studies [68, 90] show mixed results for HD as an endpoint. Persson et al. [68] estimated an OR = 3.8 (95% CI = 0.7–21) based on 4 cases and 6 controls for HD cases with exposure to predominantly chlorophenoxy compounds broadly defined. The only statistically significantly increased OR = 5.0 (95% CI = 2.4–10.2) based on 14 cases and 24 controls [90] was for a study for which exposure was categorized as at least one day of exposure to chlorophenoxy compounds based on a self-administered questionnaire. A latency period of at least five years was assumed by excluding all exposures within five years of diagnosis. 

Hoar et al. [57] conducted a population-based, case-control study in Kansas based on telephone interviews with 173 white men diagnosed with HD with 1,007 controls. None of the estimated ORs for HD was statistically significant, and was only greater than one for greater than 16 years of exposure (OR = 1.2; 95% CI = 0.5, 2.6). 

Finally, Orsi et al. [70] found a nonsignificant OR = 2.5 (95% CI = 0.8–7.7) based on 6 cases and 14 controls for occupational exposure of agricultural workers to chlorophenoxy compounds as a class for HD. This hospital-based case-control study obtained all cases of lymphoid neoplasms from the main hospitals of the French cities of Brest, Caen, Nantes, Lille, Toulouse, and Bordeaux between September 2000 and December 2004. Exposure was categorized first through a self-administered questionnaire and followed up by 90 minute individual face-to-face interviews.

#### 2.1.4. Leukemia

The middle portion of Figure 4 provides the results of the studies investigating leukemia as an endpoint. 

To investigate whether exposure to carcinogens in an agricultural setting is related to an increased risk of developing leukemia, Brown et al. [91] conducted a population-based case-control interview study of 578 white men with leukemia and 1245 controls living in Iowa and Minnesota. They found a slight, but significant, elevation in risk for all leukemia (OR = 1.2) and chronic lymphocytic leukemia (OR = 1.4) for farmers compared to nonfarmers, but there were no significant associations with leukemia for exposure to specific herbicides (including 2,4-D and 2,4,5-T). However, significantly elevated risks for leukemia of >2.0 were seen for exposure to specific animal insecticides including the organophosphates crotoxyphos (OR = 11.1), dichlorvos (OR = 2.0), famphur (OR = 2.2), and the natural product pyrethrins (OR = 3.7), and the chlorinated hydrocarbon methoxychlor (OR = 2.2). There were also smaller, but significant, risks associated with exposure to nicotine (OR = 1.6) and DDT (OR = 1.3). Based on exposure 2,4-D alone, Brown et al. [91] estimated an OR = 1.2 (95% CI = 0.9–1.6) based on 98 cases and 227 controls, and for MCPA, the estimated OR = 1.9 (95% CI = 0.8–4.3) based on 11 cases and 16 controls.

Leiss and Savitz [69] found no associations between 2,4-D use and leukemia with all predicted ORs less than one.

Orsi et al. [70] did not find an increased OR when evaluating leukemia broadly, but disaggregated by subtype, found an increased OR = 4.1 (95% CI = 1.1–15) for hairy cell leukemia, specifically, based on 4 cases and 20 controls. The overall OR = 1.0 (95% CI = 0.4–2.5) based on 7 cases and 20 controls largely exclusively exposed to chlorophenoxy compounds. This hospital-based case-control study obtained all cases of lymphoid neoplasms from the main hospitals of the French cities of Brest, Caen, Nantes, Lille, Toulouse, and Bordeaux between September 2000 and December 2004. Exposure was categorized first through a self-administered questionnaire and followed up by 90 minute individual face-to-face interviews.

Van Maele-Fabry et al. [44] conducted a meta-analysis focused on three cohort studies published between 1984 and 2004 found a statistically significant odds ratio (OR) for exposure to chlorophenoxy compounds and leukemia (OR = 1.60, 95; confidence interval (CI) = 1.02–2.52), although all three underlying studies individually showed nonsignificant associations ([86, 92, 113]—factory B only). 

Agricultural risk factors for lymphohematopeoitic cancers, including leukemia, in Hispanic farm workers in California were examined in a nested case-control study embedded in a cohort of 139,000 ever members of a farm worker labor union in California [12]. Risk of leukemia was associated with exposure to the pesticides mancozeb (OR = 2.35; 95% I = 1.12–4.95) and toxaphene (OR = 2.20; 95% CI = 1.04–4.65) but not 2,4-D (OR = 1.03; 95% CI = 0.41–2.61).

### 2.2. Cohort Studies

The cohort studies, Table 3, by their design, evaluate all cancers simultaneously rather than focusing on particular cancers as is often found in the case-control studies. Table 3 presents the results of the major cohort studies and in general show few statistically significant associations except for two [89, 111]. The Carrao et al. [111] cohort consisted of 25,945 male farmers licensed between 1970 and 1974 to buy and use pesticides without any further refinement of what pesticides were used, how often, and in what quantities. They estimated a standardized incidence ratio (SIR) of 1.4 (95% CI = 1.0–1.9) across the category “all malignant lymphomas” (which considers all the lymphohematopoietic cancers as a single category) and conclude that this is likely due to exposure to chlorophenoxy compounds. The rationale for this is that first, because the higher incidence was only found in predominantly arable areas, where, the authors argue, greater use is made of herbicides (although the specific herbicides in use are not discussed, and the assumption is that these herbicides are largely chlorophenoxys with no justification), and second, the authors argue that the use of chlorophenoxy acid products had increased in recent years, so much so that this must represent the predominant exposure. It is therefore difficult to argue that this analysis shows much support for a relationship between exposure to chlorophenoxy compounds and lymphoma.

The Jones et al. [89] study is a systematic review and meta-analysis of studies of cohorts of workers in the crop protection product manufacturing industry. Jones et al. estimated meta SMRs based on 20 individual studies and found a statistically significantly increased SMR for lymphoma, broadly defined, and exposure to chlorophenoxys (SMR = 2.01; 95% CI = 1.38–2.93) [89]. Although the SMR for HD was greater than one, it was not statistically significant. A limitation of this meta-analysis is that the underlying studies included all chlorophenoxy compounds, including 2,4,5-T, which, as acknowledged by the authors, is likely to have been contaminated with dioxin.

There have been a series of studies exploring cancer mortality and/or incidence rates in a cohort of 2,4-D manufacturing workers from the Dow Chemical Company in Midland, MI, USA [75, 79, 110, 112]. In the first of these, Bond et al. [110] estimated standardized mortality ratios (SMRs) for 878 chemical workers potentially exposed to 2,4-D at anytime between 1945 and 1983. Observed mortality was compared with expected levels based on adjusted rates for United States white men and for other male employees from a manufacturing location who were not exposed to 2,4-D. Analyses by production area, duration of exposure, and cumulative dose showed no patterns suggestive of a causal association between 2,4-D exposure and any other particular cause of death. Similarly, follow-up studies have not provided evidence that exposures in manufacturing workers have led to increased risks.

Wiklund et al. [78] report on a cohort consisting of 20,245 subjects (99% men; 1% women) who had a license for pesticide application issued between 1965 and 1976 in Sweden. Approximately 20% of subjects reporting using herbicides during the 1950s: 51% for the 1960s and 68% for the 1970s. The most commonly used herbicide across all three decades was MCPA. The authors found a decreased relative risk across all cancers.

Bond and Rossbacher [39] report on two studies based on cohorts that manufactured MCPA. The first, Lynge [107] evaluated 4459 chemical workers from two of four companies that had produced phenoxy herbicides in Denmark, although these workers were also engaged in the manufacture of diverse chemical products including not only herbicides but dyes and pigments as well. Roughly one third of them (*n* = 940) had been assigned to phenoxy herbicide production or packaging. MCPA and MCPP were the predominant phenoxy herbicides produced, followed by 2,4-D and 2,4-DP. Five cases of soft-tissue sarcoma were reported among the men as compared to expected (relative risk = 2.7; (95% confidence interval) 0.88–6.34) and no cases among the women. A slight deficit of total cancer was noted among the combined group of chemical workers. The second, Coggon et al. [92] examined mortality and cancer incidence in 5784 men who had been employed in manufacturing or spraying MCPA in the United Kingdom. Workers were classified according to their potential for exposure into high, low background based on their job titles. Overall mortality in the cohort was less than that expected from national death rates, as was mortality from all neoplasms, heart disease, and diseases of the respiratory system. Only one death from soft-tissue sarcoma occurred compared with one expected. Three men died from malignant lymphoma compared with nine expected.

Lynge [86] conducted a follow-up cohort study of 2119 workers from Denmark employed at two factories that produced phenoxy herbicides since 1947 and 1951, respectively. From 1947 to 1993 the 2119 workers showed a slightly lower overall cancer incidence than the Danish population (observed = 204; expected = 234.23; SIR = 0.87; 95% CI = 0.8–1.0). Four soft-tissue sarcoma cases were observed (expected = 2.47; SIR = 1.62; 95% Cl = 0.4–4.1). There were six cases of NHL (expected = 5.07; SIR = 1.10; 95% CI = 0.4–2.6) and no significantly elevated risk of other cancers. A follow-up study by Coggon et al. [81] in 2239 men employed in the United Kingdom from 1963 to 1985 observed two deaths from NHL with 0.87 expected, a difference that was not statistically significant. No cases of STS or HD were recorded.

In a cohort study published in 2005, 'T Mannetje et al. [84] followed 813 phenoxy herbicide producers 699 sprayers from January 1, 1969 and January 1, 1973, respectively, until December 31, 2000. The authors calculated SMRs using national mortality rates and found a 24% nonsignificant excess cancer mortality in phenoxy herbicide producers, with a significant excess for multiple myeloma. Associations were stronger for those exposed to multiple agents including dioxin during production. Overall cancer mortality was not increased for producers and sprayers mainly handling final technical products. 

Burns et al. [79] conducted a cohort study of male employees of The Dow Chemical Company who manufactured or formulated 2,4-D anytime from 1945 to the end of 1994. Their mortality experience was compared with national rates and with more than 40,000 other company employees who worked at the same location. There were no significantly increased SMRs for any of the causes of death analyzed. When compared with the United States rates, the SMR for NHL was 1.00 (95% CI = 0.21–2.92).

Boers et al. [85] report on a third follow-up of a retrospective cohort study involving two chlorophenoxy herbicide manufacturing factories, producing mainly 2,4,5-T (factory A) and MCPA/MCPP (factory B) found no statistically significant increases in lymphohematopoietic cancer deaths, although SMRs were greater than one. 

Aside from agricultural and forestry uses of 2,4-D, the lawn care industry also uses 2,4-D. Zahm [74] conducted a retrospective cohort mortality study of 32,600 employees of a lawn care company and found four deaths due to NHL (SMR = 1.15, 95% CI = 0.31–2.91). Two of the (male) applicators had been employed longer than three years, and for those, the predicted SMR was 7.11 (95% CI = 1.78–28.42). Risks of NHL increased for male applicators, especially those employed for three or more years, but no quantitative or semiquantitative measures of pesticide use or exposure were presented.

### 2.3. Summary of Epidemiologic Studies

Associations between exposures to chlorophenoxy compounds (including 2,4-D and MCPA) and potential outcomes have generally been developed through occupational studies in manufacturing facility workers and/or agricultural workers. Many of the underlying studies suffer from poor exposure specification (e.g., not clear which phenoxy herbicides were actually used and/or manufactured, whether there was cross-contamination from dioxin or other constituents, and actual exposures and doses experienced by cases and/or cohorts); poor covariate control (e.g., smoking status); insufficient sample sizes; insufficient follow-up for the cohort studies. Nonetheless, the results of the reviews are equivocal, with some suggesting an association with NHL but others not, and most indicating that an association with STS, HD, and/or leukemia is weak at best given the generally observed lack of statistical significance and risk measures less than one. Those studies that included more realistic exposures (e.g., a variety of pesticides, etc.) tended to reduce the influence of 2,4-D and/or MCPA than those studies considering only chlorophenoxy exposure alone. The few studies available for exposures likely to be experienced by the general public and/or residential use of 2,4-D and MCPA found no associations with health outcomes. 

The way in which diseases are grouped and categorized plays an important role in epidemiological studies. Sorting results by histological subtype can lead to small numbers and reduced power, increasing the probability of finding a particular association simply by chance. However, there may be important differences with respect to exposure in terms of disease outcome (e.g., exposure to a particular causal agent leads to only one histological subtype). Many different types of groupings have been used in analyzing epidemiologic data, primarily reflecting the classification system in use at the time of diagnosis or cause of death, and a confounding factor is that these classifications change over time. Our understanding of disease etiology is always growing, and increasingly researchers are able to identify key molecular and cellular transformations required for disease progression. This introduces a challenge for epidemiologic studies in that it may not be appropriate to consider all histological subtypes of a particular carcinogenic outcome relative to a hypothesized exposure (and by extension, mode of action), or it may be possible to incorporate cellular changes into measures of exposure and/or effect in epidemiologic studies (discussed in the next section).  Table 4 provides a summary of the available epidemiologic studies that have evaluated potential exposures and NHL outcomes by subtype and shows that a consistent relationship between exposures and outcomes defined by histological subtypes does not emerge. 

In summary, the available epidemiologic studies are as follows.

- Show inconsistent relationships between exposure to chlorophenoxy compounds generally, 2,4-D and/or MCPA specifically, and lymphohematopoietic outcomes.
  - Strongest association is for NHL based on case-control studies.
  - No statistically significant associations for leukemia.
  - Some evidence for STS and HD but with numerous confounding exposures, exposures poorly specified, and small sample sizes.
  - Cohort studies show no statistically significant associations across studies save one.
- The strongest association appears to be between exposure to MCPA and NHL and only in the agricultural or forestry professions.
- Studies in lawn care professionals and in residential settings do not support an association between exposure and outcome.
- With few exceptions, there are no observable dose-response relationships across the studies.
- The crudest measures of exposures tend to show the strongest associations.
- Exposure characterization relies predominantly on self-reported questionnaires, in some cases with follow-up interviews. “Exposed” typically defined as greater than one day of exposure.
- Those studies that focused on single assumed exposures based on univariate analyses tended to show the highest associations (e.g., statistical significance is typically only achieved through univariate analyses).

## 3. Molecular Events in Disease Progression

This section focuses on the evidence for key events at the molecular level associated with an increased risk of developing lymphohematopeoitic cancers with a particular emphasis on NHL. 

**Figure 5:**
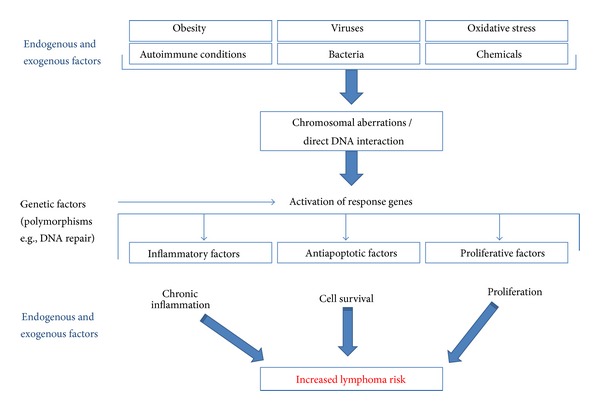
Schematic of general pathways leading to increased risk of developing lymphoma.

Lymphohematopoietic neoplasia is characterized by an uncontrolled proliferation or expansion of cells originating from the bone marrow or lymphoid tissues that do not retain the capacity to differentiate normally to form mature blood cells. In general, current evidence indicates the vast majority of leukemia-inducing agents are believed to act through a mutagenic mode of action, whereas the lymphoma-inducing agents are hypothesized to most likely act through immunomodulation and related effects including indirect DNA interaction [114]. The acute and chronic myeloid leukemias (CMLs), precursor lymphomas, acute lymphoblastic leukemias- (ALL-)B lymphoblastic leukemia/lymphoma, and T lymphoblastic leukemia/lymphoma originate in hematopoietic stem or progenitor cells while the majority of lymphomas (NHL, Hodgkin lymphoma, and Burkitt lymphoma), all myelomas, as well as several rare leukemias/lymphomas (adult T-cell leukemia, prolymphocytic leukemia, and hairy cell leukemia), and one common (CLL) leukemia originate in mature lymphoid cells [55, 114].  Figure 5 presents the generalized pathways by which the risk of developing lymphoma is increased. 

**Figure 6:**
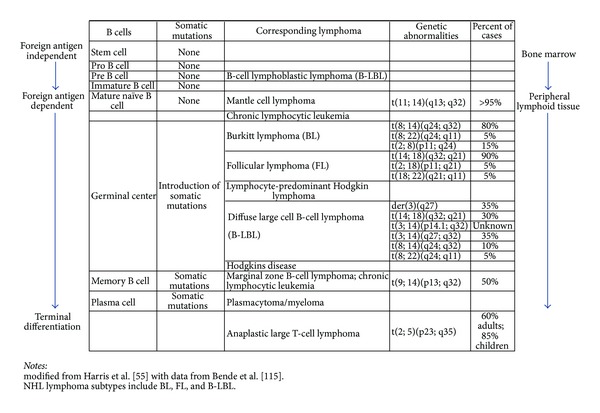
Key steps in the development of NHL.

Non-Hodgkin lymphomas, in particular, represent a heterogeneous group of diseases deriving from mature B cells (85% of cases) and, in a minority of cases, from T cells [55].  Figure 6 provides a schematic of the individual steps in the progression of B-cells from a stem cell to a final plasma cell. Most NHLs arise from the pre B cell and mature naïve B cell stages. This table also shows the most common, unique genetic changes that have been associated with particular forms of lymphoma, and the percent of cases in which these have been observed. 

In early and late stages of B-cell development, genetic polymorphisms, and environmental exposures influence the fate of a B cell and its chances of undergoing neoplastic transformation as shown in Figure 6. The majority of low-grade B-cell lymphomas (e.g., follicular lymphoma) originate in the germinal center. This stage of B-cell development combines extensive DNA modification with vigorous proliferation [115], thus this is a susceptible development point with respect to exogenous exposures. The body responds to such strand breaks and deletions by activating DNA repair genes, many of which are not present in polymorphic individuals, conferring potential susceptibility. Finally, following repair (or misrepair), there is another opportunity for endogenous and exogenous agents to interrupt key cellular functions by causing or exacerbating chronic inflammation, cell proliferation, and/or interfering with apoptosis. Although experimental models indicate that chromosomal translocations contribute to lymphoma and occur in virtually all lymphomas, there is a significant body of evidence indicating that these translocations alone are not sufficient to cause disease in the absence of promoting mechanisms, indicating a multistage process is required for complete disease to occur [55, 116]. 

### 3.1. Direct DNA Interaction and Repair

A key hypothesized event in lymphomagenesis is unrepaired and/or misrepaired DNA strand-breaks [55, 117], and specific associations with particular forms of NHL are shown in Figure 6. For example, one of the most common chromosomal abnormalities in NHL is the t(14;18)(q32;q21) translocation, which occurs in 70% to 90% of cases of follicular lymphoma, 20% to 30% of diffuse large B-cell lymphoma, and 5% to 10% of other less common subtypes [55, 117–119]. Under normal conditions, lymphocytes must strictly regulate growth and apoptosis to provide adequate immunologic defenses against infections while not overwhelming the organism with inappropriate cell numbers. The t(14;18) translocation joins the BCL-2 gene on chromosome 18 to the immunoglobulin heavy chain gene on chromosome 14, leading to an inhibition of apoptosis through Bcl-2 overexpression and, consequently, prolonged survival of the affected B cells. Evidence is growing that agricultural exposures are associated with significant t(14;18) translocations [118, 120, 121]. Recently, Agopian et al. [122] established a direct, molecular connection between agricultural pesticide use, t(14;18) in blood, and malignant progression, verifying that expanded t(14;18)+ clones truly represent malignant precursors for development of follicular lymphoma. 

However, Garry et al. [123] investigated the possible relationships between agricultural pesticide exposure and the increased risk of NHL among farm workers in the north central United States by performing G-banded chromosome analyses of peripheral blood from workers classified according to primary types of pesticide exposure: herbicides (*n* = 20), insecticides (*n* = 18), fumigants (*n* = 23), and occupationally unexposed controls (*n* = 33). The most commonly used herbicides in this study included eradicane (thiocarbamate) and 2,4-D, although all pesticide use was only qualitatively described. Increased lymphoma risk and excess breaks involving band 18q21 in herbicide appliers were observed. Given that 2,4-D was (qualitatively) the most common herbicide, a putative link was hypothesized. However, another study [124] found no correlation between measured urinary levels of 2,4-D and observed chromosomal aberrations in occupationally exposed forestry workers.

A study conducted by Schroeder et al. [125] used pesticide data derived from a population-based, case-control study conducted in Iowa and Minnesota between 1981 and 1983. The parent study included 622 cases and 1245 controls and was limited to men. Tumor blocks were retrieved for 248 of the 622 cases (40%) in the parent case-control study, and the presence of the t(14;18) translocation in tumor tissue was determined by polymerase chain reaction. One hundred eighty-two of the 248 blocks (73%) were successfully assayed, and 37% (68) of these cases were t(14;18)-positive, whereas 63% (114) were t(14;18) negative. Schroeder et al. [125] found that the t(14;18) positive NHL cases tended to have larger relative risks from agricultural exposures than t(14;18) negative cases. Report ORs for specific exposures: chlorophenoxy herbicide use (*n* = 266 controls; *n* = 17 t(14;18) positive cases; *n* = 30 t(14;18) negative cases) resulted in an OR = 0.9 (95% CI = 0.5–1.5) for the positive t(14;18) cases and OR = 1.1 (95% CI = 0.5–1.5) for the negative t(14;18) cases. For all statistically significant associations, the number of positive t(14;18) cases exceeded the number of negative t(14;18) cases (e.g., lindane, cyclodienes as a class, dieldrin, toxaphene, atrazine, phthalimide, and a fumigant). Estimated ORs were less than one for exposure to chlorophenoxy compounds. Similarly, Chiu et al. [126] found a consistent relationship with respect to dieldrin, lindane, and toxaphene exposures, but no relationship with chlorophenoxy compounds, suggesting that although the evidence is increasing that this particular chromosomal aberration is significant with respect to NHL etiology [122], there is little support for a causal role for chlorophenoxy compounds in general and specifically 2,4-D [124]. 

Genetic polymorphisms in DNA repair genes have also been shown to contribute to lymphomagenesis [117, 127]. As noted, DNA breaks and other types of DNA damage are strongly implicated in lymphoma development, and there are five overlapping DNA repair pathways that are typically invoked to repair such breaks: (1) nonhomologous end joining (NHEJ) genes, (2) homologous recombination (HR) repair, (3) nucleotide excision repair (NER), (4) base excision repair (BER), and (5) direct damage reversal. V(D)J recombination involves the deliberate introduction of double-strand breaks that reshuffle dozens of Ig building blocks, the V, D, and J segments. This process produces a highly diverse repertoire of antibodies, which are induced by a wide spectrum of antigenic challenges. Errors by the NHEJ genes responsible for ligating the V, D, and J segments are implicated at the sites of rearrangements characteristic of NHL. In addition, two steps that follow V(D)J in B-cell maturation, class-switch recombination, and somatic hypermutation also introduce DNA-strand breaks. The observation of NHL-associated translocations or aberrant hypermutation preferentially involving those regions suggests that misrepair of DNA breaks during these events could also contribute to lymphomagenesis. 

Hill et al. [117] evaluated the risk of NHL in relation to 32 potential-inherited variants in DNA repair genes and found that NHL cases were more likely than controls to have a particular variant allele common in recombination genes. Shen et al. [127] report on the association between polymorphisms in DNA repair systems and NHL to in a population-based case-control study in Australia to explore potential susceptibility in exposed populations. Their study specifically implicates alkylating agents as they found a statistically significant association between MGMT and subtypes of NHL. MGMT encodes the DNA repair protein O6-methylguanine-DNA-methyltransferase (MGMT). This protein is unique among DNA repair proteins because it acts alone to remove alkyl DNA adducts. Therefore, this polymorphism may be significant with respect to exposure to alkylating agents. By contrast, Hill et al. [117] found no association in three variants of MGMT and risk of NHL in a US-based case (*n* = 1, 172) control (*n* = 982) study suggesting prevalence population admixture differences.

### 3.2. Nongenotoxic Mode of Action

Numerous studies have explored potential associations between genetic markers related to cell-cycle regulation and specific subtypes of NHL [115] and have shown mixed results with respect to concordance across studies. However, consistent associations have been found between B-cell NHL with genetic variants in pro-inflammatory factors such as TNF and leptin and the association of viral, bacterial, and other exogenous agents leading to persistent inflammation [128]. Chronic inflammation, interruption of cell cycle regulation (e.g., apoptosis or limiting apoptosis that should occur), and clonal expansion of mutated cells through increased cell proliferation represent processes by which exposure to chemicals could increase the risk of developing lymphoma as shown in the generalized schematic in Figure 5. For example, two known risk factors for NHL, cyclosporine, and azathioprine act through an immunosuppressive mode of action [129].

There is evidence of differential expression of both caspase genes and Bcl-2 family member genes among the NHL subtypes, leading to inhibition of apoptosis thereby allowing mutated cells to proliferate. This dysregulation of the balance between cell proliferation and programmed cell death [119] is a key mechanism implicated in lymphomagenesis. For example, somatic mutations in CASP3 were found in two of 129 NHL cases [130] and somatic mutations in CASP10 in 15% of 117 cases [131]. Aggressive follicular lymphoma, a subset of NHL, is associated with upregulation of genes involved in cell cycle control such as CCNE2 (cyclin E2), CCNA2 (cyclin A2), CDK2 (cyclin-dependent kinase 2), and genes-reflecting increased metabolism and DNA synthesis [115]. Bende et al. [115] report on another study which observed markedly upregulated genes including the growth factor/cytokine receptors MET (the hepatocyte growth factor receptor), FGFR3 (fibroblast growth factor receptor 3), LTBR (lymphotoxin b receptor), and PDGFRB (platelet-derived growth factor receptor b) in 11 patients with follicular lymphoma.

De Roos et al. [132] studied variation in metabolic genes in a population-based case-control study in the United States. They selected several genes known to play a role in metabolizing a broad spectrum of substrates, including pesticides, organochlorines, solvents, and PAHs, such as the phase I cytochrome P450 enzymes (CYP1A1, CYP1B1, CYP2C9, and CYP2E1), the phase II glutathione S-transferases (GSTP1 and GSTM3), and epoxide hydrolase (EPHX1). Subjects who were heterozygous or homozygous for the cytochrome P450 gene variant CYP1B1 V432L G allele were at slightly greater risk of NHL (OR = 1.27; 95% CI = 0.97–1.65); these results were consistent across B-cell lymphoma subtypes and among both Caucasians and individuals of African-American descent. The CYP2E1 1054T allele was associated with decreased risk of NHL (CT and TT genotypes combined OR = 0.59; 95% CI = 0.37–0.93), and this pattern was observed among all histologic subtypes. A systematic comparison of risks by lymphoma subtype for a broad range of risk factors in a population-based case-control study conducted by Morton et al. [133] found that immune dysfunction is of greater etiologic importance for diverse large cell B-cell lymphoma and marginal zone lymphoma than for follicular lymphoma, but that there were strong common etiologies across all NHL subtypes. This study evaluated numerous risk factors, including specific genetic polymorphisms and lifestyle and dietary characteristics and found that exposure to chlordane and PCB180 showed a relationship between exposure and specific subtypes of NHL (chlorophenoxy compounds were not evaluated).

### 3.3. Summary of NHL Studies

Given that chromosomal translocations are present in virtually all lymphomas, and very specific translocations are increasingly being identified [55], there is strong evidence that such chromosomal translocations are a required step in lymphomagenesis. As shown in Figure 5, many different kinds of endogenous and exogenous agents (including chemicals in the environment) can interact directly with DNA and cause chromosomal aberrations of this kind in pre B and mature B cells, and the t(14;18) translocation, so ubiquitous in NHL are found in 35–55% of healthy individuals [116]. Indeed, Harris et al. [55] state that chromosomal aberrations are necessary but not sufficient to actually cause NHL (page 202), consequently, there must be additional events that occur to lead to disease. Bakhshi et al. [134] state that the t(14;18) translocation “may offer a proliferative advantage but requires additional complementing genetic changes at later steps to achieve full transformation (page 2400),” as supported by Janz et al. [116] who find that chromosomal translocations are insufficient to cause disease. Morton et al. [135] find a significant association between the risk of NHL and germline variation in genes that regulate cell cycles, apoptosis, and lymphocyte development, suggesting roles for both significant genetic predisposition as well as the importance of these nongenotoxic mechanisms in disease etiology. US EPA [114] suggests that immunomodulation, related effects, and indirect effects on DNA are the primary causal factors across the lymphomas. The evidence suggests that multiple events are required to lead to NHL, most likely including some combination of chromosomal translocation coupled with proliferation of a mutation, an interruption in cell cycle regulation (e.g., failure to initiate apoptosis), or chronic inflammation. Based on observations of molecular events significant to the development of NHL specifically and lymphomas generally, the following modes of action can be hypothesized.

- Specific chromosomal aberrations (direct genotoxicity).
  - Gene-environment interaction in individuals with polymorphisms,
    - interruption of programmed cell death;
    - DNA repair mechanisms.
- Induction of enzymes implicated in the bioactivation of ubiquitous exogenous or endogenous genotoxic compounds (indirect genotoxicity); oxidative stress.
- Proliferation of mutations.
- Immunotoxic responses.

The evidence suggests a combination of molecular events is required in the etiology of NHL, including specific chromosomal aberrations which are significantly increased in agriculturally exposed individuals, but not for chlorophenoxy exposure specifically [118, 122, 124].

There is evidence that promoting activity is also required as chromosomal aberrations, particularly those associated with NHL, are prevalent in healthy individuals [63, 115], including cell proliferation of mutations, chronic inflammation, and cell cycle interruptions (e.g., failure to initiate apoptosis). There is growing evidence that germline polymorphisms contribute significantly to NHL etiology which would increase susceptibility in these individuals. It is therefore hypothetically possible that exposure to chlorophenoxy compounds in susceptible individuals could shift the risk curve (e.g., lead to increased risk at lower exposure levels as compared to nonsusceptible individuals) by a nongenotoxic mode-of-action. The evidence does not support a genotoxic or mutagenic mode-of-action; however, key transcription errors have been identified in more than half the US population; therefore, it is theoretically possible that exposure to 2,4-D/MCPA could lead to other cellular responses that, in the presence of genetic polymorphisms, might lead to increased risk.

## 4. Absorption, Distribution, Metabolism, and Elimination (ADME)

The potential for 2,4-D and/or MCPA exposures to lead to development of NHL is influenced by the efficiency with which the compounds are absorbed across different exposure routes and disposition of the compounds once in the body. Data from toxicological studies are interpreted in the context of potential exposure route, and a consideration of how chlorophenoxy compounds are absorbed, metabolized, dispersed, and eliminated once in the body. Data from laboratory studies following the time course of absorbed exposures provides important information, and these data are also used to develop different kinds of models (e.g., physiologically-based pharmacokinetic (PBPK) and others) for use in risk assessment and other assessments of environmental exposures. This section briefly describes the results of laboratory studies that have generated ADME data, and modeling studies that have used these data. 

To identify relevant citations, a literature search was conducted using PubMed, Medline, and Web of Science with the search terms “chlorophenox*,” “2,4-D,” or “MCPA” and “pharmaco*” or “metabo*.” Further studies were identified through the reference lists of studies obtained through the literature search. The search focused on primary citations in the peer-reviewed literature, although to the extent that there were some unpublished studies utilized in regulatory or other reviews; these secondary sources were summarized as well.

In general, 2,4-D and MCPA are eliminated via urine either as the unchanged parent compound (80–95%) or as conjugates, with urinary half lives on the order of one day with no evidence of oxidative metabolism in humans [25, 136] or other mammals [137]. 2,4-D and MCPA do not accumulate in tissues. 

### 4.1. Pharmacokinetics in Animals and Humans

#### 4.1.1. Absorption

MCPA and 2,4-D are readily absorbed and undergo significant but reversible plasma binding [1, 49, 137–144]. For example, Khanna and Fang (1966) explored the pharmacokinetics of ^14^C 2,4-D in male Wistar rats in two sets of experiments [144]. In the first, six rats were orally dosed with 1 mg of ^14^C 2,4-D per rat, while in the second, seven rats were administered an oral dose of 80 mg of 2,4-D. For the 1 mg 2,4-D dosage, maximum radioactivity in all tissues was reached at within eight hours of dosing and started to decrease immediately. At the 80 mg dosage, peak concentrations persisted until about 17 hours. The urine and the extracts of several tissues contained primarily unchanged 2,4-D residue. 

Studies show 2,4-D and MCPA are both readily absorbed via oral administration, but have revealed species differences in dermal absorption [145] with rats showing approximately 20% absorption and humans less than 10%. Ross et al. [145] report on an analysis of all the available data concerning dermal absorption of 2,4-D in humans based on five studies involving 34 subjects. The studies provide remarkably similar results, ranging from 1.1% to 10% absorption with a mean of 5.7%. Both the salt and acid forms of 2,4-D were evaluated with no appreciable difference, and applied doses ranged from 1.7 to 1,100 *μ*g/cm^2^. US EPA and Health Canada have both used dermal absorption values of approximately 10% for both 2,4-D and MCPA in conducting risk assessments associated with reregistration of these compounds [3, 4, 8, 9, 31–33, 146, 147].

#### 4.1.2. Distribution

2,4-D, and to a lesser extent MCPA, is highly bound to plasma proteins [49, 139, 140, 142, 148], and both are characterized by a low volume of distribution [148]. Chlorophenoxy compounds bind largely to albumin [148–150], and plasma binding is saturable at approximately 115 mg/L based on 128 blood samples from 49 patients with acute MCPA poisoning [149].

Saghir et al. [151] examined steady state levels of 2,4-D following continuous dietary dosing in rats at 5 and 100 mg/kg-day for 28 days. At 5 mg/kg, the *C*_max⁡_ blood concentration was 0.72 *μ*g/mL and was 64 *μ*g/mL at 100 mg/kg. At these dose levels, steady-state concentrations varied less than two fold over a 24 hour period. The authors suggest that the mechanism of the observed nondose-proportional increase in plasma 2,4-D concentration is likely due to high-dose-dependent saturation of the renal active anion transport clearance mechanism (the same mechanism for renal clearance of 2,4-D in humans).

Elo and Ylitalo [141] intravenously administered doses ranging from 10 to 250 mg/kg ^14^C MCPA and ^14^C 2,4-D to young and adult male Sprague-Dawley rats and determined the plasma and tissue distribution of these constituents at various times following administration. Highest concentrations were achieved approximately four hours following administration and declined thereafter with nearly complete elimination at 120 hours. At four hours, the ^14^C MCPA was nearly equally distributed between plasma, kidney, and liver. A study of the intracellular distribution of 2,4-D across six organs in rats by Khanna and Fang [144] revealed that the soluble fraction of the cells contained the major portion of radioactivity, followed by the nuclear fraction, and finally the mitochondrial and microsomal fractions.

Maximum plasma concentrations occurred within two to four hours of a 5 mg/kg orally administered dose in rats [142]. Bergesse and Balegno [152] found that radio-labeled 2,4-D uptake in Chinese hamster ovary cells was rapid and not metabolized. Uptake was pH dependent and reached a maximum at a pH of 4.5, falling to 5% of the maximum at a pH of 8.5, suggesting that uptake would be limited at *in vivo* pHs.

#### 4.1.3. Metabolism

2,4-D is excreted largely as parent compound and shows very little metabolism *in vivo* [49]. van Ravenzwaay et al. [142] found oxidation of MCPA in rats exposed *in vivo* and observed largely unchanged levels of MCPA together with low levels of the oxidation product HMCPA (4-chloro-2-hydroxymethylphenoxyacetic acid) in urine. Oxidation typically increases water solubility and therefore excretion. Bacher and Gibson [24] and Mustonen et al. [153] demonstrated the ability of MCPA and 2,4-D to induce microsomal P-450 in rat liver, while Bergesse and Balegno [152] demonstrated no metabolic activity in Chinese hamster ovary cells exposed *in vitro* to pure 2,4-D. Observed responses were at concentrations exceeding renal transport mechanisms.

#### 4.1.4. Elimination

Elimination of both 2,4-D and MCPA following oral administration is rapid and complete, occurring within 48 hours of exposure [49, 142]. Bellet et al. [1] report that in studies in rats, goats, and poultry, greater than 94% of the administered ^14^C MCPA acid was absorbed and excreted unchanged in the urine within 24 to 48 h. Renal excretion is the key elimination mechanism, and this relies on active tubular secretion and reabsorption with negligible glomerular filtration [148, 154]. Continued exposure (e.g., occupationally) results in steady-state exposures in which the amount excreted daily in urine is approximately equivalent to the amount absorbed each day [154–156].

Gorzinski et al. [138] conducted a series of acute, pharmacokinetic, and subchronic toxicological studies in rats involving technical grade 2,4-D acid, two forms of the salt, and four forms of the ester at doses ranging from 0 to 150 mg/kg-d. The concentration of ^14^C in plasma and the amount excreted in urine were proportional to dose up to doses of 50 mg/kg 2,4-D, but at 100 and 150 mg/kg, the concentration of ^14^C in plasma was greater than expected based on the lower doses indicating saturation of renal clearance mechanisms above approximately 50 mg/kg in the rat, similar to the results obtained by van Ravenzwaay et al. [49]. 

Lappin et al. [139, 140] orally administered ^14^C MCPA to rats and dogs at 5 or 100 mg/kg in order to explore differences in plasma toxicokinetics, rates and routes of excretion and biotransformation. Elimination of radioactivity was biphasic in rat plasma and monophasic in the dog. For both species, the principal route of excretion was via urine, but renal elimination was notably more rapid and more extensive in the rat. In both rat and dog, excretion of radioactivity was mainly as MCPA and its hydroxylated metabolite (HMCPA). In the rat, both were mainly excreted as the free acids, although a small proportion was conjugated. In the dog, the proportion of HMCPA was increased, and the majority of both species were excreted as glycine or taurine conjugates. These data, along with previously published accounts indicate that renal elimination of MCPA in dogs is substantially slower than in rats. These pharmacokinetic differences indicate that studies in dogs are not relevant for potential human health effects [137].

Sauerhoff et al. [25] conducted a study in five male human volunteers who ingested a single dose of 5 mg/kg 2,4-D without detectable clinical effects. Concentration of 2,4-D was determined in plasma in three of five subjects and in urine in all subjects at timed intervals. The elimination of 2,4-D from plasma in all subjects occurred by an apparent first-order rate process with an average half life of 11.6 h. All subjects excreted 2,4-D in the urine with an average half life of 17.7 h. Excretion occurred mainly as 2,4-D (82.3%) with smaller amounts excreted as a 2,4-D conjugate (12.8%). Essentially all of the 2,4-D were absorbed from the gastrointestinal tract in man. No evidence of nonlinear kinetics was observed following the 5 mg/kg oral dose of 2,4-D.

Knopp [154] followed 27 men and 18 women over a five-year period (1985–1989) and measured urinary and serum levels in order to estimate excretion rates. Following five days of exposure during the work week, the author found that urinary concentrations decreased dramatically over a weekend of no exposure and returned to steady state during the following week of exposure, consistent with rapid clearance of 2,4-D from the body.

### 4.2. Pharmacodynamics

Dierickx [157] explored the *in vitro* interaction of 2,4-D, MCPA, MCPP, and 2,4-DP with rat-liver glutathione S-transferase (GST) using reduced glutathione and l-chloro-2,4-dinitrobenzene as substrates and found significant, dose-dependent inhibition of GST activity across compounds, albeit at concentrations of approximately 0.1 mM or 22 *μ*g/mL, and a plasma concentration saturating renal clearance. Ring substitution and side-chain length were shown to be of importance in determining the extent of GST inhibition. GST AA, an isoenzyme of GST, was stimulated by MCPA and 2,4-D. The author concludes that MCPA and 2,4-D interact with GST by binding directly to these proteins, and this may have a protective function against these herbicides. 

Bukowska et al. [158] explored the effects of exposure of human erythrocytes to different concentrations of MCPA and its environmental metabolite: 2,4-dimethylphenol (2,4-DMP) with respect to glutathione content (GSH and GSSG), glutathione peroxidase (GSH-Px), glutathione transferase (GST), and the level of adenine energy charge (AEC). GSH protects cells from oxidative damage caused by free radicals. MCPA (250 ppm) decreased the level of GSH in erythrocytes by 9.2% and 2,4-DMP by 33.3% in comparison with controls at 250 and 500 ppm but not at lower concentrations, and this decrease was not statistically significant. Glutathione transferase activity was not altered for any compound across all concentrations tested. 

Palmeira et al. [159–162] conducted a series of studies using rat hepatocytes and found that at concentrations starting at approximately 200 *μ*g/mL, 2,4-D induced time and dose-dependent cell death accompanied by depletion of GSH. 

A significant fraction of the absorbed dose of 2,4-D and MCPA circulates in plasma before being excreted, or in the case of low-level chronic exposures, concentrations in plasma will reach steady state levels relative to exposures. Consequently, it is important to understand the potential effects of circulating 2,4-D and/or MCPA on cell structure and function. Several studies have evaluated the ability of 2,4-D, MCPA, and other chlorophenoxy compounds to cause cellular damage that may be relevant to a toxic mode of action, including hemolysis, hemoglobin oxidation, and lipid peroxidation [151, 163–166]. The concentrations at which these kinds of effects are typically noted, however, tend to be above concentrations at which renal saturation occurs. Kozuka et al. [163] examined the *in vivo* effects of MCPA, 2,4-D, and several other chlorophenoxy compounds on peroxisomal fatty acid oxidation-related enzymes in rat liver and found a significant increase in hepatic peroxisomal fatty acid oxidation in male Wistar rats orally exposed to 150 mg/kg-d 2,4-D for two weeks, while no effects were observed for MCPA, again, at concentrations exceeding renal saturation.

In another series of studies to evaluate potential cellular damage caused by 2,4-D and MCPA, and their metabolites, Duchnowicz et al. [164–166] exposed human eyrthrocytes to concentrations of 2,4-D and MCPA ranging from 1 mM (200–221 *μ*g/mL) to 4 mM (1,000–1,105 *μ*g/mL). Effects, ranging from ATPase activity to lipid peroxidation, were only observed in a few instances at concentrations greater than 1 mM and typically at concentrations greater than 4 mM. Hemolysis was not increased. Duchnowicz et al. [165] found that exposure of human erythrocytes to 220 ppm 2,4-D and MCPA caused an increase in ATPase activity relative to controls that decreased relative to controls at higher tested concentrations (440 and 884 ppm); however, these high concentrations are not informative with respect to *in vivo* population exposures. 

Bukowska [167] explored the effect of 2,4-D, MCPA, the derivatives phenol, 2,4-dichlorophenol (2,4-DCP), 2,4-dimethylphenol (2,4-DMP), and catechol on the activity of acetylcholinesterase (AChE, EC3.1.1.7) in human erythrocytes. AChE activity is considered an indicator of the ability of an exposure to cause membrane damage. Phenol, MCPA, and 2,4-DMP did not significantly change AChE activity in human erythrocytes while decreases in AChE activity were observed under the highest applied dose of 2,4-D at 500 and 1000 ppm. 

Bukowska et al. [168] investigated the effect of the sodium salt of 2,4-D (2,4-D-Na) and sodium salt of MCPA (MCPA-Na) on the oxidation of dihydrorhodamine 123 and H2DCFDA, carbonyl group content in cellular proteins, and hemoglobin denaturation. The rate of fluorescent probe oxidation was significantly higher for 2,4-D-Na, while both compounds increased the contents of protein carbonyl groups. No changes in the denaturation of hemoglobin were observed. 2,4-D-Na induced H2DCF oxidation in human erythrocytes in a linear dose response up to four hours. MCPA-Na did not induce H2DCF oxidation even at the highest concentration during 3 h of incubation. Statistically significant changes were observed only for MCPA and 2,4-D at approximately 500 ppm following 24 h of incubation. The authors found that only 1% of the MCPA and 2,4-D used in this experiment penetrated the cell membrane, requiring significantly higher concentrations than would be experienced *in vivo* even occupationally and clearly exceeding renal transport mechanisms in humans. 

Bukowska et al. [169] investigated the effect of 2,4-D on catalases in human erythrocytes at 100, 500, and 1000 ppm over one hour, three hours, and 24 hours. Catalases are important in eliminating the potentially dangerous formation of free radicals in cells, thus a decline in catalase activity could be significant with respect to protecting cells. The authors found a small but statistically significant decline in catalase activity from exposure to 2,4-D and MCPA at the highest concentration (1000 ppm) but not at lower concentrations, and only after 3 hours for 2,4-D and 24 hours for MCPA. Similarly, Kaioumova et al. [170] found that 2,4-D salt was able to cause apoptosis in peripheral blood lymphocytes of healthy individuals and Jurkat T cells in a dose and time dependent manner, but only at concentrations leading to acute-poisoning effects *in vivo*.

2,4-D and MCPA have both been shown to induce peroxisome proliferation [137, 171, 172] but neither showed any agonistic activity via PPAR*α* in *in vitro* reporter gene assays with CV-1 cells [173]. Maloney and Waxman also reported that 2,4-D and MCPA were inactive in the human and mouse PPAR*α* activation assays using simian renal carcinoma COS-1 cells [174]. Thus, although 2,4-D and MCPA have been shown to be peroxisome proliferators, they do not interact with PPAR*α* in cell culture systems, and *in vivo* conversion to more toxic metabolites, a potential pathway for induction of carcinogenic effects of peroxisome proliferation, has not been shown to occur. In addition, concentrations at which proliferation has been observed have been in excess of 100 ppm, well in excess of renal transport mechanisms in humans.

Under the US EPA high-throughput screening ToxCast program, both MCPA and 2,4-D were screened in concentration-response format across more than 500 cell based and biochemical assays [172, 202].  Table 5 provides a brief description of the six positive assay results for MCPA and the eight positive assay results for 2,4-D. The level shown is the lowest effective concentration in *μ*M to elicit the response. A change in cell growth kinetics is the most sensitive assay for 2,4-D, while upregulation of CD38 expression is the most sensitive assay result for MCPA. CD38 is a transmembrane glycoprotein highly expressed in B cell lymphoma (http://www.ihop-net.org/UniPub/iHOP/gismo/87033.html) and is known to prevent apoptosis in germinal center B-cells. This may be a relevant pathway with respect to proliferation of key chromosomal aberrations. Significantly increased expression of CD38 has been observed across several NHL subtypes, although the mechanistic relevance is unknown. 

ToxCast assay results show that MCPA led to increased expression of CYP2B6, one of the P450 enzymes. De Roos et al. [132] found increased risk for developing NHL for certain CYP variants, although they did not evaluate this specific variant.

The change in cell growth kinetics observed for 2,4-D may also suggest a relevant pathway, although the specific change observed in the assay is not readily identifiable with respect to an *in vivo* response. Given that MCPA is a close structural analog to 2,4-D, one might have expected greater concordance across ToxCast assay results between the two compounds. The implications of differing ToxCast assay results remain unknown.

### 4.3. Summary of Pharmacokinetic and Pharmacodynamic Studies

- 2,4-D and MCPA are readily absorbed and excreted largely as parent compound via active tubular transport and reabsorption.
- Saturation of renal clearance occurs at approximately 50 mg/kg in male rats, resulting in nonlinear increases in plasma concentrations.
- Both 2,4-D and MCPA undergo saturable and reversible protein (primarily albumin) binding.
- Approximately 10% or less of dermally applied doses are absorbed in humans (up to 20% in mice).
- 2,4-D and MCPA induce P450 in rodents at concentrations that are readily excreted.
- Human erythrocytes incubated in greater than 110 *μ*g/mL of MCPA and 2,4-D showed membrane damage, lipid peroxidation, and a decrease in-SH groups; translating to *in vivo* concentrations exceeding saturation of renal clearance (e.g., readily excreted).
- 2,4-D and MCPA lead to cytotoxicity in hepatocytes and interact with liver mitochondrial energetics starting at approximately 200 *μ*g/mL (e.g., concentrations exceeding saturation of renal clearance and therefore readily excreted).
- 2,4-D and MCPA have been shown to be peroxisome proliferators at concentrations exceeding renal transport mechanisms but do not generate metabolites and showed no agnostic activity via PPAR*α*.
- MCPA tested positive in only six of over 500 ToxCast assays and 2,4-D in eight; the assays related to cell growth and proliferation may be relevant for increased risk of lymphoma. However, there is little concordance across ToxCast results between the two compounds, and corresponding *in vivo* concentrations are high.

## 5. Toxicological Studies

This section summarizes the available literature for toxicological studies, either conducted *in vivo* or *in vitro* in animals, or using *in vitro* human cell cultures or through *in vivo* human exposures.  Table 6 provides a summary of the *in vivo* animal studies for 2,4-D and MCPA, while Table 7 focuses on *in vitro* studies using nonhuman cell cultures.  Table 8 provides a summary of studies based on either *in vivo* human exposures or *in vitro* human cell cultures. Studies are discussed in the context of the potential modes of action. 

Toxicological studies provide an important link back to the observational studies in humans by allowing for a more detailed evaluation of hypothesized modes of action. What is difficult here with respect to exposures to 2,4-D and/or MCPA is that none of the standard rodent bioassays demonstrate carcinogenicity of these compounds. In evaluating the potential for exposures to lead to carcinogenic outcomes, generally there is a discussion of the concordance across outcomes in animals and humans, and the standard *in vivo* toxicological rodent bioassays represent an important link between potential effects in animals versus humans. But in this case, there are no relevant tumorigenic outcomes in animals based on a series of assays conducted in support of the pesticide registration process. There are a number of assays and observations *in vivo* and *in vitro* at the cellular level (e.g., sister chromatid exchange, micronucleus formation, etc.) that in some cases do show negative responses following exposures, but in the absence of frank tumor formation in rodents using standard multiyear and multigenerational assays, the implication of those results require cautious interpretation. 

Initial candidate studies are identified through an online search of PubMed, Medline, and Web of Science using search terms including “chlorophenox*,” “health,” and “effect,” or “toxicol*.” Further studies are identified through careful review of the reference lists of studies obtained through the literature search and of the reference lists of prior reviews (particularly for the older studies).

Toxicological studies are evaluated using the following set of questions.

*Does the assay involve in vivo human exposures or human cell lines?* Given the lack of tumorigenic responses across standard rodent bioassays, this evaluation considers i*n vivo* and *in vitro* responses in humans and human cell systems to be more relevant than responses in animal systems. For example, a study documenting cellular responses following *in vivo* exposures is given greater weight than a strictly *in vitro* study or a similar study in animals.

*Is there evidence for direct DNA interaction, and if so, based on which assays?* Butterworth [203] outlines a battery of typical *in vivo* and *in vitro* tests and provides a rationale for evaluating the results of specific assays. For example, results from bacterial mutagenicity, *in vivo *mouse bone marrow micronucleus tests, and human lymphocyte chromosomal aberration assay results should be considered to provide stronger evidence of mutagenic potential than the CHO chromosomal aberration assay and the mouse lymphoma mutagenicity assay because of the high false positive rate of the latter two assays. Moreover, the combination of positive assays and cell proliferation provides stronger evidence of potential carcinogenicity as DNA replication is required to convert a DNA adduct into a permanent mutation. A genotoxic chemical administered at a toxic dose that also induces cell proliferation will be far more effective as a mutagen and as a carcinogen than when given at a low dose that does not induce cell proliferation [203, 204]. 

*Is there evidence for non-DNA reactive carcinogenicity? *Chronic inflammation, nuclease release, sustained stimulation of regenerative cell proliferation, and interruptions to cell cycle regulation and function all provide evidence of a non-DNA reactive mode of action [204].

*What are the in vivo exposures implied by the study, and how do those relate to known exposures from the biomonitoring and PBPK studies?* Particularly with respect to the *in vitro* studies, the concentrations at which responses are observed can translate to *in vivo* exposures that exceed systemic toxicity and/or renal clearance mechanisms, or that require exposures that are much greater than occur during typical product use. A key element here is the fact that dermal exposures are the predominant exposure pathway given chlorophenoxy use and properties, and studies show that approximately 10% of a dermally applied dose is absorbed [145]. It is then possible to ask how much of the constituent would need to be sprayed or used to lead to a dermal exposure at which effects might be observed, and do the data suggest these kinds of exposures are realistic given what is known about product use.

### 5.1. Toxicological Studies in Animals

In general, standard two-year or multigenerational *in vivo *toxicological studies in rats and mice show virtually no evidence of treatment-associated carcinogenicity and very little in the way of histological effects resulting from sustained exposure to these constituents. The primary effects noted *in vivo* relate to organ weights and some limited hyperplasia. The US EPA human health risk assessment developed to support the reregistration decision of MCPA finds that MCPA is “not mutagenic” and “unlikely to be a carcinogen” [3], while it considers 2,4-D “unclassifiable” with respect to carcinogenicity [8]. Accordingly, there are no published slope factors in IRIS for either constituent. 

Section 5.1.1 provides a brief summary of the standard rodent bioassays, while Section 5.1.2 presents the data for genotoxicity in animal systems. 

#### 5.1.1. Rodent Bioassays

**MCPA**

The published RfD for MCPA is 0.0005 mg/kg-day based on a study of technical grade MCPA orally administered to male and female beagle dogs (6/sex/dose) at doses of 0, 6, 30, or 150 ppm (0, 0.15, 0.75, or 3.75 mg/kg/day) for 52 weeks (unpublished 2,4-D Task Force study). This dosage resulted in kidney and liver toxicity at the mid- and/or high-dose levels, with alterations in clinical chemistries (kidneys: urea, potassium, and creatinine; liver: bilirubin, GPT, GOT, triglycerides, and cholesterol) associated with concomitant organ weight changes (liver) and histopathology changes (kidney: increased kidney pigment deposition in proximal tubular epithelium; liver: change in the nature/coloration of gall fluid). Therefore, based upon kidney and liver toxicity at the 30 and 150 ppm dose levels, the lowest effect level for systemic toxicity was determined to be 0.75 mg/kg/day. The NOEL for systemic toxicity was estimated at 0.15 mg/kg/day, with an uncertainty factor of 300 to obtain the RfD.

Bellet et al. [1] report on two chronic toxicity/oncogenicity studies. In the first, MCPA was administered to 50 male and 50 female Wistar rats at doses of 0, 20, 80, and 320 ppm in the diet for 24 months. There was no effect observed on mortality in any dose group. Responses noted at 320 ppm included slight but statistically significant decreases in body weights in males as compared to controls. Small changes in clinical chemistry parameters were also noted in males as compared to females from the same dose group. Spontaneously occurring nephropathy was more pronounced in male rats in the 320 ppm dose group, including an increase in the retraction and granular surface of the kidneys. In female rats, a statistically significantly higher mean absolute kidney weight of female rats from the 80 ppm dose group was noted. However, there was no weight change in the high-dose females, and no histological effects observed in female rats at any dose level. The systemic NOEL was estimated at 20 ppm (approximately 1.3 mg/kg/day) for both male and female rats based upon the nephrotoxicity observed in either the 80 or the 320 ppm dose groups. No carcinogenic or oncogenic responses were observed across all doses.

In the second study, MCPA was administered in the diets of 50 male and 50 female B6C3F1 mice at doses of 0, 20, 100, or 500 ppm over a two-year period. There was no effect observed on mortality across dose groups. Reduced body weight gain was noted throughout the study in male mice receiving 500 ppm MCPA, except for the last six months. Both absolute and relative kidney weights showed a statistically significantly increase in female mice from the high-dose group, which was associated with histopathological changes noted in the kidneys in both sexes. Male and female mice in the highest-dose group showed an increased incidence of intratubular calcification and tubular hyaline-proteinaceous casts. Male mice only showed a statistically significant increase in the incidence of renal tubular epithelial focal hyperplasia in the 500 ppm dose group. The systemic NOEL was estimated at 100 ppm. No carcinogenic or oncogenic responses were observed across all doses.

**2,4-D**

The published Reference Dose (RfD) for 2,4-D in the US EPA Integrated Risk Information System (IRIS; http://www.epa.gov/iris/) is 0.01 mg/kg-d based on a no observed adverse effect level of 1 mg/kg-d from a 13-week subchronic study in rats. At 5 mg/kg-d, there were statistically significant reductions in mean hemoglobin (both sexes), mean hematocrit and red blood cell levels (both sexes), and mean reticulocyte levels (males only) after seven weeks. There were also statistically significant reductions in liver enzymes LDH, SGOT, SGPT, and alkaline phosphatase at week 14 in animals treated at the 15.0 mg/kg-day or higher doses. This RfD is currently in review.

The US EPA Office of Pesticide Programs used an RfD of 0.005 mg/kg-d based on an observed NOAEL of 5 mg/kg-d as documented in the reregistration document [8, page 21].

Charles et al. [6, 7, 176] conducted a series of chronic and subchronic toxicity studies using all three forms of 2,4-D (the acid parent compound, the salt and the ester). The first chronic toxicity study in male and female Fischer rats found no effects of exposure. The subchronic study (13 weeks) found decreased red blood cell and platelet counts and decreased circulating T3 and T4 levels at the 100 mg/kg-d dose level. In contrast, Gorzinski et al. [138] in a 13-week subchronic study with rats found statistically significantly decreased T4 levels at 100 mg/kg-d, but statistically significantly increased T4 levels at 15 mg/kg-d.

Charles et al. [176] reported no changes across immunotoxicological parameters including bone marrow and/or lymph node histopathology or leukocyte counts in beagle dogs in a one-year feeding study with doses ranging from 1 mg/kg-d to 7.5 mg/kg-d. The subchronic (13 weeks) portion of the study established a NOEL of 1 mg/kg-d. Increases in blood urea nitrogen, creatinine, and ALT were consistently observed across the studies. With respect to chronic effects, the study noted no other effects at one year that were not already evident at 13 weeks; thus, the authors argue for a chronic NOEL of 1 mg/kg-d based on this study.

Blakley et al. [177] exposed male CD-1 to a commercial amine derivative of 2,4-D in drinking water to evaluate the effect of 2,4-D on the incidence of spontaneous murine lymphocytic leukemia over a 365 day treatment period and found that mortality associated with the leukemia was not impacted in any way by the 2,4-D treatment. In another study, Blakley et al. [178] evaluated immune function in male Fischer 344 rats exposed to 10 mg/kg of 2,4-D by oral gavage twice weekly for four weeks and found no effect on lymphocyte blastogenesis, lymphocyte cell surface marker expression, or phagocytic function of peritoneal macrophages.

The only evidence for carcinogenicity of 2,4-D in animal studies is based on a case-control study of pathologically confirmed cases of lymphoma in dogs [205]. This study was subsequently reviewed by an expert panel [206], who identified significant limitations associated with the study and concluded no association between 2,4-D exposure and canine lymphoma given similar limitations as the human epidemiologic studies (e.g., exposures were poorly understood and specific exposures only qualitatively identified, no observed dose-response relationship, etc.). Kaneene and Miller [207] reanalyzed the Hayes et al. [205] data using the exposure definition used in the original study, reanalyzed the data using a redefinition of exposure, conducted a dose-response analysis with the redefined exposure criteria, and did not confirm a dose-response relationship between 2,4-D use and lymphoma in dogs. The reanalysis found no significant association between 2,4-D exposure and canine lymphoma.

Reynolds et al. [208] showed that dogs exposed to lawns following various types of herbicide treatment do show measurable urinary levels of 2,4-D (on the order of 10 *μ*g/L and some as high as 50 *μ*g/L ten days following application). A recent study [209] found a statistically-significant association between the use of “self-applied insect growth regulators” (OR = 2.7; 95% CI = 1.1–6.8) and canine lymphoma, but specific constituents were not identified. Associations between herbicides were not statistically significant (OR = 1.3; 95% CI = 0.9–1.8).

The only available study that evaluated any effects following dermal exposures is Schop et al. [180] who exposed male CD1 mice dermally to 500, 1000, and 2000 *μ*Mol/kg-day (approximately 110, 220, and 442 ppm, resp.). They compared the results of the bone marrow micronucleus test and hair follicle nuclear aberration assay conducted 24 hours following topical application of 2,4-D and found no effect in the micronucleus test, and a statistically significant increase of a 2% increase relative to controls in the nuclear aberration assay (at the site of exposure). Concentrations of 2,4-D were not measured in the animals; however, Ross et al. [145] report that mice show amongst the highest dermal absorption proportion relative to exposed dose (20%). Consequently, the 2,4-D should have been completely absorbed in 24 hours.

#### 5.1.2. Genotoxicity Based on *In Vitro* or *In Vivo* Animal Studies

A hypothesized mode of action is that exposure to 2,4-D and/or MCPA leads to direct DNA damage. Specific chromosomal translocations have been observed across the lymphomas, particularly NHL, and evidence is increasing that these are a prerequisite for disease. This section evaluates the data on genotoxicity for these two compounds based on laboratory studies in animals.

**MCPA**

Bond and Rossbacher [39] report that MCPA did not cause point mutations when tested in the Ames test or the host mediated assay or in mammalian (V79) cells. No increase occurred in chromosomal aberrations in Chinese hamster bone marrow cells after oral exposure of the animals. A weak increase in the rate of SCE was found in the same animal strain at toxic doses *in vivo*, whereas at lower doses there were no adverse effects observed. A DNA-binding study of radiolabelled MCPA did not show any interaction of the compound with the genetic material of the liver cells. Other test systems showed equivocal results (SLRL test, assays in yeast cells). Considering all mutagenic studies carried out with MCPA, it can be concluded that most tests were negative, but that in some tests a weak mutagenic potential was found at doses that would lead to acute toxicity *in vivo*.

Elliott [20] similarly conducted a review of available *in vivo* (*n* = 12) and *in vitro* (*n* = 13) assays to evaluate the mutagenic and genotoxic potential of the amine salt form of MCPA. Elliott concludes that MCPA is nonmutagenic across bacterial and mammalian cell gene mutation assays. He notes increases in percentage aberrant cells found on analysis of metaphases of human peripheral lymphocytes treated *in vitro* in the presence of auxiliary metabolic activation (S9), but only at doses approaching 10 mM and leading to significant cytotoxicity. The fact that metabolic activation was required suggests that this effect would not be noted *in vivo*. No evidence for clastogenicity *in vivo* was found in the mouse bone marrow micronucleus assay or the Chinese hamster bone marrow metaphase assay. No evidence for either increases in SCE frequency or DNA binding was found in the rat. Very small (less than 1.5 times controls) increases in SCE were observed *in vivo* in the hamster at toxic or maximum tolerated dose levels. Elliott [20] concludes there is no *in vivo* or *in vitro* evidence for mutagenicity of MCPA, particularly the salt forms of the compound typically used in commercial products.

**2,4-D**

Amer and Aly [179] treated six Swiss mice by oral gavage with 2,4-D at 1.7, 3.3, and 33 mg/kg. 2,4-DCP, the 2,4-D metabolite, was intraperitoneally injected at 36, 72, and 180 mg/kg. Oral treatment by gavage with the lowest tested dose (1.7 mg 2,4-D kg^−1^ BW) for five consecutive days had no significant effect on the induction of chromosomal aberrations, but a significant increase in the percentage of chromosomal aberrations in bone-marrow and spermatocyte cells was observed after oral administration of 2,4-D at 3.3 mg/kg bw for three and five consecutive days. The number of observed chromosomal aberrations was a factor of four higher for the positive control injected with mitomycin C as compared to the 2,4-D exposed animals, and the number of chromosomal aberrations was not dose dependent. 2,4-DCP injected animals only showed a response at the highest dose tested (180 mg/kg).

Charles et al. [181] conducted *in vitro* unscheduled DNA synthesis assays on male Fischer 344 rat hepatocytes using parent 2,4-D compound and seven derivatives of salts, esters, and amines. Plate concentrations ranged from 2 to 340 *μ*g/L, and no effects were observed. In the same study, the authors also conducted the Ames bacterial reverse mutation assay (including a positive control) and found no effects across all treatment types. A follow-on study [185] evaluated the potential for 2,4-D and seven of its salts and esters to induce cytogenetic abnormalities in mammalian cells *in vivo* using the mouse bone marrow micronucleus test in CD-1 mice. All the test materials were administered to male and female mice by oral gavage, and the frequencies of micronucleated polychromatic erythrocytes MN-PCE in bone marrow were determined at intervals of 24, 48, and 72 h following dosing. There were no significant increases in the incidence of MN-PCE in the treated mice at any of the bone marrow sampling times. Five animals per group were sacrificed at either 2–4 h, or 12–14 h, after dosing. Treatment with 2,4-D at doses up to 1000 mgkg demonstrated no effects on rat hepatocytes.

Maire et al. [188], after 5 h of treatment, the percentage of SHE cells with damaged DNA was 8% in class 1, and 0.7% in class 2 (percentage of DNA in the tail between 40% and 60%) after exposure to 11.5 M 2,4-D. After 5 h of treatment at 23 M, the percentage of DNA-damaged cells was 12.3% in class 1 and 1.3% in class 2. After 24 h of treatment at 11.5 M 2,4-D, 9.7% of cells ranked in class 1 and 0.3% in class 2. At 11.5 M 2,4-D, 17% of cells ranked in class 1, 5.3% in class 2, while 1.3% of cells were in class 3 with a high level of DNA breaks (percentage of DNA in the tail between 60% and 80%). After 2 h of treatment with the positive control H2O2 (500 M), the percentage of SHE cells with DNA damage was 18% in class 1, 10% in class 2, while 67% of the cells were in class 3, with a high level of DNA breaks.

Gollapudi et al. [18] investigated the genetic toxicity of 2,4-D 2-butoxyethylester and two salts (2,4-D isopropylamine and 2,4-D triisopropanolamine) in cultured Sprague-Dawley rat cells. The end points used were the induction of chromosomal aberrations in primary cultures of rat lymphocytes and forward mutations at the HGPRT locus of Chinese hamster ovary (CHO) cells with and without S9 activation. There was no evidence of genotoxicity across test materials. However, Gonzáles et al. [186] evaluated the potential genotoxicity of 2,4-D and a commercially used derivative, 2,4-D dimethylamine salt (2,4-D DMA) in CHO cells using SCE, and single cell gel electrophoresis (SCGE) assays and found significant dose-dependent increases in SCE, regardless of the harvesting time (2,4-D: *r* = 0.98 and *r* = 0.88, *P* < 0.01, for 24 and 36 h harvesting times; 2,4-D DMA: *r* = 0.97 and *r* = 0.88, *P* < 0.01, for 24 and 36 h harvesting times). Log-phase cells were treated with 2.0–10.0 *μ*g/mL of herbicides and harvested 24 and 36 h later for SCE analysis. Neither test compound altered cell cycle progression or proliferative replication index (*P* > 0.05), but the higher doses of both compounds reduced the mitotic index of cultures harvested at 24 and 36 h (*P* < 0.05). A 90-min treatment with 2.0–10.0 *μ*g/mL 2,4-D and 2,4-D DMA produced dose-dependent increases in the frequency of DNA-strand breaks detected in the SCGE assay, both in cultures harvested immediately after treatment and in cultures harvested 36 h later. The doses of 2,4-D and 2,4-D DMA were equally genotoxic in all of the assays. By contrast, Linnainmaa [19] reported no increase in SCE frequency after a 1 h pulse treatment of CHO cells with pure 2,4-D and a commercial 2,4-D formulation (2,4-D amine salt as the active ingredient) with and without S9 activation.

Gonzáles et al. [186] evaluated the potential genotoxicity of pure 2,4-D (acid), and the commercially used salt in Chinese hamster ovary cells treated with 2.0–10.0 *μ*g/mL using SCE and single cell gel electrophoresis (SCGE) assays. The authors found that both forms of 2,4-D induced significant dose-dependent increases in SCE, but neither test compound altered cell cycle progression or replicative index. The highest doses of both forms of 2,4-D reduced the mitotic index of cells.

### 5.2. Studies in Humans and Using Human Cell Cultures

Table 8 summarizes the available data for assays involving human cell cultures or cells from humans exposed *in vitro* and *in vivo*. Several studies, particularly those based on field exposures *in vivo*, do not explicitly distinguish between MCPA and 2,4-D exposures, and results are considered applicable to both. In general, observing an *in vivo* effect in humans takes precedence, although lack of an effect *in vivo* does not negate a positive *in vitro* effect. At that point, it is important to consider the conditions under which exposure across test systems, and how those relate to environmental exposures. 

#### 5.2.1. Genotoxicity

Mustonen et al. [193] evaluated chromosomal aberrations *in vivo* in lymphocyte cultures from 19 exposed 2,4-D and MCPA Swedish forestry sprayers. Workers sprayed 333 g/L 2,4-D and/or 167 g/L MCPA during July through October 1981 for a minimum of six days and a maximum of 28 days. No increase in the incidence of chromosomal aberrations in the lymphocytes of workers was observed in this study. These authors also conducted an *in vitro* study in which human peripheral lymphocytes were cultured with 0.125, 0.250, 0.500, 1.000, and 1.250 mM of pure 2,4-D as well as a commercial herbicide containing 2,4-D (*Vesakontuho Tasku* containing 550 g/L 2,4-D as amine salt in water). The pure 2,4-D product showed no induction of chromosomal aberrations of any kind, but the commercial mixture showed statistically significant differences from controls in a dose-dependent manner starting at 0.5 mM (110 ppm). The authors suggest this is due to impurities and phenols contained in the commercial mixture. However, Clausen et al. [194] and Jacobi and Witte [195] in separate studies involving a commercial formulation of 2,4-D argue that observed differences in toxicity may be attributable to differences in chemical structure between the pure acid and the soluble salt, although this seems unlikely as the soluble salt disassociates to the pure acid under physiological conditions. 

In an *in vivo* study in forestry workers spraying foliage with either 2,4-D, MCPA, or a mixture, Linnainmaa [210] found no induction of SCEs in peripheral lymphocytes. SCE analyses were conducted on cells from 35 herbicide workers and 15 control subjects. No statistically significant differences in the frequencies of SCEs were observed in samples taken before, during, or after the exposure, and the mean SCE from nonexposed control group fell in the same range as those of the exposed subjects.

**MCPA**

Elliot [20] conducted a literature review of available genotoxicity and mutagenicity studies involving MCPA and find no evidence of these effects in human cell cultures. MCPA was not genotoxic in a battery of assays developed under the US EPA high-throughput screening ToxCast program [211].

**2,4-D**

Korte and Jalal [191] evaluated the clastogenic and mutagenic potential of 2,4-D in cultured lymphocytes. Chromosomal damage, though statistically insignificant, occurred at doses as low as 0.2 *μ*g/mL and increased at a statistically significant level at concentrations of 50 *μ*g/mL or higher. Potential mutagenicity, based on rates of increase in SCE, was significant at 10 *μ*g/mL or higher concentrations. In a similar study, Turkula and Jalal [192] observed a weak increase in SCE in peripheral human lymphocytes exposed *in vitro* at 50, 100, and 250 *μ*g/mL, but the difference was only statistically significant at the lowest dose, and the increase was less at higher doses than at the lowest dose.

Soloneski et al. [22] explored the genotoxic potential of 2,4-D and its commercial derivative 2,4-D DMA by measuring sister chromatid exchange (SCE), cell cycle progression, and mitotic index in human whole blood (WBC) and plasma leukocyte cultures (PLC). Cells were exposed to concentrations of 10, 25, 50, and 100 *μ*g/mL for 72 h. SCE frequency was statistically significant increased at concentrations from 10 to 50 *μ*g/mL for 2,4-D and from 25 to 100 *μ*g/mL for 2,4-D DMA. However, in PLC, there was no observed increase in SCE. A significant delay in cell proliferation was observed in WBC after treatments with 25 and 50 *μ*g/mL 2,4-D and 50 and 100 *μ*g/mL 2,4-D DMA, whereas in PLC, only 100 *μ*g/mL 2,4-D altered cell-cycle progression. For both chemicals, a progressive dose-related inhibition of mitotic activity was observed. The results demonstrated that the presence of erythrocytes in the culture system appeared to increase DNA and cellular damage inflicted by 2,4-D and 2,4-D DMA. However, again these concentrations are high relative to environmental exposures.

Under the US EPA ToxCast program, a suite of chemicals, including 2,4-D, was tested in 467 assays including assays for genotoxicity [202] and found not to be genotoxic across a suite of assays [211]. The US EPA has reviewed the potential genotoxicity and mutagenicity of 2,4-D [5, 27], most recently in 2012 [33]. Those data show no evidence for heritable mutagenic effects in mammals but some evidence supporting 2,4-D's potential to cause genotoxic effects. Specifically, U.S. EPA concluded that the combined evidence shows: (1) 2,4-D is negative across bacterial mutation assays; (2) some positive results for mutagenicity in assays in yeast, plants, and insects; (3) negative results for mutagenicity based on *in vivo* mammalian studies; (4) mixed results for mutagenic and genotoxic results based on mammalian *in vitro* tests.

#### 5.2.2. Proliferative and Immunological Effects

In a study involving ten farmers who mixed and applied 2,4-D and MCPA for one to three days, Faustini et al. [200] collected blood samples from ten farmers within seven days prior to exposure to 2,4-D. Samples were collected again from one to 12 days after exposure and again from 50 to 70 days after exposure. Whole blood was used to count lymphocyte subsets with monoclonal antibodies. Peripheral blood mononuclear (PBM) cells were used to measure natural killer (NK) cell activity and lymphocyte response to mitogenic stimulations. Individual values collected prior to exposure were used as reference. Relative to concentrations prior to exposure, a significant reduction was found from one to 12 days after exposure in the following variables (*P* < 0.05): circulating helper (CD4) and suppressor T cells (CD8), CD8 dim, cytotoxic T lymphocytes (CTL), natural killer cells (NK), CD8 cells expressing the surface antigens HLA-DR (CD8-DR), and lymphoproliferative response to mitogen stimulations. All immunological values found 50–70 days after exposure were comparable with concentrations before exposure, with the exception of the percentage of CD8-DR cells, which continued to be statistically significantly decreased. Although exposures to chlorophenoxy compounds are episodic, there may be long-term implications associated with repeated, short-term immunosuppression in cancer etiology. No correlation was found between kg of pesticide applied (which ranged from 12 to 155 kg across the ten participants) and immunological measures.

**MCPA**

Elliot [20] reports on three studies using MCPA and peripheral human lymphocytes *in vitro* in which demonstrated cell cycle delays at concentrations greater than 500 *μ*g/mL (original studies were not available from the primary literature).

**2,4-D**

Tuschl and Schwab [197] and Kaioumova et al. [170] were able to induce apoptosis by exposing HepG2 cells and human lymphocytes, respectively, *in vitro* for several days. However, these effects were only observed at high concentrations (above 884 *μ*g/mL and 660 *μ*g/mL, resp.). In theory, induction of apoptosis could be beneficial in individuals with an existing t(14;18) translocation since that leads to inhibition of apoptosis. However, these concentrations are too high to be relevant to *in vivo* exposures [155].

Figgs et al. [201] found that the lymphocyte replicative index increased after spraying 2,4-D (*P* = 0.016), independent of tobacco and alcohol use, in a study involving two applicators spraying only 2,4-D. The data demonstrated a weak dose response with increasing urinary 2,4-D levels (*P* = 0.15). Lymphocyte immunologic phenotypes and complete blood counts (CBC) before spraying 2,4-D were not statistically different after spraying 2,4-D nor were there significant differences between 2,4-D applicators and controls after applicators had sprayed. The authors found no relationship between the frequency of micronuclei and urinary 2,4-D levels and conclude there are no human chromosome-damage outcomes at mean urinary 2,4-D levels ranging from 12 to 1285 ppb. Increased replicative index scores may be important because they suggest stimulated cell growth that could contribute to carcinogenesis. However, the finding of no relationship between the frequency of micronuclei and urinary 2,4-D level does not support a human chromosome-damage outcome at mean urinary 2,4-D levels ranging from 12 to 1285 ppb.

In a follow-on study to Figgs et al. [201], Holland et al. [21] evaluated cultured lymphocytes from the workers described above using a micronucleus assay and replicative index, a measure of cell division kinetics, as well as an associated in vitro study using whole blood and cultured lymphocytes to which a commercial formulation containing 2,4-D (Spurge and Oxalis Killer) as well as pure 2,4-D in different vehicles (e.g., ethanol, DMSO) was added. This study demonstrated that the lymphocytes of the 12 male applicators described above had a significantly higher replicative index than the same group prior to exposure and than a control group (*P* < 0.01). These results corroborate the *in vitro* finding in this study of increased replicative index at low doses (0.005 mM 2,4-D). *In vitro* there was a significant inhibition of lymphocyte proliferation for all five individuals at the highest dose level (0.3 mM) independent of the vehicle used for both pure and commercial 2,4-D (*P* < 0.001). At the low dose (0.005 mM) of commercial 2,4-D, four out of five study subjects exhibited an increase in replicative index. Pure 2,4-D results were inconclusive with three individuals responding with increased proliferation and four actually declining. This study showed a micronucleus increase above normal baseline only at high 2,4-D doses, that is, those approaching cytotoxic levels. The authors conclude that genotoxicity of 2,4-D as measured by the bone marrow micronucleus assay at environmentally relevant concentrations is negligible but find that increased proliferation after low 2,4-D exposure may be significant. Similarly, an extensive review of 2,4-D by the German Research Foundation [40, page 90] concluded there was sufficient evidence of a weak promoting effect of herbicide formulations of 2,4-D.

### 5.3. Summary of Toxicological Studies

Table 9 provides a brief summary of the studies that have explored genotoxicity and cytotoxicity both *in vivo* and *in vitro* in animals and humans. A negative sign indicates a negative result. A single plus indicates a positive result, but either only weakly positive (not statistically significant) or statistically significant but at very high concentrations relative to environmental exposures, including occupational exposures.

**Rodent Bioassays**

- Standard carcinogenic bioassays in rodents show no carcinogenic effects at concentrations ranging from 1 to 500 mg/kg-d.
  - US EPA (IRIS) RfD for 2,4-D is 0.01 mg/kg-d (http://www.epa.gov/iris/); the US EPA Office of Pesticide Programs uses 0.005 mg/kg-d in risk assessments conducted to support pesticide registration evaluations.
  - The published IRIS value is 0.0005 mg/kg-d; the US EPA Office of Pesticide Programs uses 0.0044 mg/kg-d in risk assessments conducted to support pesticide registration evaluations.

**Genotoxicity**

- Soloneski et al. [22] and Zeljezic and Garaj-Vrhovac [199] show that *in vitro* exposures at 4–10 *μ*g/mL 2,4-D and/or a commercial product containing 2,4-D are associated with statistically significant increases in SCE, but *in vivo* exposures in workers are more equivocal.
- Most *in vitro* studies in both human and animal cell cultures show effects at concentrations greater than would be expected in the environment.
- Genotoxicity was not observed across a battery of ToxCast assays.
- 2,4-D is negative for genotoxicity in bacterial mutation assays.
- Some positive results for mutagenicity have been observed in assays in yeast, plants, and insects.
- Negative results have been observed for mutagenicity across *in vivo* mammalian studies.
- Mixed results have been observed based on mammalian *in vitro* tests.
- Proliferative and immunological effects.
- 2,4-D and MCPA are weak peroxisome proliferators.
- 2,4-D and MCPA increase lymphocyte replicative index.
- Occupationally exposed individuals showed temporary increases in immunological markers.
- MCPA tested positive in six of over 500 ToxCast assays, and 2,4-D tested positive in eight.

## 6. Exposure and Biomonitoring

Although the dose makes the poison, it is the exposure that makes the dose, which is both a function of exposure concentrations in the environment and the relationship between exposed and absorbed dose. 2,4-D and MCPA are relatively straightforward to study, since they do not metabolize and are readily excreted in urine largely as parent compound within days of exposure. A number of models have been developed to explore and predict the relationship between exposures, particularly as defined in epidemiological studies (e.g., different spraying methods, occupational methods, uses, and durations for farmers versus lawn care professionals, etc.) and observed levels in urine as it is the characterization of exposure that is the greatest weakness of the epidemiological studies [228]. Developing models helps researchers to understand differences in exposures across methods of applications and assists in defining likely exposure routes for future studies. In this analysis, these data and models provide the context for the interpretation of potential cellular impacts as they relate to a potential mode of action for carcinogenic outcomes in humans.

### 6.1. Predictors of 2,4-D and MCPA Exposure in Occupational Settings and Observed Urinary Levels

Table 10 provides a summary of biomonitoring studies from the literature and the conditions under which these urinary levels were measured. Most of the studies are in occupational settings, but several include spouses and family members. For example, Arbuckle et al. [218] examined predictors of urinary 2,4-D levels among 126 farm applicators in the first 24 h after the first pesticide application of the season [218]. The variables pesticide formulation, protective clothing, application equipment, handling practice, and personal hygiene practice were found to explain 39% of the variability in 2,4-D dose. The mean and geometric mean urinary levels among 43 applicators reporting use of 2,4-D were 27.63 and 5.63 *μ*g/L, respectively.

A similar study conducted by Bhatti et al. [224] found much higher mean and geometric mean urinary levels, but this study followed noxious weed control applicators over a 12 week period (longer than the Arbuckle study). Overnight (approximately 12 h) urine samples were obtained from study participants every other week after a typical day of 2,4-D application. A total of 140 urine samples were collected (45 samples were collected in 1994 and 95 samples were collected in 1995). The best-fit multivariate model explained only approximately 23% of the variation in predicted urinary levels.

Harris et al. [229] found that volume of pesticide applied explained 20% of the variation in 2,4-D dose among 98 professional turf applicators over a 1 week period (mean and geometric mean daily dose of 2,4-D 1399 and 420 mg, resp.). Type of spray nozzle used, and the use of gloves while spraying explained an additional 43% of variation in 2,4-D dose [229]. In a study of 34 farm applicators and their families with urine samples collected 1 day before through 3 days after an application, glove use, repairing equipment, and number of acres treated were found to be the most significant predictors of 2,4-D concentration among applicators (geometric mean urinary 2,4-D concentration 1, 2, and 3 days after application was 33.4, 33.3, and 16.3 mg/g creatinine, resp.) [223].

In a study of Swedish forestry workers, Frank et al. [213] measured urine levels for six volunteer workers involved in mixing and loading 2,4-D ester solutions into aircraft and in guiding the spray aircraft in two conifer release programs during 1981 and 1982. The highest measured urinary level (22.2 *μ*g/kg body weight/day) was backcalculated to a maximum absorbed dose of 60 *μ*g/kg-d assuming an 18 hr half life for excretion of 2,4-D.

In a study involving 12 applicators spraying only 2,4-D, Figgs et al. [201] collected 45 urine specimens over time with concentrations ranging from 1.0 to 1,700 (lg 2,4-D/g creatinine/L urine) that increased logarithmically as spraying time increased. However, the relationship between urine concentrations and potential exposures was not provided or explored.

Lavy et al. [215, 230] evaluated potential exposures to US Forestry Service personnel occupationally exposed to 2,4-D under four different application regimes, including backpack spraying, injection bar, Hypohatchet, and hack-and-squirt. Four groups of 20 workers each were selected who had no known herbicide exposure for at least seven days before beginning the test. Each worker applied herbicide in a 12-d, two-part test, including a preapplication day, an application day on which usual application procedures were used followed by four days of no new exposures. The following week, the workers had another preapplication day, a second application day on which special precautions to minimize exposure were taken followed by four days of no new exposure. The total urine excreted each day was collected from each worker. The authors measured an average of backpack applicators applying 2,4-D during a 7 h day in T-1 had an absorbed dose of 0.088 mg/kg. The average absorbed doses of 2,4-D during T-1 for others applying Tordon 101-R were 0.010, 0.085, and 0.029 mg/kg for the injection bar, Hypohatchet and hack-and-squirt crews, respectively.

GM urinary 2,4-D levels for broadcast spray applicators in Thomas et al. [225] (GM 21 *μ*g/L; range 2.5–270 *μ*g/L for day 1 urine samples) were lower than those measured by Acquavella et al. [222] (GM 64 *μ*g/L; range 2–1856 mg/L), but higher than those reported by Arbuckle et al. [218] (GM 5.4 *μ*g/L; range 0.5–410 *μ*g/L) for 43 Ontario farm applicators.

Durkin et al. [231] developed a physiologically based pharmacokinetic (PBPK) model of 2,4-D in humans based on an unpublished study by Dow Chemical involving rats. They then calibrated the model using human data from Sauerhoff et al. [25] and Feldmann and Maibach [232]. The model considers flow-limited pH trapping modified to consider tissue binding, binding to plasma, and high-dose inhibition of urinary excretion in tissue, skin, GI tract, kidney, liver, and blood. Exposure is primarily through dermal contact. Lavy et al. [215, 230] measured exposures to backpack applicators in a study for the US Forest Service, and these data were used in the model to determine disposition of 2,4-D under typical exposure conditions. 

Thomas et al. [225] monitored private pesticide applicators in the Agricultural Health Study (AHS), epidemiological cohort was monitored around the time of their agricultural use of 2,4-D and obtained urinary samples as well as patch, hand-wipe, and personal air samples. Preapplication urinary levels averaged approximately 8 *μ*g/L, which increased to an average of 25 *μ*g/L following several days of 2,4-D application.

In an observational research study of 135 preschool children and their caregivers in NC and OH, Morgan et al.'s report measured urinary levels based on several spot samples [226]. The highest measured sample in a child was 12.5 *μ*g/L, translating to a dose of 0.28 mg/kg-d assuming a daily urine excretion of 22.4 mL/kg bw for children [226], a value 35 times lower than the IRIS RfD of 0.01 mg/kg-d.

Aylward et al. [26] report the 50th and 95th percentiles from the National Health and Nutrition Examination Survey (NHANES) dataset, which shows levels in the general public based on several spot samples that are comparable to Morgan et al. [226].

### 6.2. Exposure Pathways

The available data suggest that the primary exposure pathway for residential and nonoccupational exposures is dermal exposure following application (e.g., treatment of yards, etc.) followed by oral exposure and that inhalation exposures are negligible [146, 147, 217] representing less than 0.2% of overall exposures in occupationally exposed adults [38, 231]. The highest inhalation exposures have been documented for workers in production facilities [154]; even sprayers do not experience significant inhalation exposures [38, 231]. Consequently, the primary exposure to 2,4-D and MCPA in the environment is dermal and, to a lesser extent, oral ingestion. Studies summarized in Ross et al. [145] show that less than 10% of dermally applied 2,4-D is absorbed in occupationally exposed adults.

In risk assessments developed to support reregistration of MCPA in Canada, Health Canada [147] estimates the contribution of inhalation exposure to the overall exposure in postapplication scenarios as negligible, due to the dilution effect of outdoor use and considering the study by Yeary and Leonard [233], wherein MCPA was not detected in the breathing zone of 25 applicators during the application of MCPA to residential lawns, trees, and shrubs (limit of detection of 0.001 mg/m^3^). Similarly, inhalation has been shown to contribute less than 2% of the cumulative exposure among 2,4-D applicators [234]. Further, air concentrations of up to 20 mg/m^3^ did not correspond with measurable exposure in any of the bystanders to a 2,4-D spray application [217].

A series of studies by Nishioka et al. [235–237] evaluating transport of lawn-applied 2,4-D into homes, including measurements of how much was tracked relative to how much was applied, and the primary tracking mechanisms found low but measurable concentrations of 2,4-D inside homes and conclude that although low, these concentrations could lead to dermal and oral (but not inhalation) exposures. Similarly, Mustonen et al. [193] measured air breathing space of workers and found very low air concentrations from spraying concurrent with measured urinary concentrations in 19 workers and conclude that dermal exposures represent the primary source of exposures. 

The exception to the dermal pathway as the dominant exposure pathway is for children. Wilson et al. [238] in a study of children exposed to residential use of pesticides in North Carolina and Ohio found that the diet represented approximately 80–90% of the daily dose of 2,4-D for children. Inhalation was 3-4% and dermal 9–15%. Based on observed urinary levels in 287 children, the aggregate potential dose was approximately 9-10 ng/kd-d (for reference, the IRIS RfD is 0.01 mg/kg-day or 10^4^ ng/kd-d). The maximum predicted aggregate dose ranged from 98 to 177 ng/kg-d.

### 6.3. Biomonitoring Equivalents

Biomonitoring equivalents are urine and/or blood concentrations associated with exposures in humans to chemical-specific regulatory standards such as the RfD.

Aylward et al. [26] reviewed the available biomonitoring data for 2,4-D from the United States and Canada and compared these data with expected biomonitoring equivalents based on regulatory threshold values to draw conclusions regarding the margin of safety for 2,4-D exposures based on published biomonitoring data for the general population, farm applicators, and farm family members. Aylward and Hayes [155] estimated a biomonitoring equivalent in urine of 200 *μ*g/L (or 300 *μ*g/g creatinine) associated with chronic, low-level exposure to 0.005 mg/kg-d. The analysis reflects oral exposures only; that is, 200 *μ*g/L in urine is the concentration associated with a daily, steady-state oral exposure of 0.005 mg/kg-d based on the following equation:

(1) $$\begin{array}{c} \text{Urinary Level}=\frac{\text{Dose}*\text{BW}}{V_{24\;\text{hr}}}, \end{array}$$

where Urinary level = volume-based urinary level in *μ*g/L, Dose = Dose (RfD or from (2)), BW = body weight, *V*_24 hr_ = volume of urine in 24 hrs in L.

Given the potentially relevant positive results from the suite of 500 ToxCast assays described previously, these *in vitro* results were explored in the context of *in vivo* exposures using the following methodology. First, oral equivalent doses associated with the lowest biologically relevant *in vitro* ToxCast assay results (Table 5) are estimated based on the following equation [172, 239]:

(2) $$\begin{array}{c} \text{Dose}=\text{Assay}*\frac{1\left(\text{mg}\;/\text{kg}\right)-d}{C_{\text{ss}}}, \end{array}$$

where Dose = oral equivalent dose in mg/kg-d, Assay = lowest effective concentration for a biologically relevant pathway from the ToxCast assay in *μ*M, and *C*_ss_ = steady-state concentration from PBPK model assuming 1 mg/kg-d oral exposure [172, 231].

**Figure 7:**
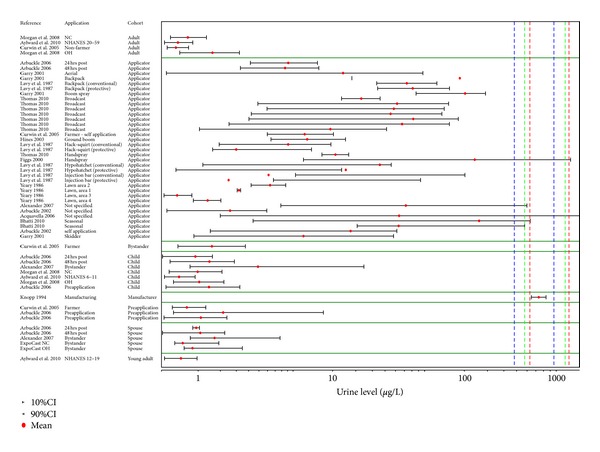
Biomonitoring data for 2,4-D in the context of backcalculated urine levels. Reference lines: green = men 575 *μ*g/L (RfD), men 1170 *μ*g/L (ToxCast reverse dosimetry). Blue = women 480 *μ*g/L (RfD), women 960 *μ*g/L (ToxCast reverse dosimetry). Red = child 630 *μ*g/L (RfD), child 1250 *μ*g/L (ToxCast reverse dosimetry) (see for review [26, 124, 154, 201, 214, 215, 218–227]).

The resulting predicted urinary level is based on the relationship provided in Aylward et al. [155] and shown in (1).  Table 11 shows the results for children 4–12, adolescents up to 18, men, and women including input assumptions for each, and Figure 7 provides a graphical depiction of these results in the context of the biomonitoring data. 

**Figure 8:**
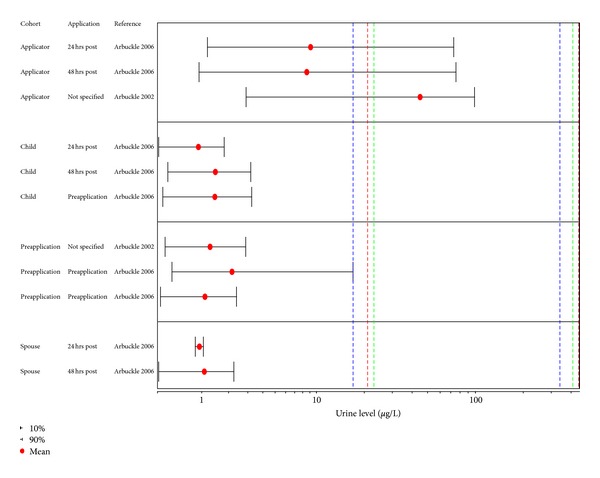
Biomonitoring data for MCPA in the context of backcalculated urine levels. Reference lines: green = men 23 *μ*g/L (RfD), men 410 *μ*g/L (ToxCast reverse dosimetry). Blue = women 17 *μ*g/L (RfD), women 340 *μ*g/L (ToxCast reverse dosimetry). Red = child 21 *μ*g/L (RfD), child 450 *μ*g/L (ToxCast reverse dosimetry) see for review [218, 221].

The highest predicted steady-state concentration for 2,4-D from the PBPK models is approximately 90 *μ*M, and the lowest biologically relevant ToxCast assay result is 1.5 *μ*M based on cell growth kinetics., The associated estimated urinary levels are provided in the last column of Table 11 based on (1). The average *C*_ss_ (steady-state body burden associated with 1 mg/kg exposure) predicted by Wetmore et al. [172] is approximately 40 *μ*M. The resulting ranges of predicted 2,4-D urinary levels associated with the lowest observed response from the *in vitro* ToxCast assays is 600–1250 *μ*g/L for children, 440–900 *μ*g/L for adolescents, 470–960 *μ*g/L for women, and 560–1200 *μ*g/L for men, see Figure 8. These compare to the biomonitoring equivalents developed by Aylward and Hays [26] and Aylward [155] of 200 *μ*g/L for an adult population based on a urinary level associated with exposure to the RfD. 

By parameterizing lognormal distributions using the parameters in Table 10 (geometric mean and geometric standard deviation, in most cases), 10th and 90th percentiles for each distribution were developed using the Crystal Ball Excel add-in, and these are the basis of the whiskers in Figures 7 and 8. Comparing the backcalculated urine levels to the biomonitoring data from Table 10 shows that the only overlap between the levels associated with the potential for *in vitro* effects (at the lowest biologically relevant assay result) and data from biomonitoring studies is for one study in manufacturing workers [154]. The remaining studies show that even the predicted 90th percentiles fall well below these backcalculated levels with only a few exceptions for applicators. Most values even fall below the 200 *μ*g/L level based on the RfD backcalculation. The data suggest some transient occupational exposures that may come close to overlapping the backcalculated assay results, but the use of protective gear would preclude these exposures from occurring.

There is less data available for MCPA, but based on the results presented in Wetmore et al. [172] combined with (1) [155] shows that backcalculated urinary levels are approximately 450 *μ*g/L for children, 320 *μ*g/L for adolescents, 240 *μ*g/L for women, and 310 *μ*g/L for men as shown in the bottom portion of Table 11. Using the published IRIS RfD of 0.0005 mg/kg-d results in predicted urinary levels an order of magnitude lower, and the regulatory value used by Health Canada and US EPA Office of Pesticide Programs (0.0044 mg/kg-d) falls in-between. There are no direct MCPA biomonitoring data available, but Figure 8 shows these backcalculated values in the context of the Arbuckle et al. [218] study (for which 2,4-D and MCPA urinary levels coeluted). The backcalculated bioassay results show no overlap, but the backcalculated level from the RfD falls within the biomonitoring data for the applicators. However, there is no particular relevance of the RfD with respect to potential carcinogenicity.

### 6.4. Summary of Exposure and Biomonitoring

Dermal absorption represents the primary exposure route in both occupationally exposed individuals and the general public, followed by ingestion, while inhalation exposures are negligible in residential settings and largely negligible even in occupational settings.

- There are numerous studies available for characterizing 2,4-D and MCPA concentrations in homes following residential application of 2,4-D.
- Occupational exposures depend heavily on the amount of protective clothing that is worn and vary widely; exposures at the 95th percentile in the general public are typically less than 100 times the IRIS RfD of 0.01 mg/kg-d.
- Backcalculated urinary levels using the results from the lowest observed bioassay result required to alter a relevant biological pathway *in vitro* are an order of magnitude higher than levels based on the RfD for MCPA and a factor of five higher for 2,4-D.
- There are orders of magnitude difference between estimated urine levels equivalent to the lowest ToxCast concentrations required to alter biologically relevant pathways and biomonitoring data. The difference is less for occupational exposures, but still generally large, even at the 90th percentile.

## 7. Discussion and Conclusions

Chlorophenoxy compounds have been in use since the 1940s, and despite numerous regulatory and nonregulatory reviews, they continue to be controversial, particularly with respect to carcinogenic outcomes. Early epidemiologic studies that defined exposures in terms of job matrices rather than through quantitative estimates of actual exposures to chlorophenoxy compounds found some associations with various lymphomas, particularly NHL. The Swedish studies, in particular, [71] found significant associations between exposure specifically to MCPA, NHL, and STS [90], although those associations were not confirmed in other studies. Associations were limited to case-control studies with small sample sizes that were not confirmed by the cohort studies. Potential associations in case-control studies were based on univariate analyses without including other potential exposures and/or known risk factors, while those studies incorporating the variety of exposures experienced in the environment generally show no statistically significant role for exposures to chlorophenoxy compounds. More recent epidemiologic studies linking genetic markers of effect (e.g., t(14;18) translocations) find no association with exposure to chlorophenoxy compounds and carcinogenic outcomes. Genomic instability observed in agricultural workers has not been associated with exposure to 2,4-D, and in any event have been shown to be transient and reversible [124]. Short-term immunosuppressive effects have been observed in humans [200] following exposure to 2,4-D and MCPA, and although most of these effects were transient, some were still observed 70 days after exposure. Given that exposures to 2,4-D and MCPA are episodic in nature, the question arises as to what role repeated short-term immunosuppression might play in contributing to an increased risk of developing NHL.

Toxicological studies conducted *in vivo* using traditional rodent assays over one or two years showed no treatment-related carcinogenic effects although other effects were noted, particularly to the kidney and liver. Animal studies *in vitro* are equivocal, with some suggesting that 2,4-D and/or MCPA are able to cause chromosomal aberrations and interrupt key cellular functions while others do not (summarized in Table 9), but generally showing effects at concentrations exceeding renal transport mechanisms. Studies involving human cell cultures or human cells derived from *in vivo* exposures do suggest that 2,4-D and/or MCPA are capable of causing chromosomal aberrations in some studies [186, 188, 191, 212] but not in others [19, 181], and both 2,4-D and MCPA showed negative results across a battery of ToxCat genotoxicity assays [211]. Interestingly, some positive associations are noted for exposure to a commercial product containing 2,4-D but not for the pure 2,4-D acid or salt [21, 193]. 

Studies are quite consistent; however, in suggesting that exposure to 2,4-D can disrupt other cellular functions and lead to cell replication necessary for tumor promotion *in vivo*, but only after longer exposure periods and only at the highest concentrations tested. Observed 2,4-D toxicity generally occurs at doses above renal saturation; that is, doses above which excretory processes could readily eliminate the chemical. Nonetheless, an extensive review of 2,4-D by the German Research Foundation [40, page 90] concluded there was sufficient evidence of a weak promoting effect of herbicide formulations of 2,4-D. Studies in human volunteers have shown that while immunological effects following exposure were observed [200], effects only persisted during exposure. Although effects showed a relationship to urine levels of 2,4-D, the trend in the relationship was not statistically significant. Thus, the combined evidence indicates that it is only at exposures exceeding renal transport mechanisms that effects are observed. What is less well understood is whether exposures at lower concentrations but over longer periods of time might be sufficient to effect relevant cellular changes. The episodic nature of environmental exposures, however, suggests this is unlikely to occur. 

The etiology of NHL suggests a multistage process including specific chromosomal translocations present in nearly 90% of all cases coupled with proliferating events such as interruption of apoptosis, increased chronic inflammation as a result of oxidative stress or peroxisome proliferation, increased production of free radicals, or direct proliferation of a mutation. The evidence does not support an association between exposures to 2,4-D and/or MCPA and direct DNA interaction; however, the specific chromosomal translocations observed in NHL (e.g., t(14;18) are prevalent in healthy individuals [63]; therefore, it is not unreasonable to assume that a significant proportion of individuals exposed to 2,4-D and/or MCPA already have the required translocations.

NHL most often arises in premature and/or naïve B cells, and a host of genetic markers has been identified with respect to key subclinical features of the disease. Epidemiologic studies are incorporating these genetic markers, and these studies show no association between exposure to chlorophenoxy compounds broadly defined (of which 2,4-D is likely to be a predominant exposure) and these markers. Although the toxicological data in human cell cultures and/or *in vivo* human exposures suggest that there are plausible mechanisms by which exposure to 2,4-D could promote NHL by causing cellular proliferation as evidenced by positive responses in replicative index assays, the doses at which these effects are noted are well above levels observed even in occupational settings (based on measured urine) and are likely to exceed saturation of renal transport mechanisms. 

Exposure data and measured urine levels in workers and in the general public show that exposures to 2,4-D and MCPA in the environment are at levels below RfDs published in IRIS or by the Office of Pesticide Programs. Modeled urine levels based on chronic exposures to the RfD for 2,4-D, when compared to biomonitoring data, show that urine levels, even in occupationally exposed individuals, are below levels of concern. Exposures in the environment are predominantly through dermal absorption, and studies show less than 10% of 2,4-D and MCPA are dermally absorbed. Studies that have estimated daily doses to the general public based on repeated measurements in the home show these doses are orders of magnitude below the IRIS RfDs.

The only plausible potential for risk that can be hypothesized would be in an occupationally exposed subpopulation with specific polymorphisms, family history, and/or lifestyle characteristics identified as risk factors for NHL, but that assumes that occupational exposures would be high enough and sustained enough to lead to adverse effects. Figures 7 and 8 shows the difference between measured urine levels and estimated urine levels from exposures at the RfD or exposures at the lowest biologically relevant ToxCast assay results. As seen in those figures, the difference is significant at the 90th percentile across all but a few occupational studies. This difference translates to several orders of magnitude for the general public, and at least an order of magnitude, in general, for occupational exposures. Given that the RfD is a dose associated with no effects (including a margin of safety), and that *in vitro* bioassay results may not translate to *in vivo* effects, the combined evidence indicates it is highly implausible that exposures to 2,4-D and/or MCPA are associated with a risk of developing NHL or other lymphohematopoietic cancers.

However, exposures and potential impacts are considered only for exposure to 2,4-D and MCPA in isolation. From a cumulative risk perspective, there are likely numerous concurrent exposures, some of which exert similar immunosuppressive or proliferative effects, and the combined impact of these exposures has not been considered. That represents a more complex question that cannot be addressed by the data in-hand since so much of that is unique to the individual, but there is an opportunity going forward for epidemiologic and other studies to evaluate these combined exposures more comprehensively. That said, given the difference between observed exposures as measured by urine levels and estimated urine levels from regulatory or ToxCast values indicates that environmental exposures to 2,4-D and/or MCPA are unlikely to be risk drivers even in a cumulative risk context.

## Supplementary Material

Supplementary materials provide the genetic analysis profile (GAP) developed by IARC to evaluate mutagenicity of 2,4-D [10, 29, 45].

## Figures and Tables

**Table 1:** Summary of reviews of 2,4-D and/or MCPA.

Reference	Evaluation	Conclusions
Regulatory reviews or reviews in support of regulatory activities		

US EPA, SAB [27]	Science Advisory Board consultation on carcinogenicity of 2,4-D	“Data are not sufficient to conclude that there is a cause and effect relationship between exposure to 2,4-D and non-Hodgkin's lymphoma.”

US EPA [28]	4th carcinogenicity of 2,4-D peer review	Not classifiable as to carcinogenicity.

WHO/IARC [29]	Evaluations of carcinogenic risk	Inadequate and/or limited for 2,4-D specifically and chlorophenoxy compounds generally.

US EPA [5]	Health Effects Division Carcinogenicity Peer Review Committee (2,4-D)	“Evidence is inadequate and cannot be interpreted as showing either the presence or absence of a carcinogenic effect.”

European Commission [30]	Review report for 2,4-D	Proposed uses have no harmful effects on animal or human health; no evidence of carcinogenicity.

US EPA [3, 4]	Risk assessments and reregistration decision for MCPA	Limited evidence for carcinogenicity.

US EPA [8]	Risk assessments and reregistration decision for 2,4-D	Group D, not classifiable as to carcinogenicity.

Health Canada PMRA [31]	Reregistration decision for 2,4-D	No evidence of carcinogenicity.

Health Canada PMRA [32]	Reregistration decision for MCPA	No evidence of carcinogenicity.

Health Canada [9]	MCPA in drinking water	Not considered a carcinogen.

77FR23135 [33]	Response to NRDC petition to revoke 2,4-D registration	No new evidence that would suggest registration should be revoked.

Nonregulatory reviews		

Canadian Centre for Toxicology [34]	Expert panel on carcinogenicity of 2,4-D	No evidence that 2,4-D forms reactive intermediates in the liver or other tissues or forms adducts with DNA. Existing animal and human data are insufficient to support the finding that 2,4-D is a carcinogen; insufficient evidence that existing uses of 2,4-D pose a significant human health risk.

Kelly and Guidotti [35]	Review of literature to advise provincial regulatory body on chlorophenoxy safety, particulary 2,4-D	Evidence for a causal association is strongest for NHL and probably reflects either a weak effect or, possibly, a confounding exposure associated with the use of 2,4-D. Given worst-case assumptions, potency of 2,4-D as a carcinogen is probably weak. Its intrinsic toxicity is less than that of alternative herbicides.

Johnson [36, 36]	13 cohort studies (9 cohorts) in chlorophenoxy manufacturing; 16 cohort studies (12 cohorts) in chlorophenoxy spraying	The weight of evidence does not unequivocally support an association between use of chlorophenoxys and malignant lymphomas/STS; occupational cohort studies have not accumulated sufficient person-years of observation to date, yet see cases of myeloid lymphoma and STS when none are expected.

Ibrahim et al. [37]	Human carcinogenicity of 2,4-D	The toxicological data alone do not provide a strong basis for determination of carcinogenicity of 2,4-D. Suggestive although inconclusive evidence for an association between exposure to 2,4-D and NHL based on epi studies and further study was warranted. Little evidence of an association between 2,4-D and STS or HD, and no evidence of an association between 2,4-D use and any other form of cancer.

Munroe et al. [38]	Comprehensive, integrated review of 2,4-D safety in humans	No evidence for adverse health effects; no mechanistic basis by which 2,4-D could cause cancer.

Morrison et al. [14]	Meta analysis of epidemiological studies involving occupational exposures to chlorophenoxy compounds	Suggestive evidence of an association with NHL; no evidence for an association with STS, HD, or leukemia.

Bond and Rossbacher [39]	Potential carcinogenicity of MCPA, MCPP, 2,4-DP	No evidence for carcinogenicity across all three compounds.

Henschler and Greim [40]	Comprehensive, integrated review to establish “maximale arbeitsplatz konzentration”	Evidence for potential proliferative response; less evidence for genotoxicity.

Gandhi et al. [41]	Potential carcinogenicity of 2,4-D	Some suggestive evidence for an association with NHL but without any plausible mode of action.

Garabrant and Philbert [42]	Comprehensive, integrated review of potential for health effects in humans	No causal association of any form of cancer with 2,4-D exposure. Animal studies of acute, subchronic, and chronic exposure to 2,4-D, its salts, and esters showed an unequivocal lack of systemic toxicity at doses that did not exceed renal clearance mechanisms. No evidence that 2,4-D in any of its forms activated or transformed the immune system in animals at any dose.

Bus and Hammond [43]	Update on data generated by the Industry Task Force II on 2,4-D Research Data	Toxicity responses limited to highest doses; not a carcinogen or genotoxicant, does not cause birth defects; low potential for reproductive toxicity and neurotoxicity.

Van Maele-Fabry et al. [44]	Cohort studies for chlorophenoxy expousures and leukemia	Meta-analysis of 3 cohort studies; statistically significant odds ratio = 1.60, 95% confidence interval = 1.02–2.52; all three underlying studies individually showed non-significant associations.

**Table 2:** Case-control studies of chlorophenoxy compounds and NHL.

NHL OR	Cases	Controls	Reference	Herbicide	Notes
**4.8 (2.9–8.1)**	41	24	Hardell et al. [72]	2,4,5-T; 2,4-D; MCPA	Chlorophenols, solvents, and other pesticides. Exposure defined as greater than one day; entirely self-reported. One documented MCPA exposure across cases; results for NHL and HD combined. Forestry.

**4.9 (1.3–18)**	6	6	Persson et al. [68]	Phenoxys	Chlorophenols, solvents, and other pesticides. Variety of occupations and exposure described by occupation. Exposure to wood preservatives/creosote predicted much higher ORs; exposure to pets comparable. Only logistic model statistically significant.

**13 (1.2–360)**	3	1	Hardell et al. [56]	2,4-D only	Chlorophenols. Potential for recall bias in questionnaires used for exposure. No dose response relationship observed. Forestry.

**5.5 (2.7–11)**	25	24	Hardell et al. [56]	Phenoxys (including 2,4-D, 2,4,5-T, and MCPA)	2,4,5-T known to contain dioxin. No dose response relationship observed.

**2.7 (1.0–7.0)**	12	11	Hardell and Eriksson [60]	Primarily MCPA	Greater than 30 year latency to achieve statistical significance.
1.3 (0.7–2.3)	43	62	Hardell and Eriksson [60]	2,4-D/2,4,5-T	

**2.81 (1.27–6.22)**	21	9	Eriksson et al. [71]	MCPA only overall	Questionnaire on work history; questions on exposure to pesticides, organic solvents, and other chemicals. Numbers of years, days per year, and approximate length of exposure per day. No job-exposure matrix could be developed. One full day of exposure constituted exposure.
**3.76 (1.35–10.5)**	15	5	Eriksson et al. [71]	MCPA < 32 days of use	
1.66 (0.46–5.96)	6	4	Eriksson et al. [71]	MCPA > 32 days of use	
**2.04 (1.24–3.36)**	47	26	Eriksson et al. [71]	Phenoxys overall	
**2.83 (1.47–5.47)**	32	13	Eriksson et al. [71]	Phenoxys < 45 days of use	
1.27 (0.59–2.70)	15	13	Eriksson et al. [71]	Phenoxys > 45 days of use	

1.3 (0.8–2.1)	51	92	Pearce et al. [73]	Primarily 2,4,5-T; MCPA not mentioned	Fencing work; employment in meat works statistically significant. “Farming” assumed to represent chlorophenoxy exposures.

**2.2 (1.2–4.1)**	24	78	Hoar et al. [57]	2,4-D (overall, not stratified)	No dose response (see below).

2.7 (0.9–8.1)	6	17	Hoar et al. [57]	Use of 2,4-D 1-2 d/y (frequency)	Only overall analysis statistically significant; no dose response relationship. Many other exposures; use of hierarchical modeling by class (herbicides only, insecticides only, etc.). Detailed questionnaire; but elicited dates and frequency of use of any herbicide on each farm instead of dates and frequency for each specific herbicide.
1.6 (0.4–5.7)	4	16	Hoar et al. [57]	Use of 2,4-D 3–5 d/y (frequency)	
1.9 (0.5–6.7)	4	16	Hoar et al. [57]	Use of 2,4-D 6–10 d/y (frequency)	

3.0 (0.7–11.8)	4	9	Hoar et al. [57]	Use of 2,4-D 11–20 d/y (frequency)	
**7.6 (1.8–32.3)**	5	6	Hoar et al. [57]	Use of 2,4-D > 21 d/y	Only overall analysis statistically significant; no dose response relationship. Many other exposures; use of hierarchical modeling by class (herbicides only, insecticides only, etc.). Detailed questionnaire; but elicited dates and frequency of use of any herbicide on each farm instead of dates and frequency for each specific herbicide.
1.3 (0.3–5.1)	3	16	Hoar et al. [57]	Use of 2,4-D 1–5 yrs (duration)	
2.5 (0.9–6.8)	7	22	Hoar et al. [57]	Use of 2,4-D 6–15 yrs (duration)	
**3.9 (1.4–10.9)**	8	15	Hoar et al. [57]	Use of 2,4-D 16–25 yrs (duration)	
2.3 (0.7–6.8)	6	17	Hoar et al. [57]	Use of 2,4-D > 26 yr (duration)	

1.5 (0.9–2.5)	43	98	Zahm et al. [61]	2,4-D	Lawn care professionals.

1.07 (0.8–1.4)	NR	NR	Woods et al. [66]	Phenoxys; other compounds	Increased risks found for DDT (1.82 (1.04-3.2)) and organic solvents (1.35 (1.06-1.7)). Exposure based on job description. No dose response observed for phenoxys.

0.68 (0.3–1.4)	181	196	Woods [65]	2,4-D	Small but significant excess risk observed for farmers (all chemicals). Exposure poorly defined.
0.71 (0.3–1.5)	181	196	Woods [65]	Any phenoxy	

1.2 (0.9–1.6)	118	231	Cantor et al. [64]	Largely 2,4-D	Statistically significant OR > 1.5 for personal handling, mixing, or application of carbaryl, chlordane, dichlorodiphenyltrichloroethane, diazinon, dichlorvos, lindane, maialinoli, nicotine, and toxaphene.

0.5 (0.2–1.2)	6	79	Leiss and Savitz [69]	Yard treated in last three months of pregnancy	Exposure dichotomous never exposed versus ever exposed; actual products and durations not specified. Lymphomas considered as a category.
0.8 (0.3–1.8)	15	118	Leiss and Savitz [69]	Yard treated in the two years between birth and diagnosis	

0.6 (0.4–1.0)	10	98	Leiss and Savitz [69]	Yard treated in the two years prior to diagnosis	Exposure dichotomous never exposed versus ever exposed; actual products and durations not specified. Lymphomas considered as a category.

1.25 (0.54–2.90)	19	85	Kogevinas et al. [59]	Phenoxys	Exposure evaluated for phenoxy herbicides and chlorophenols, polychlorinated dibenzodioxins and furans, raw materials, process chemicals, and chemicals used in the production of phenoxy herbicides.
0.88 (0.36–2.18)	15	76	Kogevinas et al. [59]	MCPA/MCPP	Three industrial hygienists carried out questionnaire-based analysis of exposure to 21 chemicals or mixtures. Nested case-control in IARC cohort [83].

1.11 (0.46–2.65)	12	56	Kogevinas et al. [59]	2,4-D/P/B	See previous/above

**1.32 (1.01–1.73)**	111	293	McDuffie et al. [13]	2,4-D	Small validation study of exposure questionnaire. Considered many covariates and exposures. Final models did not include MCPA or 2,4-D. Previous cancer and family history strong predictors.
1.10 (0.6–2.0)	17	46	McDuffie et al. [13]	MCPA	

1.25 (0.96–1.62)	110	293	McDuffie et al. [62]	2,4-D	Cross-Canada study. Mecoprop exposures show strongest relationship; also DEET.
1.09 (0.6–1.98)	17	46	McDuffie et al. [62]	MCPA	

1.09 (0.76–1.56)	64	186	McDuffie et al. [62]	2,4-D	Farm study. Mecoprop exposures show strongest relationship; also DEET.
0.98 (0.7–1.37)	15	33	McDuffie et al. [62]	MCPA	

0.9 (0.6–1.2)	123	314	De Roos et al. [16]	2,4-D	Pooled data from three previous case-control studies.
0.9 (0.4–2.0)	8	16	De Roos et al. [16]	MCPA	Strongest associations for organophosphates, chlordane, and dieldrin.

1.0 (0.5–2.0)	18	NR	Miligi et al. [58]	Chlorophenoxys and men	
1.3 (0.5–3.7)	11	NR	Miligi et al. [58]	Chlorophenoxys and women	Crop exposure matrix for exposure based on face-to-face interview questionnaire together with pesticide usage statistics by area.
0.7 (0.3–1.9)	6	NR	Miligi et al. [58]	2,4-D and men	
1.5 (0.4–5.7)	7	NR	Miligi et al. [58]	2,4-D and women	
1.1 (0.4–3.5)	8	NR	Miligi et al. [58]	MCPA and women	
1.1 (0.6–1.8)	32	28	Miligi et al. [58]	Chlorophenoxys overall	
2.4 (0.9–7.6)	13	6	Miligi et al. [58]	Chlorophenoxys without protective equipment	
0.9 (0.5–1.8)	17	18	Miligi et al. [87]	2,4-D overall	

**4.4 (1.1–29.1)**	9	3	Miligi et al. [87]	2,4-D without protective equipment	
0.9 (0.4–1.8)	18	19	Miligi et al. [87]	MCPA overall	Crop exposure matrix for exposure based on face-to-face interview questionnaire together with pesticide usage statistics by area.
3.4 (0.8–23.2)	7	3	Miligi et al. [87]	MCPA without protective equipment	

1.1 (0.78–1.55)	257	161	Hartge et al. [15]	500 ng/g 2,4-D in carpet dust	
0.91 (0.45–1.45)	86	59	Hartge et al. [15]	500–999 ng/g 2,4-D in carpet dust	No dose response observed; residential exposures from tracking following yard application. Measured exposures but not concurrent.
0.66 (0.45–0.98)	165	162	Hartge et al. [15]	1000–9999 ng/g 2,4-D in carpet dust	

0.82 (0.41–1.66)	24	18	Hartge et al. [15]	>10000 ng/g 2,4-D in carpet dust	See previous/above.

**3.8 (1.85–7.81)**	60	NR	Mills et al. [12]	2,4-D	US farmworkers union.

1.75 (0.42–7.38)	3	5	Fritschi et al. [67]	Chlorophenoxys	

0.9 (0.4–1.9)	11	25	Orsi et al. [70]	Phenoxys	

0.94 (0.67–1.33)	49	187	Hohenadel et al. [17]	2,4-D	

**1.78 (1.27–2.5)**	63	133	Hohenadel et al. [17]	2+ phenoxys	

**Table 3:** Cohort studies.

Reference	Metric	HD	STS	NHL	Leukemia	Exposure
Lynge [107]	SIR	Lymphoma = 1.1	2.72	Lymphoma = 1.1	1.3	Chlorophenoxy manufacturing workers; confidence intervals not provided

Wiklund et al. [96, 108, 109]	SIR	NR	0.9 (0.8–1.0)	NR	NR	MCPA, but including TCDD and other chlorophenoxy exposures

Wiklund et al. [76]	SIR	1.2 (0.6–2.16)	NR	1.01 (0.63–1.54)	NR	Chlorophenoxy, but including TCDD and other exposures

Wiklund et al. [77]	SIR	1.02 (0.88–1.88)	NR	0.97 (0.89–1.06)	NR	Chlorophenoxy, but including TCDD and other exposures

Bond et al. [110]	SMR	All lymphomas 2.02 (0.06–4.6)			2.2 (0.03–7.9)	2,4-D manufacturing

Wiklund et al. [78]	SIR	1.2 (0.6–2.16)	0.94 (0.34–2.04)	1.01 (0.63–1.54)	0.66 (0.28–1.21)	MCPA dominates but many other compounds also

Corrao et al. [111]	SIR	**1.4 (1.0–1.9) **(all lymphomas, including NHL, HD)			1.1 (0.8–1.5)	Assumed chlorophenoxy

Wigle et al. [80]	SMR	NR	0.89 (0.53–1.40)	0.92 (0.75–1.11)	0.88 (0.74–1.04)	Chlorophenoxy

Coggon et al. [81]	SMR	0 (0.–9.76)	0 (0–20.58)	2.72 (0.33–9.73)	NR	Chlorophenoxy

Bloemen et al. [112]	SIR	NR	NR	2.0 (0.2–7.1)	NR	2,4-D manufacturing; update to Bond [110]

Asp et al. [82] (10 yr latency)	SIR	1.18 (0.03–6.56)	None observed	0.42 (0.01–2.35)	1.23 (0.25–3.59)	Chlorophenoxy sprayers

Kogevinas et al. [83]	SMR	0.99 (0.48–1.82)	2.00 (0.91–3.79)	1.27 (0.88–1.78)	1.00 (0.69–1.39)	Chlorophenoxy and chlorophenols

Zahm [74]	SMR	NR	NR	1.14 (0.31–2.91)	NR	Lawn applicators; 4 deaths

Lynge [86]	SIR	NR	1.62 (0.4–4.1)	1.10 (0.4–2.6)	1.21 (0.49–2.50)	Mostly MCPA, MCPP, some 2,4-D, and 2,4,5-T

Burns et al. [79]	SMR	1.54 (0.04–8.56)	NR	1.00 (0.21–2.92)	1.30 (0.35–3.32)	2,4-D manufacturing; Update to Bond [110]; Bloemen [112]

'T Mannetje et al [84]	SMR	0 (0.–16.1)	4.28 (0.11–23.8)	0.69 (0.02–3.84)	1.16 (0.03–6.44)	Chlorophenoxy sprayers, include dioxin, paraquat, and organophosphates

'T Mannetje et al [84]	SMR	5.58 (0.14–31.0)	0 (0–19.3)	0.87 (0.02–4.87)	0 (0–5.29)	Production workers

Jones et al. [89]	SMR	1.15 (0.74–1.78)	0.96 (0.62–1.49)	**2.01 (1.38–2.93)**	1.02 (0.707–1.463)	Chlorophenoxy manufacturing, but many with predominantly 2,4,5-T known to be contaminated with dioxin; meta-analysis of 20 studies

Boers [85] Factory A	SMR	NR	NR	0.92 (0.19–4.47)	0.28 (0.03–2.61)	Chlorophenoxy, solvents

Boers [85] Factory B	SMR	NR	NR	only one case	1.53 (0.22–10.82)	MCPP, MCPA, and 2,4-D

Burns et al. [79]	SIR	0.97 (0.01–5.4)	0.6 (0.01–3.3)	1.2 (0.7–2.1)	0.9 (0.4–1.8)	2,4-D manufacturing; 1985–2007 cohort 1

Burns et al. [79]	SIR	1.05 (0.01–5.9)	0.7 (0.01–3.6)	1.4 (0.7–2.3)	0.9 (0.4–2.0)	2,4-D manufacturing; 1985–2007 cohort 2

Burns et al. [79]	SIR	1.3 (0.02–7.2)	0.8 (0.01–4.5)	1.7 (0.9–2.9)	0.9 (0.3–2.0)	2,4-D manufacturing; 1985–2007 cohort 3

SIR: standardized incidence ratio.

SMR: standardized mortality ratio.

**Table 4:** Epidemiologic studies by histological subtype for NHL.

Type of lymphoma	Number exposed	Chlorophenoxys	MCPA	2,4-D and/or 2,4,5-T	Reference
B-cell lymphomas total	819	**1.99 (1.2–2.32)**	**2.59 (1.14–5.91)**	1.69 (0.94–3.01)	Eriksson et al. [71]
	665	1.47 (0.33–6.64)			Fritschi et al. [67]

Lymphocytic	196	2.11 (0.99–4.47)	2.57 (0.74–8.97)	1.93 (0.85–4.41)	Eriksson et al. [71]

Follicular	165	1.26 (0.42–3.75)	—	1.21 (0.35–4.22)	Eriksson et al. [71]
	227	1.15 (0.12–11.2)			Fritschi et al.[67]
	27	0.8 (0.2–3.6)			Orsi et al. [70]

Diffuse large B cell	239	**2.6 (1.08–4.33)**	**3.94 (1.48–10.5)**	1.65 (0.71–3.82)	Eriksson et al. [71]
	231	2.16 (0.36–13.1)			Fritschi et al. [67]
	30	1.0 (0.4–2.8)			Orsi et al. [70]

Other specified B cell	131	**2.60 (1.20–5.64)**	3.2 (0.95–10.7)	2.21 (0.9–5.44)	Eriksson et al. [71]

Unspecified B cell	89	1.14 (0.33–3.95)	1.35 (0.16–11.2)	0.88 (0.2–3.92)	Eriksson et al. [71]

T cell	53	1.62 (0.36–7.25)	2.4 (0.29–20)	1.02 (0.13–7.95)	Eriksson et al. [71]

Unspecified NHL	38	**3.75 (1.16–12.1)**	**9.31 (2.11–41.2)**	3.21 (0.85–12.1)	Eriksson et al. [71]

Hoar et al. [57] reported no differences in the ORs associated with herbicide use when cases were categorized by histological subtype (specific results not reported).

Hardell et al. [56] reported no differences in the ORs associated with herbicide use when cases were categorized by histological subtype (specific results not reported).

**Table 5:** ToxCast assay results for 2,4-D and MCPA.

ToxCast assay	2,4-D	MCPA	Description
	(*μ*M)	
ACEA_LOCinc	1.2		Change in cell growth kinetics.

BSK_SAg_CD38_up		1.5	Novel multifunctional ectoenzyme widely expressed in cells and tissues especially in leukocytes. CD38 also functions in cell adhesion, signal transduction, and calcium signaling. Highly expressed in NHL.

BSK_SM3C_Proliferation_down		1.5	Primary bronchial vascular epithelial smooth muscle cells; proliferation.

BSK_BE3C_IL1a_up	1.5		This interleukin 1 cytokine is a pleiotropic cytokine involved in various immune responses, inflammatory processes, and hematopoiesis.

BSK_BE3C_uPAR_up	1.5		Encodes the receptor for urokinase plasminogen activator.
BSK_BE3C_uPAR_down		40.0	

CLZD_CYP2B6_48		8.4	Encodes a member of the cytochrome P450 superfamily of enzymes.

BSK_LPS_TissueFactor_down	13.3		Encodes coagulation factor III which is a cell surface glycoprotein. This factor enables cells to initiate the blood coagulation cascade.

CLM_MitoticArrest_72 hr	14.5		Mitotic arrest; phosphorylation. Disrupts cell growth in HepG2 cells.

BSK_SM3C_LDLR_down	40.0		Low-density lipoprotein receptor; cholesterol.

CLM_MicrotubuleCSK_Destabilizer_72 hr	57.0		Affects Hep3G cell growth.

ATG_PPRE_CIS		46.0	Transcription factor HepG2. Multiplexed reporter gene assay; nuclear receptor pathway.

ATG_PPARg_TRANS	80.7	40.0	The protein encoded by this gene is PPAR gamma and is a regulator of adipocyte differentiation.

**Table 6:** *In vivo* animal studies.

Organism	Compound	Test	Endpoint	Duration	Doses	Results	Reference
Wistar rat (50 m, 50 f)	MCPA	Oral feeding	Chronic toxicity, oncogenicity	Two years	0, 1, 4, and 16 mg/kg-d	No oncogenicity; systemic NOEL = 4.4 mg/kg-d; LOEL 17.6 mg/kg-d	Bellet et al. [1]

B6C3F1 mice (50 m, 50 f)	MCPA	Oral feeding	Chronic toxicity, oncogenicity	Two years	0, 20, 50, and 500 mg/kg	No oncogenicity; systemic NOEL in females = 3.9 mg/kg-d; LOEL = 19.5 mg/kg-d; systemic NOEL in males = 15.7 mg/kg-d; LOEL = 79.5 mg/kg-d	Bellet et al. [1]

Albino rat (m, f; 25 each)	MCPA	Oral feeding	Reproductive	Two generation	0, 2.5, 7.5, and 22.5 mg/kg-d	Reproductive NOEL = 7.5 mg/kg-d; LOEL = 22.5 mg/kg-d	Bellet et al. [2]

Beagle dog (6 m, 6 f)	MCPA	Oral feeding	chronic toxicity	One year	0, 0.15, 0.75, 3.75 mg/kg-d	Effects in liver and kidney; systemic NOEL = 0.15 mg/kg-d; LOEL = 0.75 mg/kg-d	Reported in US EPA, IRIS [175]; US EPA [3, 4] EC 2005 [30]

Fischer 344 rats	2,4-D salt	Oral feeding	Chronic toxicity, oncogenicity	Two years	0, 5, 75, and 150 mg/kg-d	No effects; NOEL = 5 mg/kg-d	Charles et al. [6]

B6C3F1 mice	2,4-D salt	Oral feeding	Chronic toxicity, oncogenicity	Two years	0, 5, 62.5, and 125 mg/kg-d	No effects	Charles et al. [6]

Fischer 344 rats (m, f, 50 each)	2,4-D acid; salt; ester	Oral feeding	Subchronic toxicity	13 weeks	0, 1, 15, 100, and 300 mg/kg-d	NOEL = 15 mg/kg-d; statistically significant decreases in red cell mass, platelet count, T3, and T4 at 100 mg/kg-d	Charles et al. [7]

5 beagle dogs/sex/group	2,4-D acid; salt; ester	Oral feeding	Chronic toxicity, oncogenicity	One year	0, 1, 5, and 10 mg/kg-d; 10 changed to 7.5 in yr 2	NOEL = 1 mg/kg-d	Charles et al. [176]

Male CD-1 mice	salt; commercial formulation	Drinking water	Effect on spontaneous lymphocytic leukemia	365 days	0 to 50 mg/kg-d	No effects; more control mice died than 2,4-D treated mice	Blakley et al.[177]

Male Fischer 344 rats	salt; commercial formulation	Oral gavage	Immune function	2x week for 4 weeks	10 mg/kg	No impact on lymphocyte blastogenesis	Blakley et al. [178]

Swiss male (6 m)	2,4-D salt; 2,4-DCP	Oral gavage; i.p.	Chromosomal aberrations in bone marrow, germ cells, and sperm cells	Three and five days	1.7, 3.3, and 33 mg/kg	No effects at 1.7; statistically significant chromosomal aberrations in 2/3 mice at 3.3 mg/kg and the one mouse tested at 33 mg/kg	Amer and Aly [179]

12 male CD1 mice per group	2,4-D	Dermal	Bone marrow micronucleus	1 topical application	Approximately 110, 220, and 442 ppm	No effect	Schop et al. [180]

13 male CD1 mice per group	2,4-D	Dermal	Hair follicle nuclear aberration	1 topical application	Approximately 110, 220, and 442 ppm	2% increase in nuclear aberrations at highest applied dose	Schop et al. [180]

5 per dose per group male Han Wistar rat	2,4-D acid; salt; ester; 4 other derivatives	Oral gavage	*In vivo *unscheduled DNA synthesis	One dose	100, 400 ppm	No effects observed	Charles et al. [181]

Chinese hamster	2,4-D acid; MCPA (pure compound and commercial herbicide)	Oral gavage	SCE in lymphocytes	9 days	100 mg/kg-d	Weak increase in SCE; not statistically significant	Linnainmaa [19]

Male Han Wistar rats	2,4-D; MCPA	Oral gavage	SCE rat lymphocytes	5 days wk for 2 weeks	100 mg/kg-d	No increase in SCE	Mustonen et al. [153]

Male Han Wistar rats	2,4-D; MCPA	Oral gavage	SCE rat lymphocytes	5 days wk for 2 weeks	100 mg/kg-d	Significantly increased peroxisome proliferation	Mustonen et al. [153]

Sprague Dawley (5 dose/group)	2,4-D acid; MCPA (pure compound and commercial herbicide)	Oral gavage	SCE in rat lymphocytes	10 days	100, 200 mg/kg-d	No increase in SCE	Linnainmaa [19]

Mice (5 per dose)	2,4-D acid	Single oral gavage	SCE in rat lymphocytes	1 day	50, 100, and 200 mg/kg-d	Statistically significant increase in SCE at 2 highest doses	Madrigal-Bujaidar et al. [182]

Osborne Wendel rats (25/dose/sex)	2,4-D acid	Oral feeding	Chronic toxicity, oncogenicity	Two years	0, 5, 125, 625, and 1250 mg/kg-d	No statistically significant effects	Hansen et al. [183]

Beagle dog (3/dose/sex)	2,4-D acid	Oral feeding	Chronic toxicity, oncogenicity	Two years	0, 10, 50, 100, and 500 mg/kg-d	No statistically significant effects	Hansen et al. [183]

Male Wistar Albino rats	2,4-D acid; MCPA	Oral gavage	P450 induction	Three days	50, 100, and 200 mg/kg	Significant increase in cytochrome P450	Bacher and Gibson [24]

Male Wistar rats	2,4-D salt; MCPA ester	Oral feeding	GST, GSH, and oxidation	14 days	1 mM/kg-d	Weak lipid peroxidation; increase in glutathione reductase activity; increase in glutathione peroxidase for MCPA	Hietanen et al. [184]

**Table 7:** *In vitro* animal studies.

Organism	Compound	Dose	Endpoint	Duration	Results	Reference
Review study of *in vivo* (*n* = 15) and *in vitro* (*n* = 19) studies of mutagenicity and genotoxicity	MCPA	Review and meta-analysis	Mutagenicity and genotoxicity	Many	6 equivocal positive results *in vitro *at highest dose; 6 weakly positive (borderline statistical significance, etc.) *in vivo *	Elliott [20]

Chinese hamster ovary	2,4-D acid; MCPA (pure compound and commercial herbicide)	100, 150, and 200 mg/kg oral gavage	CHO/HGPRT assay	2 weeks 5 days/wk	No increase in SCE from 2,4-D; significant increase in SCE from MCPA	Linnainmaa [19]

Male Wistar rat liver	2,4-D acid	0, 200, and 500 *μ*M (44, 110 ppm)	Liver mitochondrial bioenergetics	Not specified	depressed membrane polarization at highest concentration; no effect on ATP synthetase	Palmeira et al. [159–162]

Male Fischer 344 rat hepatocytes	2,4-D acid; salt; ester; 4 other derivatives	2–350 *μ*g/L	Unscheduled DNA synthesis		No effects observed	Charles et al. [181]

Bacteria	2,4-D acid; salt; ester; 4 other derivatives	10–10000 *μ*g/plate	Ames test		No evidence of mutagenic activity in any tester strain across 2,4-D or any derivative either in the presence or absence of S9	Charles et al. [181]

3–5/dose/sex/group ICR and CD-1 mice	2,4-D acid; salt; ester; 4 other derivatives	40, 130, and 400 ppm	Mouse bone marrow micronucleus test		No significant increases in the incidence of micronucleated polychromatic erythrocytes (MN-PCE)	Charles et al. [185]

Sprague Dawley rat blood	2,4-D 2-butoxyethyl ester, and 2,4-D isopropylamine salt, 2,4-D triisopropanolamine salt	Up to 1400 *μ*g/mL plate	Rat lymphocyte chromosomal aberration test	48 hr	No clastogenic response	Gollapudi et al. [18]

Chinese hamster ovary	2,4-D 2-butoxyethyl ester, 2,4-D isopropylamine salt, 2,4-D triisopropanolamine salt	up to 1400 *μ*g/mL plate	CHO/HGPRT assay	48 hr	No increase in SCE	Gollapudi et al. [18]

Chinese hamster ovary	2,4-D acid; salt	2–10 *μ*g/mL	SCGE (Comet)	90 m/36 hr	Statistically significant increases in DNA damage up to 100%	González et al. [186]

Chinese hamster ovary	2,4-D acid; salt	2–10 *μ*g/mL	Cell cycle progression, mitotic index, and replicative index	90 m/36 hr	No effect on cell cycle progression or replicative index; reductions in mitotic index	González et al. [186]

Chinese hamster ovary	2,4-D acid; salt	2–20 *μ*g/mL	Chromosomal aberrations	90 m/36 hr	Significant dose- dependent increase in SCE	González et al. [186]

Chinese hamster ovary	2,4-D acid; salt	200 *μ*M–4 mM	SCGE (Comet)	24 hr	No effects observed	Sorensen et al. [187]

Syrian hamster embryo	2,4-D salt	1, 2.5, and 5 *μ*g/mL	SCGE (Comet)	5 hr/24 hr	Increase in DNA damage	Maire et al. [188]

Syrian hamster embryo	2,4-D salt	1, 2.5, and 5 *μ*g/mL	Morphological transformation	5 hr/24 hr	Percentage of morphologically transformed colonies increased in a dose-dependent manner to up to 2.4% and 3.8% at 11.5 *μ*M and 23 *μ*M; significant upregulation of c-Myc RNA. No induction of apoptosis	Maire et al. [188]

Chinese hamster ovary cells	2,4-D	1 mM	Polyamine biosynthesis	24 hr	Significant decrease in polyamine metabolism	Rivarola and Balegno [189]; Rivarola et al. [190]

Chinese hamster (lung) V79 cells	2,4-D acid	20, 50, 75, 100, 120, and 140 *μ*g/mL	Intercellular communication	Hours	Colony forming inhibited at 140 *μ*g/mL	Rubinstein et al. [23]

**Table 8:** Studies in human cell cultures *in vitro* or humans exposed *in vivo*.

Test system	Compound	Concentration	Number	Endpoint	Results	Reference	Effect
*In vitro* human lymphocytes	2,4-D acid	0.2, 10, 20, 30, 40, 50, and 60 *μ*g/mL for 48–52 h	NA	SCE and chromosomal aberrations	Statistically significant increase in SCE at all doses (but less at highest dose); deletions and gaps above 50 ug/mL	Korte and Jalal [191]	++

*in vitro* human lymphocytes	2,4-D acid	50, 100, and 250 *μ*g/mL for 72 h	NA	SCE	Significant increase in SCE at lowest dose but not at higher doses; weak effect	Turkula and Jalal [192]	+

*In vitro* human lymphocytes	2,4-D acid; DMA salt (commercial product)	0.125, 0.25, 0.5, 1.0, and 1.25 mM	NA	Chromosomal aberrations	Statistically significant increases in breaks at 0.5 (but not gaps) only in commercial mixture (not pure product)	Mustonen et al. [193]	+

Human fibroblasts	2,4-D acid; DMA salt (commercial product)	18 mM 2,4-D; 5 mM U 46 D Fluid	NA	Colony-forming ability	No effect of 2,4-D; reduced colony-forming ability of U 46 D Fluid at 5 mM	Clausen et al. [194]	+

Human fibroblasts	2,4-D acid; DMA salt (commercial product)	18 mM 2,4-D; 5 mM U 46 D Fluid	NA	Single-strand DNA breaks	No effect of 2,4-D; increased strand breaks of U 46 D Fluid (0.1 strand breaks/10 mM)	Clausen et al. [194]	+

Human fibroblasts	U 46 D Fluid (commercial product)	1 mM–10 mM	NA	Unscheduled DNA synthesis	No effect	Jacobi and Witte [195]	−

Human fibroblasts	U 46 D Fluid (commercial product)	1 mM–10 mM	NA	Colony forming ability	No effect	Jacobi and Witte [195]	−

*In vitro* human erythrocytes	2,4-D salt; 2,4-DP; MCPA	100; 500; 1000 ppm for 1, 3, and 24 h	NA	Catalases in human blood	Small decrease in catalase activity at 1000 ppm	Bukowksa et al. [169]	+

*In vitro* human erythrocytes	MCPA-NA; 2,4-DMP	10–500 ppm	NA	Effect on glutathione (GSH and GSSG), glutathione peroxidase (GSH-Px), glutathione transferase (GST), and adenine energy charge (AEC)	MCPA decreased GSH at 250 ppm (not statistically significant); no effect on GSSG, GSH-Px, GST, and AEC	Bukowska et al. [158]	+

*In vitro* human erythrocytes	2,4-D-NA; MCPA-NA; 2,4-DCP	1–1000 ppm	NA	AChE activity (indicator of membrane damage)	One hour incubation of erythrocytes showed no statistically significant changes in AChE activity except for highest dose of 2,4-D (1000 ppm)	Bukowska and Hutnik 2006 [196]	−

*In vitro* human erythrocytes	2,4-D; MCPA	1, 2, and 4 mM	NA	–SH groups (membrane protein damage)	Membrane damage at 2 and 4 mM	Duchnowicz et al. [165]	

*In vitro* human erythrocytes	2,4-D; MCPA	1, 2, 4 mM	NA	ATPase	Increase in ATPase at 1mM; decrease at 2 and 4 mM	Duchnowicz et al. [165]	+

*In vitro* human erythrocytes	2,4-D-NA; MCPA-NA	0.045 mM to 2.25 mM	NA		2,4-D-NA induced H2DCF oxidation, but not MCPA-NA. Neither denatured hemoglobin	Bukowska et al. [168]	−

*In vitro* human lymphocytes	2,4-D salt	1–3 mM	NA	Apoptosis	Significant increase in apoptosis but only at 660 ppm	Kaioumova et al. [170]	+

*In vitro* HepG2 cells	2,4-D	4, 8, and 16 mM	NA	Apoptosis	Significant increase in apoptosis above 884 ppm	Tuschl and Schwab [197]	+

*In vitro* human blood/lymphocytes from 5 individuals	Commercial product	0.001–1.0 mM	NA	Peripheral blood lymphocyte proliferation (RI); micronuclei frequency (MN)	Minimal increase in MN frequency at cytotoxic level, decreased RI with high dose	Holland et al. [21]	+

*In vitro* human lymphocyte from 15 healthy male smoker/nonsmokers	2,4-D	221–2,221 *μ*g/mL	8 healthy nonsmokers, 7 healthy smokers	SCGE (comet)	No effects observed in nonsmokers; smokers only at the highest concentration	Sandal and Yilmaz [198]	−

*In vitro* human lymphocytes	Deherban A (commercial 2,4-D product)	0.4 and 4 *μ*g/mL	healthy, young nonsmokers	SCE	Statistically significant increase in chromatid and chromosome breaks, number of micronuclei and number of nuclear buds	Zeljezic and Garaj-Vrhovac [199]	++

*In vitro* whole blood culture	2,4-D acid; salt	10, 25, 50, and 100 *μ*g/mL	6 healthy individuals	SCE	Statistically significant increase in SCE at all doses; not at lowest dose for salt	Soloneski et al. [22]	++

*In vitro* plasma leukocyte culture	2,4-D acid; salt	10, 25, 50, and 100 *μ*g/mL	6 healthy individuals	SCE	No effect	Soloneski et al. [22]	−

*In vivo *							

Blood from *in vivo* exposed workers	2,4-D acid; commercial product	Sprayed 333 g/L 2,4-D; 167 g/L MCPA between 6–28 days	19 exposed sprayers	Chromosomal aberrations	No effects observed	Mustonen et al. [193]	−

Blood from *in vivo* exposed workers	2,4-D acid; MCPA	12–155 kg sprayed	10 farmers	Immunological variables	Small,statistically significant reduction in immunological variables 1–12 d but not 50–70 d following exposure. Increased CD8-DR	Faustini et al. [200]	+

Blood from *in vivo* exposed workers	2,4-D acid	Not given; based on urinary concentrations (12–1285 ppb)	13 exposed sprayers	Peripheral blood lymphocyte proliferation (RI); micronuclei frequency (MN)	Small, statistically significant increase in RI not in MN related to urinary levels of 2,4-D but not statistically significant	Figgs et al. [201]	+

Isolated lymphocytes from *in vivo* exposed workers	2,4-D acid; commercial product	Exposure based on urinary levels of 2,4-D	12 exposed sprayers	Peripheral blood lymphocyte proliferation (RI); micronuclei frequency (MN)	No effect on MN, increased RI	Holland et al. [21]	+

*Notes*. ^−^No effect observed; ^+^weak effect (not statistically significant or at conentrations exceeding systemic toxicity); ^++^statistically significant strong effect.

**Table 9:** Summary of evaluations of cellular interactions of 2,4-D.

Study type	Result	Reference
*In vivo *		
Micronucleus formation		
Human lymphocytes	−	Figgs et al. [201]
Human lymphocytes	+	Holland et al. [21]
Mouse bone marrow	−	IARC [29]
Replicative Index		
Human lymphocytes	++	Holland et al. [21]
Human lymphocytes	++	Figgs et al. [201]
Sister chromatid exchange (SCE) and chromosomal aberrations		
Chinese hamster bone marrow	+	Linnainmaa [19]
Rat lymphocytes	−	Linnainmaa [19]
Human lymphocytes	+	Turkula and Jalal [192]
Human lymphocytes	−	Mustonen et al. [193]
Rat lymphocytes	−	Mustonen et al. [153]
Somatic and germ cells in mice	+	Madrigal-Bujaidar et al. [182]
Chicken embryos	++	Arias [212]
Mouse bone marrow and spermatocytes	+	Amer and Aly [179]
Unscheduled DNA Synthesis (UDS)		
Han Wistar rat	−	Charles et al. [181]
Cell transformation/cytotoxicity		
Increased CD8-DR in humans	+	Faustini et al. [200]
*In vitro *		
Micronucleus formation		
Mouse bone marrow	−	Charles et al. [185]
Human lymphocytes	−	Holland et al. [21]
Human lymphocytes	++	Zeljezic and Garaj-Vrhovac [199]
Mouse bone marrow	−	Schop et al. [180]
Sister chromatid exchange (SCE) and chromosomal aberrations		
Human lymphocytes	++	Korte and Jalal [191]
Human lymphocytes	+	Mustonen et al. [193]
Human lymphocytes	++	Zeljezic and Garaj-Vrhovac [199]
Human lymphocytes (SCGE) smoker	+	Sandal and Yilmaz [198]
Human lymphocytes (SCGE) nonsmoker	−	Sandal and Yilmaz [198]
Human fibroblasts	+	Clausen et al. [194]
Rat lymphocytes	−	Mustonen et al. [153]
Rat lymphocytes	−	Gollapudi et al. [18]
Chinese hamster ovary	−	Linnainmaa [19]
Chinese hamster ovary (SCGE)	++	González et al. [186]
Syrian hamster embryo (SCGE)	++	Maire et al. [188]
Chinese hamster ovary (SCGE)	−	Sorensen et al. [187]
Hair follicle nuclear aberration (mouse)	+	Schop et al. [180]
Unscheduled DNA Synthesis (UDS)		
Rat hepatocytes	−	Charles et al. [181]
Human fibroblasts	−	Jacobi and Witti [195]
Replicative index		
Chinese hamster ovary	−	González et al. [186]
Cell transformation		
upregulation of c-Myc RNA in SHE	++	Maire et al. [188]
bcl2 and bax expression in SHE	−	Maire et al. [188]
Colony-forming ability in human fibroblasts	+	Clausen et al. [194]
Polyamine biosynthesis in CHO	+	Rivarola and Balegno [189]
Induction of apoptosis in human lymphocytes	+	Kaioumova et al. [170]
Induction of apoptosis in human lymphocytes	+	Tuschl and Schwab [197]

*Notes*. ^+^Weak effect (not statistically significant or statistically significant but at *in vivo* concentrations exceeding renal clearance mechanisms or systemic toxicity); ^++^significant effect; ^−^no effect.

**Table 10:** Summary of biomonitoring studies.

Reference	Measured	Activity	Urine Concentration (*μ*g/L)	Notes
Frank et al. [213]	Forestry workers involved in aerial application of 2,4-D	During 2 weeks of spraying	High of 323	ND within one week following spraying
		Postspray	High of 326	

Yeary [214]	45 lawn applicators in four areas	Area 1	3.2 (0.1)	Mean (SD)
		Area 2	6.3 (1.8)	Mean (SD)
		Area 3	0.35 (0.5)	Mean (SD)
		Area 4	1.38 (0.5)	Mean (SD)

Lavy et al. [215]	Forestry workers using four different application methods and conventional versus protective equipment	Backpack (conventional)	73.15 (1.51)	Range (35.8–183.7)
		Backpack (protective)	81.09 (1.71)	Range (30.1–244.5)
		Injection bar (conventional)	6.12 (2.48)	Range (1.4–33.6)
		Injection bar (protective)	2.48 (3.28)	Range (0.4–12.1)
		Hypohatchet (conventional)	45.62 (3.14)	Range (1.8–262.9)
		Hypohatchet (protective)	25.14 (2.79)	Range (0.7–103.5)
		Hack-squirt (conventional)	9.08 (5.82)	Range (0.4–140.8)
		Hack squirt (protective)	2.99 (7.48)	Range (0.1–60.3)

Harris et al. [216, 217]	Turf applicators of 2,4-D and bystanders	Bystanders	<4	ND

Knopp [154]	2,4-D production and manufacturing	Production workers	736 (1.3)	GM (GSD)

Figgs et al. [201]	2,4-D in applicators	204 spraying hours	240	Mean (range = 12–1285)

Garry et al. [124]	State licensed pesticide applicators	Backpack	185	GM (GSD not provided; range 28–1700; next highest 890)
		Boom spray	203	GM (GSD not provided; range 86–290)
		Aerial	24	GM (GSD not provided; range ND–97)
		Skidder	12	GM (GSD not provided; range 0.85–58)

Arbuckle et al. [218]	2,4-D in urine of farm applicator	Self-reported use on application day	27.36 (72.48)	Mean (SD). Model explained 39% of variability in urine levels.
	2,4-D in urine of farm applicator	No application	2.58 (7.99)	
	MCPA in urine of farm applicator	Self-reported use on application day	44.9 (110.36)	
	MCPA in urine of farm applicator	No application	1.27 (2.28)	

Hines et al. [219]	2,4-D in urine of applicator	Ground boom application	12.8 (1.7)	GM (GSD)
	2,4-D in reference population	Reference	12	10% detected

Curwin et al. [220]	Nonfarmer	No application	0.29 (3.6)	GM (GSD); *n* = 45

	Farmer	No application	0.48 (4.1)	GM (GSD); *n* = 27
Curwin et al. [220]	Farmer	Application by others	1.6 (7.3)	GM (GSD); *n* = 45
	Farmer	Applicator	13 (7.1)	GM (GSD); *n* = 16

Arbuckle 2006 [221]	2,4-D in urine of farming families in Ontario	Preapplication	2.16 (5.14)	GM (SD) includes MCPA and 2,4-D (ELISA method does not distinguish). Application rates, and so forth. not specified.
		24 hrs post application	9.10 (5.02)	
		48 hrs post application	8.55 (5.68)	
		Spouse preapplication	1.10 (3.48)	
		Spouse 24 hrs post	0.93 (3.21)	
		Spouse 48 hours post	1.08 (4.84)	
		Child preapplication	1.44 (3.65)	
		Child 24 hrs post	0.90 (4.32)	
		Child 48 hrs post	1.46 (2.32)	

Acquavella et al. [222]	Farm family exposure study to 2,4-D	Self-reported use on application day; application method not reported	64	GM (GSD not provided; range 2–1856)

Alexander et al. [223]	Farm families in Minnesota and South Carolina	Applicator	71.9 (6.2)	GM (GSD); range 1.5–2236
		Spouse	1.7(3.2)	Range 0.5–24.9
		Children	4.9 (4.5)	Range ND–640.6

Bhatti et al. [224]	31 seasonal 2,4-D applicators	Noxious weed control for 12 weeks	259.4 (431.7)	Mean (SD)
	31 seasonal 2,4-D applicators	Noxious weed control for 12 weeks	63.4 (8.1)	GM (GSD)

Thomas et al. [225]	Agricultural Health Study	Preapplication	7.8 (4.7)	GM (GSD); min ND–max = 210
		Day 1	25 (4.1)	GM (GSD); min 1.6–max = 970
		Day 2	26 (3.7)	GM (GSD); min 2.2–max = 1000
		Day 3	23 (4.1)	GM (GSD); min 1.3–max = 840
		Day 4	27 (4.40	GM (GSD); min 1.1–max = 1700
		Day 5	17 (5.5)	GM (GSD); min 0.8–max = 2500

Morgan et al. [226]	General public, child	Child (NC)	50th-0.5, 95th-1.9	*n* = 66, 88% detectable levels
	General public, child	Child (OH)	50th-1.2, 95th-4.3	*n* = 69, 97% detectable levels
	General public, adult	Adult (NC)	50th-0.7, 95th-2.8	*n* = 66, 86% detectable levels
	General public, adult	Adult (OH)	50th-0.7, 95th-3.3	*n* = 69, 87% detectable levels

Aylward et al. [26] (NHANES)	General public, child	6–11 years	50th-0.2, 95th-1.55	50th = detection level
	General public, young adult	12–19 years	50th-0.2, 95th-1.4	50th = detection level
	General public, adult	20–59 years	50th-0.2, 95th-1.24	50th = detection level

ExpoCastDB 2,4-D [227]	General public NC	Unknown	0.5 (2.66)	GM (GSD); range 0.23–6.65.
	General public OH		0.8 (2.79)	GM (GSD); range 0.44–42.9

**Table 11:** Oral equivalent doses based on ToxCast dosimetry and estimated urinary levels associated with oral doses.

2,4-D	BW (kg)	24 hr urine volume (L)	*In vitro-in vivo* equivalent dose (mg/kg-d)	Scenario	Estimated urine level from oral dose (*μ*g/L)
Children 4–12	30	0.66	0.014	Lowest biologically relevant ToxCast assay result; highest predicted *C*_ss_	636
Adolescents	55	1.7	0.014		453
Women	55	1.6	0.014		481
Men	70	1.7	0.014		576

Children 4–12	30	0.66	0.028	Lowest biologically relevant ToxCast assay result; average predicted *C*_ss_	1273
Adolescents	55	1.7	0.028		906
Women	55	1.6	0.028		963
Men	70	1.7	0.028		1153

MCPA					

Children 4–12	30	0.66	0.01	Lowest biologically relevant ToxCast assay result; highest predicted *C*_ss_	455
Adolescents	55	1.7	0.01		324
Women	55	1.6	0.01		344
Men	70	1.7	0.01		412

Children 4–12	30	0.66	0.0005	Oral RfD	23
Adolescents	55	1.7	0.0005		16
Women	55	1.6	0.0005		17
Men	70	1.7	0.0005		21

Children 4–12	30	0.66	0.0044	Oral RfD used by Health Canada; OPPTS	200
Adolescents	55	1.7	0.0044		142
Women	55	1.6	0.0044		151
Men	70	1.7	0.0044		181
