# Supplementary material for: A Systematic Review of Carcinogenic Outcomes and Potential Mechanisms from Exposure to 2,4-D and MCPA in the Environment
Source: J Toxicol. 2013 Feb 26;2013:371610. doi: 10.1155/2013/371610 (PMC3600329; doi:10.1155/2013/371610)
Supplement: Supplementary file 1 — Supplementary materials provide the genetic analysis profile (GAP) developed by IARC to evaluate mutagenicity of 2,4-D [10, 29, 45]. [file 371610.f1.pdf]

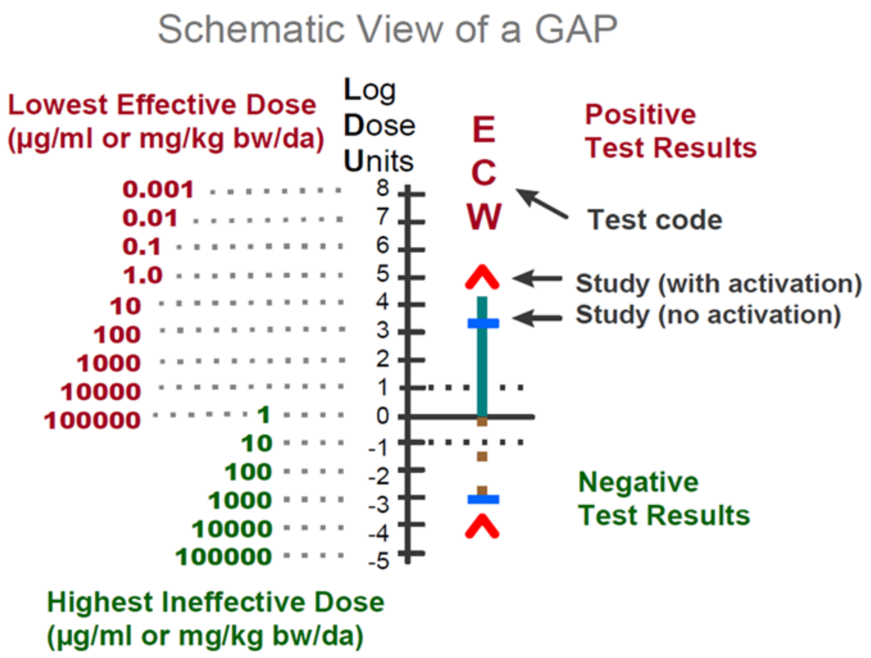

In the schematic diagram of a GAP above, four studies (two positive; two negative) are illustrated for an example short-term test, ECW. Studies using exogenous metabolic activation are indicated by the carets (^) and those studies done without exogenous activation are marked by the horizontal bars (-). A solid vertical profile line is drawn to the mean LDU of the majority call (positive or negative). A dashed vertical line is drawn to the extreme of the conflicting test results. Note in cases where there are an equal number of positive and negative studies, as illustrated here, the overall call is positive.

GAPs are produced with tests in phylogenetic order and with tests in endpoint order (see below for 2,4-D).

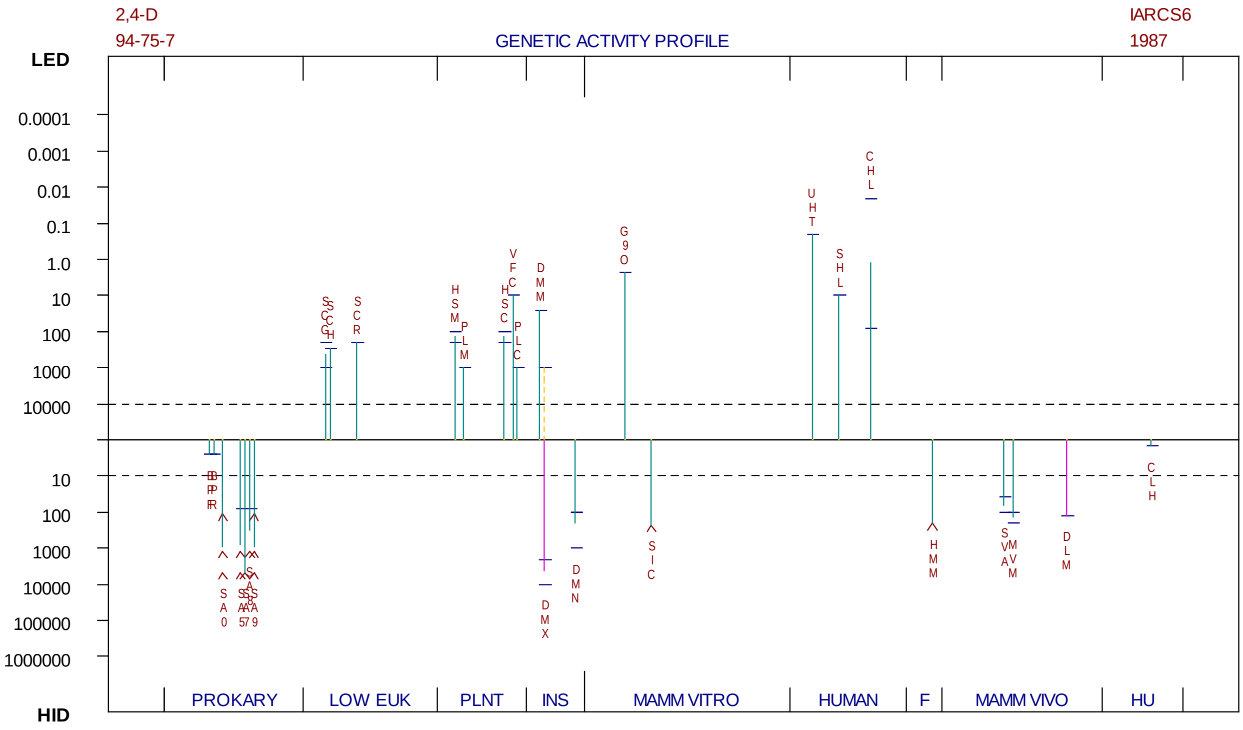

IARC possible human carcinogen (group 2B*: human - limited, animal - inadequate)

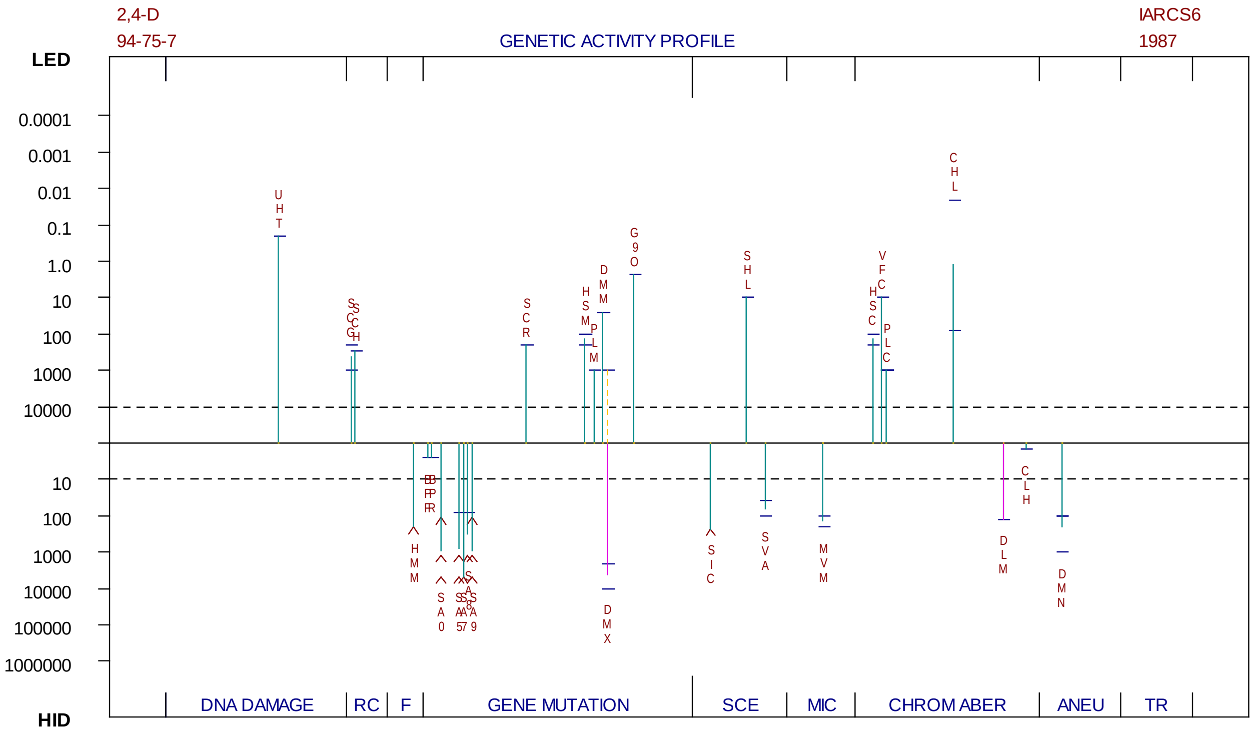

IARC possible human carcinogen (group 2B*: human - limited, animal - inadequate)

TEST CODE	END-POINT	RESULTS -MA	RESULTS +MA	DOSE (LED or HID)	REF ID NUMBER	REFERENCE
1 UHT	D	+	+	0.2000	2756	Ahmed et al. (1977)
2 SCG	R	+	0	200.0000	3566	Zetterberg et al. (1977)
3 SCG	R	(+)	0	1000.0000	2339	Siebert & Lemperle (1974)
4 SCH	R	+	0	300.0000	3566	Zetterberg et al. (1977)
5 HMM	H	0	-	200.0000	3566	Zetterberg et al. (1977)
6 BPF	G	-	0	2.5000	10066	Andersen et al. (1972)
7 BPR	G	-	0	2.5000	10066	Andersen et al. (1972)
8 SA0	G	-	-	5000.0000	3718	Mortelmans et al. (1984)
9 SA0	G	-	-	110.0000	2308	Nishimura et al. (1982)
10 SA0	G	0	-	1250.0000	47	Anderson & Styles (1978)
11 SA5	G	-	-	5000.0000	3718	Mortelmans et al. (1984)
12 SA5	G	-	0	80.0000	3566	Zetterberg et al. (1977)
13 SA5	G	0	-	1250.0000	47	Anderson & Styles (1978)
14 SA7	G	-	-	5000.0000	3718	Mortelmans et al. (1984)
15 SA8	G	-	0	80.0000	3566	Zetterberg et al. (1977)
16 SA8	G	0	-	1250.0000	47	Anderson & Styles (1978)
17 SA9	G	-	-	5000.0000	3718	Mortelmans et al. (1984)
18 SA9	G	-	-	110.0000	2308	Nishimura et al. (1982)
19 SA9	G	0	-	1250.0000	47	Anderson & Styles (1978)
20 SAS	G	-	0	0.0000	10066	Andersen et al. (1972)

21	SCR	G	+	0	200.0000	3565	Zetterberg (1978)
22	HSM	G	+	0	200.0000	2335	Mohandas & Grant (1972)
23	HSM	G	(+)	0	100.0000	2282	Khalatkar & Bhargava (1982)
24	PLM	G	+	0	1000.0000	2191	Logvinenko & Morgun (1978)
25	DMM	G	+	0	25.0000	3564	Rasmuson & Svahlin (1978)
26	DMX	G	-	0	10000.0000	3719	Zimmering et al. (1985)
27	DMX	G	+	0	1000.0000	2205	Magnusson et al. (1977)
28	DMX	G	-	0	1989.0000	2123	Vogel & Chandler (1974)
29	G9O	G	+	0	2.2000	2213	Ahmed et al. (1977)
30	SIC	S	-	-	221.0000	2052	Linnainmaa (1984)
31	SHL	S	+	0	10.0000	10078	Korte & Jalal (1982)
32	SVA	S	(-)	0	40.0000	3716	Lamb IV et al. (1981)
33	SVA	S	-	0	100.0000	2052	Linnainmaa (1984)
34	SLH	S	(+)	0	0.0000	3717	Crossen et al. (1978)
35	SLH	S	-	0	0.0000	10076	Linnainmaa (1983)
36	MVM	M	-	0	100.0000	2383	Jenssen & Renberg (1976)
37	MVM	M	-	0	200.0000	3563	Seiler (1978)
38	HSC	C	+	0	200.0000	2202	Singh et al. (1977)
39	HSC	C	(+)	0	100.0000	2282	Khalatkar & Bhargava (1982)
40	VFC	C	+	0	10.0000	2324	Prasad & Das (1977)
41	PLC	C	+	0	1000.0000	2222	Al-najjar & Soliman (1982)
42	PLC	C	+	0	1000.0000	2191	Logvinenko & Morgun (1978)
43	CHL	C	+	0	77.3500	3567	Mustonen et al. (1986)
44	CHL	C	+	0	0.0200	10080	Pilinskaya (1974)
45	DLM	C	-	0	125.0000	414	Epstein et al. (1972)

46	CLH	C	(-)	0	0.0000	3712	Hogstedt et al. (1980)
47	CLH	C	-	0	1.5600	3567	Mustonen et al. (1986)
48	DMN	A	-	0	1000.0000	2300	Woodruff et al. (1983)
49	DMN	A	-	0	100.0000	2205	Magnusson et al. (1977)
50	DMN	A	-	0	100.0000	2001	Ramel & Magnusson (1979)
51	ICR		+	0	150.0000	3736	Rubinstein et al. (1984)

IARC possible human carcinogen (group 2B*: human - limited, animal - inadequate)

Footnotes:

Results are recorded in separate data columns for in vitro studies without exogenous metabolic activation (-MA) and/or exogenous metabolic activation (+MA). Results from in vivo studies are recorded in the -MA column.

Codes used for test results are defined as: +, positive; (+), weakly positive; -, negative; ?, equivocal; 0, not tested

Doses are either the lowest effective dose (LED) or highest ineffective dose (HID). Doses from in vitro studies are expressed as µg/ml, and the in vivo values are calculated to be mg/kg body weight per day.

For an agent in an IARC Monographs project, the IARC Working Group determined the degree of evidence of carcinogenicity in humans and animals, and made an overall evaluation of carcinogenic risk to humans. The evaluation is shown immediately above these footnotes for the IARC data. An Asterisk(*) marked after the IARC carcinogenicity evaluation indicates that the evaluation applies to the group of chemicals as a whole and not to any specific chemical within that group.

Description of Test Codes in Endpoint order

DNA damage

UHT = Unscheduled DNA synthesis, transformed human cells in vitro

Recombination

SCG = Saccharomyces cerevisiae, gene conversion

SCH = S. cerevisiae, homozygosis by recombination or gene conversion

Body fluid and host mediated assays

HMM = Host mediated assay, microbial cells in animal hosts

Gene mutation

BPF = Bacteriophage, forward mutation

BPR = Bacteriophage, reverse mutation

SA0 = Salmonella typhimurium TA100, reverse mutation

SA5 = Salmonella typhimurium TA1535, reverse mutation

SA7 = Salmonella typhimurium TA1537, reverse mutation

SA8 = Salmonella typhimurium TA1538, reverse mutation

SA9 = Salmonella typhimurium TA98, reverse mutation

SAS = Salmonella typhimurium (other misc. strains), reverse mutation

SCR = Saccharomyces cerevisiae, reverse mutation

HSM = Hordeum species, mutation

PLM = Plants (other), mutation

DMM = Drosophila melanogaster, somatic mutation (and recombination)

DMX = Drosophila melanogaster, sex-linked recessive lethal mutation

G9O = Gene mutation, Chinese hamster lung V-79 cells in vitro, ouabain

Sister chromatid exchange

SIC = Sister chromatid exchange, Chinese hamster cells in vitro

SHL = Sister chromatid exchange, human lymphocytes in vitro

SVA = Sister chromatid exchange, animal cells in vivo

SLH = Sister chromatid exchange, human lymphocytes in vivo

Micronuclei

MVM = Micronucleus test, mice in vivo

Chromosome aberration

HSC = Hordeum species, chromosomal aberrations

VFC = Vicia faba, chromosomal aberrations

PLC = Plants (other), chromosomal aberrations

CHL = Chromosomal aberrations, human lymphocytes in vitro

DLM = Dominant lethal test, mice

CLH = Chromosomal aberrations, human lymphocytes in vivo

Aneuploidy

DMN = Drosophila melanogaster, aneuploidy

Alternative testcodes (not included in profiles)

ICR = Inhibition of intercellular communication, rodent in vitro

Endpoint Codes

A, Aneuploidy; C, Chromosome aberrations; D, DNA damage; F, Body fluids; G, Gene Mutation

H, Host-mediated assay; M, Micronucleus; R, Recombination; S, SCE; T, Cell transformation

Ahmed, F. E., Hart, R. W., & Lewis, N. J. (1977) Pesticide induced DNA damage and its repair in cultured human cells. Mutat Res, 42, 161-174

(Ref. ID: 2756)

Ahmed, F. E., Lewis, N. J., & Hart, R. W. (1977) Pesticide induced ouabain resistant mutants in Chinese hamster V79 cells. Chem Biol Interact, 19, 369-374

(Ref. ID: 2213)

Al-najjar, N. R. & Soliman, A. S. (1982) Cytological effects of herbicides. I. Effect of 2,4-d, and 2,4,5-T on meiotic cells of wheat and two related species. Cytologia, 47, 53-61

(Ref. ID: 2222)

Andersen, K. J., Leighty, E. G., & Takahashi, M. T. (1972) Evaluation of herbicides for possible mutagenic properties. J Agric Fd Chem, 20, 649-656

(Ref. ID: 10066)

Anderson, D. & Styles, J. A. (1978) An evaluation of six short-term tests for detecting organic chemical carcinogens. Appendix 2. The bacterial mutation test. Br J Cancer, 37, 924-930

(Ref. ID: 47)

Crossen, P. E., Morgan, W. F., Horan, J. J., & Stewart, J. (1978) Cytogenetic studies of pesticide and herbicide sprayers. N.Z. Med. J., 88, 192-195

(Ref. ID: 3717)

Epstein, S. S., Arnold, E., Andrea, J., Bass, W., & Bishop, Y. (1972) Detection of chemical mutagens by the dominant lethal assay in the mouse. Toxicol Appl Pharmacol, 23, 288-325

(Ref. ID: 414)

Hogstedt, B., Kolnig, A., Mitelman, F., & Skerfving, S. (1980) Cytogenetic study of pesticides in agricultural work. Hereditas, 92, 177-178

(Ref. ID: 3712)

Jenssen, D. & Renberg, L. (1976) Distribution and cytogenetic test of 2,4-D and 2,4,5-T phenoxyacetic acids in mouse blood tissues. Chem Biol Interact, 14, 291-299

(Ref. ID: 2383)

Khalatkar, A. S. & Bhargava, Y. R. (1982) 2,4-dichlorophenoxyacetic acid - a new environmental mutagen. Mutat Res, 103, 111-114

(Ref. ID: 2282)

Korte, C. & Jalal, S. M. (1982) 2,4-D induced clastogenicity and elevated rates of sister chromatid exchanges in cultured human lymphocytes. J Hered, 73, 224-226

(Ref. ID: 10078)

Lamb IV, J. C., Marks, T. A., Gladen, B. C., Allen, J. W., & Moore, J. A. (1981) Male fertility, sister chromatid exchange, and germ cell toxicity following exposure to mixtures of chlorinated phenoxy acids containing 2,3,7,8-tetrachlorodibenzo-p-dioxin. J Toxicol Environ Health, 8, 825-834

(Ref. ID: 3716)

Linnainmaa, K. (1983) Sister chromatid exchanges among workers occupationally exposed to phenoxy acid herbicides 2,4-D and MCPA. Teratogenesis Carcinog Mutagen, 3, 269-279

(Ref. ID: 10076)

Linnainmaa, K. (1984) Induction of sister chromatid exchanges by the peroxisome proliferators 2,4-D, MCPA, and clofibrate in vivo and in vitro. Carcinogenesis, 5, 703-707

(Ref. ID: 2052)

Logvinenko, V. F. & Morgun, V. V. (1978) Study of the mutagenic action of certain pesticides on spring Durum wheat. Cytol Genet, 12, 12-16

(Ref. ID: 2191)

Magnusson, J., Ramel, C., & Eriksson, A. (1977) Mutagenic effects of chlorinated phenoxyacetic acids in Drosophila melanogaster. Hereditas, 87, 121-123

(Ref. ID: 2205)

Mohandas, T. & Grant, W. F. (1972) Cytogenetic effects of 2,4-D and amitrole in relation to nuclear volume and DNA content in some higher plants. Can. J. Genet. Cytol., 14, 773-783

(Ref. ID: 2335)

Mortelmans, K., Haworth, S., Speck, W., & Zeiger, E. (1984) Mutagenicity testing of agent orange components and related chemicals. Toxicol Appl Pharmacol, 75, 137-146

(Ref. ID: 3718)

Mustonen, R., Kangas, J., Vuojolahti, P., & Linnainmaa, K. (1986) Effects of phenoxyacetic acids on the induction of chromosome aberrations in vitro and in vivo. Mutagenesis, 1, 241-245

(Ref. ID: 3567)

Nishimura, N., Nishimura, H., & Oshima, H. (1982) Survey on mutagenicity of pesticides by the Salmonella-microsome test. Aichi Ika Daig Igak Zass, 10, 305-312

(Ref. ID: 2308)

Pilinskaya, M. A. (1974) Cytogenetic effect of the herbicide 2,4-D on human and animal chromosomes. Cytol Genet, 8, 6-10

(Ref. ID: 10080)

Prasad, G. & Das, K. (1977) Effects of some growth substances on mitosis. Cytologia, 42, 323-329

(Ref. ID: 2324)

Ramel, C. & Magnusson, J. (1979) Chemical induction of nondisjunction in Drosophila. Environ Health Perspect, 31, 59-66

(Ref. ID: 2001)

Rasmuson, B. & Svahlin, H. (1978) Mutagenicity tests of 2,4-dichlorophenoxyacetic acid and 2,4,5-trichlorophenoxyacetic acid in genetically stable and unstable strains of Drosophila melanogaster. Ecol. Bull. (Stockholm), 27, 190-192

(Ref. ID: 3564)

Rubinstein, C., Jone, C., Trosko, J. E., & Chang, C. (1984) Inhibition of intercellular communication in cultures of Chinese hamster V79 cells by 2,4-dichlorophenoxyacetic acid and 2,4,5-trichlorophenoxyacetic acid. Fundam Appl Toxicol, 4, 731-739

(Ref. ID: 3736)

Seiler, J. P. (1978) The genetic toxicology of phenoxy acids other than 2,4,5-T. Mutat Res, 55, 197-226

(Ref. ID: 3563)

Siebert, D. & Lemperle, E. (1974) Genetic effects of herbicides: induction of mitotic gene conversion in Saccharomyces cerevisiae. Mutat Res, 22, 111-120

(Ref. ID: 2339)

Singh, B. D., Singh, Y., Singh, R. B., Singh, V. P., Singh, R. M., & Bhatnagar, P. S. (1977) Cytogenetic effects of seed treatment with IAA, NAA and 2,4-d in barley Hordeum vulgare L. Indian J Exp Biol, 15, 1105-1108

(Ref. ID: 2202)

Vogel, E. & Chandler, J. L. R. (1974) Mutagenicity testing of cyclamate and some pesticides in Drosophila melanogaster. Experientia, 30, 621-623

(Ref. ID: 2123)

Woodruff, R. C., Phillips, J. P., & Irwin, D. (1983) Pesticide-induced complete and partial chromosome loss in screens with repair-defective females of Drosophila melanogaster. Environ Mutagen, 5, 835-846

(Ref. ID: 2300)

Zetterberg, G. (1978) Genetic effects of phenoxy acids on microorganisms. In: Ramel, C. (Ed.), Chlorinated Phenoxy Acids and Their Dioxins. Ecol. Bull. (Stockholm), 27, 193-204

(Ref. ID: 3565)

Zetterberg, G., Busk, L., Elovson, R., Starec-Nordenhammar, I., & Ryttman, H. (1977) The influence of ph on the effects of 2,4-D (2,4-dichlorophenoxyacetic acid Na salt) on Saccharomyces cerevisiae and Salmonella typhimurium. Mutat Res, 42, 3-18

(Ref. ID: 3566)

Zimmering, S., Mason, J. M., Valencia, R., & Woodruff, R. C. (1985) Chemical mutagenesis testing in Drosophila II. Results of 20 coded compounds tested for the National Toxicology Program. Environ Mutagen, 7, 87-100

(Ref. ID: 3719)
